# scLEGA: an attention-based deep clustering method with a tendency for low expression of genes on single-cell RNA-seq data

**DOI:** 10.1093/bib/bbae371

**Published:** 2024-07-26

**Authors:** Zhenze Liu, Yingjian Liang, Guohua Wang, Tianjiao Zhang

**Affiliations:** Aulin College, Northeast Forestry University 150040, 26 Hexing Road, Xiangfang District, Harbin, China; Key Laboratory of Hepatosplenic Surgery, Ministry of Education, Department of General Surgery, the First Affiliated Hospital of Harbin Medical University 150001, 23 Postal Street, Nangang District, Harbin, China; College of Computer and Control Engineering, Northeast Forestry University 150040, 26 Hexing Road, Xiangfang District, Harbin, China; Faculty of Computing, Harbin Institute of Technology 150006, 92 West Dazhi Street, Nangang District, Harbin, China; College of Computer and Control Engineering, Northeast Forestry University 150040, 26 Hexing Road, Xiangfang District, Harbin, China

**Keywords:** scRNA-seq, multi-head attention mechanism, DAE, GAE

## Abstract

Single-cell RNA sequencing (scRNA-seq) enables the exploration of biological heterogeneity among different cell types within tissues at a resolution. Inferring cell types within tissues is foundational for downstream research. Most existing methods for cell type inference based on scRNA-seq data primarily utilize highly variable genes (HVGs) with higher expression levels as clustering features, overlooking the contribution of HVGs with lower expression levels. To address this, we have designed a novel cell type inference method for scRNA-seq data, termed scLEGA. scLEGA employs a novel zero-inflated negative binomial (ZINB) loss function that fully considers the contribution of genes with lower expression levels and combines two distinct scRNA-seq clustering strategies through a multi-head attention mechanism. It utilizes a low-expression optimized denoising autoencoder, based on the novel ZINB model, to extract low-dimensional features and handle dropout events, and a GCN-based graph autoencoder (GAE) that leverages neighbor information to guide dimensionality reduction. The iterative fusion of denoising and topological embedding in scLEGA facilitates the acquisition of cluster-friendly cell representations in the hidden embedding, where similar cells are brought closer together. Compared to 12 state-of-the-art cell type inference methods on 15 scRNA-seq datasets, scLEGA demonstrates superior performance in clustering accuracy, scalability, and stability. Our scLEGA model codes are freely available at https://github.com/Masonze/scLEGA-main.

## Introduction

Cells, with their diverse types and functions, contribute to the heterogeneity observed in histology [1]. The primary advantage of scRNA-seq is that it allows for the study of cellular gene expression profiles at the single-cell level, enabling more precise measurements of gene expression levels, the discovery of cellular heterogeneity and dynamic changes, the identification of unknown or rare cell types, the elucidation of cellular developmental trajectories and differentiation processes, and the exploration of cellular responses and regulatory mechanisms under conditions such as disease, drugs, and environment [2]. The classification of cell types forms the basis for downstream research [3], which includes analyzing interactions between different cell types and analyzing associations with specific diseases. For many downstream analyses, accurately identifying cell subpopulations is crucial [4].

In recent years, the study of cell type inference based on scRNA-seq data has witnessed the proposal of a plethora of clustering methodologies. For instance, SC3 [5] employs multiple distance metrics and dimensionality reduction techniques to perform repeated clustering of single-cell RNA-seq data (scRNA-seq), subsequently integrating the various clustering outcomes through a consensus clustering algorithm. SIMLR [6], fundamentally a spectral clustering method, achieves effective clustering of single-cell data by learning a reliable distance metric that combines multiple kernel functions. Seurat4 [7], an advanced toolkit for single-cell data analysis, is capable of efficiently processing ultra-large datasets comprising millions of cells. It rapidly constructs a multimodal k-nearest neighbor graph through dimensionality reduction, clustering, and integration methods. Seurat4 also utilizes graph-based community detection algorithms, such as Louvain [8] or Leiden [9], to identify cell subpopulations and visualize cellular distribution and heterogeneity through PCA, t-SNE, or UMAP [10]. Shared nearest neighbors (SNN)-cliq [11], predicated on the concept of SNN, constructs a graph reflecting cellular similarity and then employs a quasi-fluid model to identify subgraphs with high density and connectivity as candidate sets for cell types.

With the continuous advancement of deep learning techniques [12], a variety of deep learning-based clustering methods have been developed to tackle the challenges of cell type inference based on scRNA-seq data. Renowned for their exceptional data fitting capabilities, these methods can reveal hidden information within the data through complex functional models. The end-to-end learning paradigm of deep learning eschews reliance on traditional prior knowledge, enabling the automatic extraction of valuable features directly from raw data. This approach aligns well with the characteristics of scRNA-seq data—high dimensionality and sparsity—making deep learning an ideal tool for uncovering the underlying biological mechanisms [13]. scVI [14] is based on a hierarchical Bayesian model, with conditional distributions specified by deep neural networks, which can be trained very efficiently even for very large datasets. scDeepCluster [15], by integrating a zero-inflated negative binomial [16] (ZINB) distribution autoencoder with DEC [17], has achieved efficient processing of scRNA-seq data. DCA [18] extends the typical autoencoder approach and defines the reconstruction error as the likelihood of the distribution of the noise model instead of reconstructing the input data themselves. DeepScena [19] employs a NB-based autoencoder by fitting the NB model to accomplish data imputation and improve accuracy. scCAN [20], utilizing autoencoders and network fusion techniques, can accurately distinguish different cell types within high-dimensional, sparse scRNA-seq data. DESC [21] employs an autoencoder to learn a low-dimensional representation of the data, followed by soft assignment using deep embedding clustering, and ultimately optimizes cluster centers and data allocation. scGNN [22] introduces a cell–cell graph into multiple autoencoders integrated with a graph convolutional network (GCN) to learn the embedding of the topological graph. scGPCL [23] is also a graph-based method that uses prototypical contrastive learning, aiming at clustering cells in the scRNA-seq data, which fully leverages the relational information between cells. scGAC [24] employs a graph autoencoder (GAE) based on a graph attention network to learn the low-dimensional embedding of cells from the cell–cell graph. scBGEDA [25] is a deep single-cell clustering model that employs a dual denoising autoencoder (DAE) with bipartite graph ensemble clustering.

**Figure 1:**
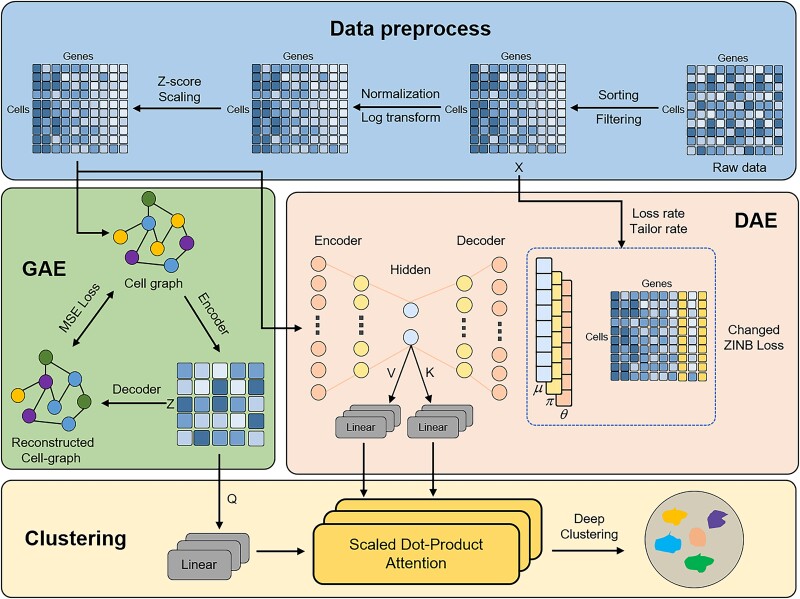
**The overall structure of scLEGA.** scLEGA, through the implementation of changed ZINB loss, is able to focus on the contributions of genes with lower expression levels to cell type inference. This loss function is integrated into a DAE to learn denoising embedding. Subsequently, multi-head attention is employed to combine the denoising embedding produced by the DAE with the topological embedding generated by the GAE for deep clustering purposes.

Current methodologies for cell type inference based on scRNA-seq data predominantly utilize highly variable genes (HVGs) with higher expression levels as the main feature for clustering, overlooking the contribution of HVGs with relatively lower expression levels. These lower-expressed genes may be crucial markers for cell types or states, or key factors in regulatory networks. Therefore, relying solely on highly expressed genes may neglect the uniqueness of certain cell types, thereby affecting the accuracy and completeness of classification. It is necessary to consider both high-expression and low-expression HVGs because high-expression genes provide basic functional and state information of the cells, while low-expression HVGs capture subtle differences and specific characteristics between cells. Combining information from both types of genes can improve the accuracy of clustering and classification, allowing the model to more accurately identify and distinguish cell types. To address this, we have designed a novel cell type inference method based on scRNA-seq data, termed scLEGA. scLEGA introduces a novel ZINB loss function, which adequately considers the importance of HVGs with lower expression levels in cell type inference, leading to a friendlier clustering representation. Additionally, scLEGA employs a multi-head attention mechanism [26] that organically integrates two complementary clustering strategies. One strategy is based on a low-expression optimized DAE with a ZINB model, which extracts biologically meaningful low-dimensional features from high-dimensional data, handles common dropout events in scRNA-seq data, and combines the new ZINB loss to fully consider the contribution of lowly expressed genes to cell type inference. The other strategy is based on a GCN-based GAE approach, which uses similarity information between cells to construct a graph structure and guides the dimensionality reduction process through neighbor information, thus preserving the topological structure of the data. Through iterative fusion based on denoising and topological embedding, scLEGA generates more compact and robust cell representations that are more easily recognized by clustering algorithms. Moreover, scLEGA utilizes the Leiden algorithm to determine initial clustering centers and adaptively labels cells based on their position in the hidden space, without prior knowledge of the number of groups. Many traditional clustering methods, such as k-means, require a predetermined number of clusters, which can lead to inaccurate results in the absence of prior knowledge. Compared to other community detection algorithms, such as the Louvain algorithm, the Leiden algorithm can produce higher quality community partitions, avoiding issues of community splitting and disconnection. The overall framework of the scLEGA method is illustrated in Fig. 1. To evaluate the performance of scLEGA, we compared it with 12 state-of-the-art baseline methods across 15 datasets, demonstrating scLEGA’s advantages in analyzing scRNA-seq data. Compared to several k-means-based methods that require a predetermined number of groups, scLEGA also achieved better results. Experiments also showed the robustness and stability of scLEGA when subjected to manual dropout, indicating the framework’s strong generalization ability and high fault tolerance.

## Methods

### Data Preprocessing

We used the Python package SCANPY [27] to preprocess the raw scRNA-seq data. The scRNA-seq data are structured as a matrix, with rows representing cells and columns representing genes, each cell containing an equal number of genes. Initially, we filtered out cells or genes without counts. Multiple methods [20, 22, 24, 25] have selected HVGs, from 2000 to 3000; Most studies significantly reduce computational costs by selecting an appropriate number of genes without causing a noticeable drop in accuracy, as selecting too many genes can introduce a substantial amount of noise. Therefore, we conducted a comparison to determine the optimal number of genes to select. From Fig. S1, we found that the ARI value was higher when using 2500 genes. Additionally, the performance variability was lower with this selection, indicating stability and reliability. And when the number of genes was increased to 3000 or more, the ARI value did not significantly improve and might even decrease, suggesting that too many genes could introduce noise rather than enhance the results. Therefore, selecting 2500 genes strikes a good balance between performance and computational efficiency, leading us to choose 2500 HVGs as characteristic genes in this study. These were then sorted by gene content magnitude to facilitate the subsequent processing of genes with lower expression levels, forming the original count matrix $X$. Following the normalization and log transformation of each cell, the data were employed to construct a cell–cell graph for input into the GAE. Numerous previous methods [23, 28, 29] have selected 10–20 nearest neighbors to construct the cell–cell graph; in this paper, the similarity of the 15 nearest neighbors for each cell was calculated and filtered using a Gaussian kernel function and cosine distance, which was then used to build a directed graph representing the cell–cell graph. Finally, genes were normalized to a z-score data with a mean of zero and a variance of one, denoted as $\overline{X}$.

### Changed ZINB loss

The ZINB distribution serves as an approximate distribution for scRNA-seq data [15, 18, 30], effectively addressing its sparsity and over-dispersion characteristics, thereby enhancing the accuracy of cell type inference and biological interpretability.

Given that HVGs with lower expression levels in scRNA-seq data may significantly impact clustering, this study employs a DAE based on the ZINB distribution, with a modified ZINB loss by introducing two hyperparameters to adjust the proportion and tendency of low-expressed HVGs. Lowly expressed high-variable genes often represent unique biomarkers of rare or specific cell types. Despite their low expression levels, their high variability can reflect biological differences between cells. Therefore, focusing on these genes helps in more accurately identifying and classifying rare or special cell types. On the other hand, reducing the weight of highly expressed genes may result in the loss of potentially important information within these genes, affecting the integrity of the data. This approach allows the model to consider the contributions of both high and low expression genes, increasing the weight for HVGs with low expressions, and optimizing the clustering of scRNA-seq data. In contrast to the standard ZINB loss, the loss function calculation expands the representation of genes with lower expression levels from the original count matrix $X$ to ${X}^{\prime }$, biasing the model towards these genes and enabling the learning of more rational features for cell type inference. The following two parameters are defined: the tailor rate, which indicates the proportion of genes with higher expression, and the loss rate, which denotes the magnification factor for genes with lower expression. And processed through two hyperparameters, to obtain ${X}_{n\times m}^{\prime }$, as follows, note that we did not modify the original data, but used ${X}^{\prime }$ when calculating ZINB loss:


$$ \kern-2.6pc k= tailor\ rate\times m $$

$$ {X_{ij}}^{\prime }=\left\{\begin{array}{@{}cc}{X}_{ij}& \mathrm{if}\ j\le k\\{} loss\ rate\cdotp{X}_{ij}& \mathrm{if}\ j>k\end{array}\right. $$

As this was the inaugural introduction of such parameters, which are floating-point numbers, it was challenging to determine their reasonable values through repeated experimentation. Therefore, we utilized the Optuna [31] framework to seek the optimal hyperparameter values. Optuna is an automated hyperparameter optimization framework capable of pruning suboptimal experiments. It conducts multiple trials to find the best set of hyperparameter values, aiming to expedite and automate the hyperparameter optimization process. In Figs. S2-S4, we observe that the ARI converges within 100 trials. Specifically, the ARI values stabilize and remain high after ~ 40 to 50 trials. This indicates that using 100 trials is sufficient to achieve stable and reliable clustering results across different datasets. Therefore, we chose 100 trials as the standard to ensure consistency and reliability of the results. In this paper, we use 16 datasets [32–34] to train the two hyperparameters by using Optuna with 100 trials and validate the performance in the other 15 datasets [25, 35, 36] in the follow-up case studies (we present detailed information about datasets in Table S1 and Table S2). We ran 100 trials on every different dataset with Optuna and identified the most favorable loss rate and tailor rate values (we present detailed outcome in Table S3). And we selected a median position as the final hyperparameters value. Ultimately, we set the loss rate at 2 and the tailor rate at 0.9.

### Low-expression optimized DAE

The low-expression optimized DAE comprises two main components: an encoder and a decoder. The encoder accepts z-score data, denoted as $\overline{X}$, as input, and its output layer generates a dimensionality-reduced hidden embedding $H$. The decoder then maps $H$ to the reconstructed data of the original input $\overline{X}$. Both the encoder and decoder are neural networks based on multilayer perceptron (MLP). scRNA-seq data are characterized by high dimensionality, and the simple structure of a MLP can directly handle high-dimensional numerical data. By progressively learning gene expression features through multiple hidden layers, MLPs are well-suited to capture the nonlinear characteristics of the data. Additionally, scRNA-seq typically includes thousands of cells and tens of thousands of genes. The computational complexity of MLPs is relatively low, making them suitable for rapid iteration on large-scale data. In contrast, other neural networks such as Kolmogorov–Arnold Networks require higher computational resources and more complex training processes, especially when dealing with high-dimensional and sparse data. By directly processing the data, MLPs can effectively capture noise patterns within the data and perform denoising. Subsequently, three distinct fully connected layers are utilized to fit the mean ($\mu$), dispersion ($\theta$), and dropout probability ($\pi$), respectively, as follows:


$$ \kern-.6pc H={W}_{\mathrm{encoder}}\left(\overline{X}+\epsilon \right), $$

$$ \kern-1.8pc D={W}_{\mathrm{decoder}}(H), $$

$$ \kern-.6pc\pi =\mathrm{Sigmoid}\left(D{W}_{\pi}\right), $$

$$ \mu =\mathrm{Diag}\left[\exp \left(D{W}_{\mu}\right)\right], $$

$$ \kern-2.1pc\theta =\exp \left(D{W}_{\theta}\right), $$

where $\epsilon$ represents random Gaussian noise with a mean of 0 and a variance of 0.01, introduced to enhance the model’s denoising capabilities for scRNA-seq data; $\mu$ denotes the mean of gene expression; $\theta$ is the dispersion parameter, indicating the probability of a dropout event when the original count is zero, which is used to measure the variability of gene expression levels; and $\pi$ signifies the probability of the zero-inflation component. The three fully connected layers are activated by different nonlinear functions to increase expressiveness. Since the dropout probability ($\pi$) lies between zero and one, a sigmoid function is employed for standardization. The mean ($\mu$) and dispersion ($\theta$), as non-negative values, are activated using the exponential function. To prevent overfitting, the mean ($\mu$) is normalized to match the original counts for each cell. And all $W$ represent learnable parameters within the model. Ultimately, the loss function is defined as the sum of the negative log-likelihoods of the ZINB distribution, which is a critical metric for measuring model performance. Moreover, the low-expression optimized DAE employs the aforementioned changed ZINB loss to enhance the focus on genes with lower expression levels, utilizing ${X}^{\prime }$ instead of $X$ in the loss calculation, as follows:


$$ {P}_{NB}\left({X}^{\prime }|\mu, \theta \right)=\frac{\varGamma \left({X}^{\prime }+\theta \right)}{\varGamma \left({X}^{\prime }+1\right)\varGamma \left(\theta \right)}\times{\left(\frac{\theta }{\theta +\mu}\right)}^{\theta}\times{\left(\frac{\mu }{\theta +\mu}\right)}^{X^{\prime}}, $$

$$ {P}_{ZINB}\left({X}^{\prime }|\pi, \mu, \theta \right)=\pi \delta \left({X}^{\prime}\right)+\left(1-\pi \right)\times{P}_{NB}\left({X}^{\prime }|\mu, \theta \right), $$

$$ {L}_{\mathrm{ZINB}}=-\mathit{\log}\left({P}_{\mathrm{ZINB}}\left({X}^{\prime }|\pi, \mu, \theta \right)\right) , $$

### GCN-based GAE

In this study, we employed a GAE to capture the topological information between cells. This approach allows for the learning of low-dimensional embedding representations of nodes from the graph structure and the reconstruction of the graph’s adjacency matrix. The GAE model consists of an encoder and a decoder. In our work, as opposed to the classical GAE encoder, which uses GNN layers to generate node embeddings, our encoder unit utilizes a GCN [37]. Given that scRNA-seq data typically contain a significant amount of noise and sparsity, the GCN is able to more effectively capture and integrate the complex interaction information between cells, by leveraging the spatial relationships inherent in graph-structured data. The formula for each GCN layer is as follows:


$$ {Z}^{(l)}=\tanh \left(\hat{A}{Z}^{\left(l-1\right)}{W}^{\left(l-1\right)}\right), $$

$$ \tanh (x)=\frac{e^x-{e}^{-x}}{e^x+{e}^{-x}}, $$

where $\hat{A}$ represents the symmetrically normalized adjacency matrix $A$ with added self-loops. The matrix ${Z}^{(l)}$ denotes the feature matrix of nodes at the ${l}^{th}$ layer, while ${W}^{\left(l-1\right)}$ is the trainable weight matrix for the ${\left(l-1\right)}^{th}$ layer. The activation function used is the hyperbolic tangent ($\tanh$), which is a widely used activation function in neural network architectures. Our decoder employs a dot product reconstruction method, which reconstructs the adjacency matrix by calculating the dot product between node embeddings. The formula for our decoder is as follows:


$$ \tilde{A}=\sigma \left({Z}^l{\left({Z}^l\right)}^T\right), $$

where the reconstructed adjacency matrix is denoted as $\tilde{A}$, with dimensions of $N\times N$, and the activation function $\sigma$ employed is the Sigmoid function. The loss function for the GAE is defined by the mean squared error between the reconstructed and the original graph, formulated as follows:


$$ L=\frac{1}{N^2}\sum_{i,j=1}^N{\left({A}_{ij}-{\tilde{A}}_{ij}\right)}^2, $$

Within the framework, the elements of the original graph and the reconstructed graph at the $ {i}^{th}$, row and $ {j}^{th}$ column are represented by $ {A}_{ij} $ and $ {\tilde{A}}_{ij} $ respectively.

### Multiple attention mechanism

The multi-head attention mechanism is an extension of the attention mechanism that allows the model to simultaneously focus on information across different representation subspaces. Compared to the traditional attention mechanism, the multi-head attention mechanism enables the model to learn different features of the data from multiple perspectives, thereby enhancing the model’s overall capacity to comprehend information. To integrate the respective advantages of low-expression optimized DAE and GAE, and to mitigate the deficiencies in the features extracted by each, the multi-head attention mechanism is utilized to combine the denoised data embedding with the topological structure embedding, as follows:


$$ {Q}_M^l={W}_M^q{Z}^l,{K}_M^l={W}_M^K{E}^l,{V}_M^l={W}_M^V{E}^l , $$

$$ Attention\ score:{a}_M^l=\mathrm{softmax}\left({Q}_M^l\cdotp{K}_M^l\right) , $$

$$ Output:{R}^l=W\cdotp Concat\left({a}_1^l\cdotp{V}_1^l,{a}_2^l\cdotp{V}_2^l,\dots, {a}_M^l\cdotp{V}_M^l\right)+{E}^l, $$

where the query vector ${Q}_M^l$ is derived from the topological embedding ${Z}^l$ generated by the GAE. Concurrently, the key ${K}_M^l$ and value ${V}_M^l$ are produced from the denoising embedding ${E}^l$ by the low-expression optimized DAE. The attention score is computed using a dot-product attention schema. $M$ signifies the count of attention heads. All instances of $W$ and ${W}_M$ are indicative of trainable parameters. Within the scLEGA architecture, dual information fusion modules are employed, thus yielding two sequential outputs ${R}^l$ based on the ${l}^{th}$ embeddings from both GAE and low-expression optimized DAE. The resultant ${R}^l$ serves as the input for the subsequent encoding layer, which consists of fully connected layers followed by ReLU activation, and ${R}^2$ is utilized as the feature representation for the clustering phase.

### Unsupervised deep embedding clustering

We employed an unsupervised algorithm to refine the clustering until convergence. In the first phase of the algorithm, we assessed the similarity between data points in the embedded space by calculating the soft membership of each data point to the cluster centers, utilizing the Student’s t-distribution as the kernel function. Here, ${z}_i$ denotes the embedded point for the cell $i$, and the centroid ${\mu}_j$ represents cluster $j$ (where $j$ = 1, 2, …, $k$), obtained from the Leiden algorithm with the pre-trained embedding $Z$ as input and if we know the number of categories $k$, although the Leiden algorithm does not require a predetermined number of clusters, knowing the value of k in advance can be advantageous during parameter selection and algorithm tuning. For example, the known k can be used to set reasonable initial conditions or optimization criteria, potentially improving the quality of the clustering results. Additionally, the known k can serve as a benchmark to validate the clustering results of the Leiden algorithm. By comparing the actual number of clusters obtained with the known k, the accuracy and effectiveness of the algorithm can be assessed, leading to further optimization and improvement of the algorithm. And the kernel function as follows:


$$ {q}_{ij}=\frac{{\left(1+{\left\Vert{z}_i-{\mu}_j\right\Vert}^2/\alpha \Big)\right)}^{-1}}{\sum_{k=0}^k{\left(1+{\left\Vert{z}_i-{\mu}_k\right\Vert}^2/\alpha \right)}^{-1}} , $$

where the degree of freedom for the student’s t-distribution is denoted by $\alpha$. We usually [29, 38] set $\alpha$ as one. In the second step, we refine the clustering by learning the cells with high-confidence cluster assignments, aided by the optimized distribution of high confidence. The distribution is defined as follows:


$$ {p}_{ij}=\frac{q_{ij}^2/{\sum}_{i=1}^n{q}_{ij}}{\sum_{j=1}^K\left({q}_{ij}^2/\sum_{i=1}^n{q}_{ij}\right)\ } $$

The optimization process of clustering is defined by the Kullback–Leibler divergence loss between the soft cell assignments ${q}_i$ and the auxiliary distribution ${p}_i$ for the cell $i$, as follows:


$$ {L}_{kl- loss}= KL\left(P\Vert Q\right)={\sum}_{i=1}^n{\sum}_{j=1}^K{p}_{ij}\mathit{\log}\left(\frac{p_{ij}}{q_{ij}}\right) , $$

In summary, the loss function of the model is defined as follows:


$$ train:{L}_{train}={L}_{zinb}+{r}_1{L}_{graph} , $$

$$ fine\ tune:L={L}_{kl- loss}+{r}_2{L}_{train} , $$

The scLEGA was developed within a Python 3.8 environment, featuring a hidden layer architecture with dimensions sequentially set at 256, 64, 16, 64, and 256. The model incorporates two multi-head attention blocks, each comprising eight heads. To enhance training efficiency, the low-expression optimized DAE and GAE share a common dimensionality reduction linear layer. The optimizer of choice was Adam, with a learning rate set at 0.001 and a gradient clipping strategy employing a maximum L2 norm of three to prevent overfitting. During the fine-tuning phase, clustering operations are exclusively performed, and training is prematurely terminated if label changes are less than 1/1000 of the total count. Additionally, ${r}_1$ and ${r}_2$ are parameters utilized to balance multi-objective optimization, with default values set at 0.1.

### Evaluation metrics

To evaluate the effectiveness of clustering methods for scRNA-seq datasets, we utilize datasets with cell labels denoted as $L=\left\{{L}_1,{L}_2\dots, {L}_n\right\}$. We define the predicted labels as $L=\left\{{L}_1^{\prime },{L}_2^{\prime },\kern0.5em \dots, {L}_3^{\prime}\right\}$, using the labels provided by these datasets as the ground truth. The model’s performance is assessed using the adjusted Rand index (ARI) [39] and normalized mutual information (NMI) [40] as metrics. These metrics compare the similarity between the predicted labels and the true values. Higher values indicate more accurate clustering results.

NMI is defined within the range [0,1], representing a combination of mutual information (MI) and entropy. It addresses the issue of MI’s sensitivity to the number of clusters. The computation of NMI is as follows:


$$ \mathrm{NMI}=\frac{\mathrm{MI}\left({L}^{\prime },L\right)}{\sqrt{H\left({L}^{\prime}\right)H(L)}} $$

The term $\frac{\mathrm{MI}\left({L}^{\prime },L\right)}{\sqrt{H\left({L}^{\prime}\right)H(L)}}$ is utilized to obtain the MI between $L$ and ${L}^{\prime }$, where $H\left({L}^{\prime}\right)$ and $H(L)$ denote the entropy of the clustering results and the true classification, respectively. The ARI accounts for the number of sample pairs that are assigned to the same or different clusters in the true classification and the clustering results. The range of ARI is [−1,1]. The calculation method for ARI is as follows:


$$ \mathrm{ARI}=\frac{\sum_{ij}\left(\begin{array}{@{}c@{}}{n}_{ij}\\{}2\end{array}\right)-\left[{\sum}_i\left(\begin{array}{@{}c@{}}{a}_i\\{}2\end{array}\right){\sum}_j\left(\begin{array}{@{}c@{}}{b}_j\\{}2\end{array}\right)\right]\Big/\left(\begin{array}{@{}c@{}}n\\{}2\end{array}\right)}{\frac{1}{2}\left[{\sum}_i\left(\begin{array}{@{}c@{}}{a}_i\\{}2\end{array}\right)+{\sum}_j\left(\begin{array}{@{}c@{}}{b}_j\\{}2\end{array}\right)\right]-\left[{\sum}_i\left(\begin{array}{@{}c@{}}{a}_i\\{}2\end{array}\right){\sum}_j\left(\begin{array}{@{}c@{}}{b}_j\\{}2\end{array}\right)\right]\Big/\left(\begin{array}{@{}c@{}}n\\{}2\end{array}\right)} $$

Here, ${n}_{ij}$ represents the number of cells that belong to the category $i$ in the true classification and cluster $j$ in the clustering results; ${a}_i$ denotes the number of cells within category $i$ of the true classification, while ${b}_j$ refers to the number of cells within cluster $j$ of the clustering results.

## Results

### Comparison of clustering results with other methods

To evaluate the clustering performance of scLEGA, we ran scLEGA on 15 real scRNA-seq datasets from various organs or platforms, obtaining clustering results. These were compared with 12 single-cell clustering methods using default parameters: DeepScena, scGPCL, scBGEDA, scGNN, graph-sc [41], DESC, Leiden (SCANPY), SC3, scGAC, scDeepCluster, scvi-tools [42], and SCEA [43] as benchmark methods. We assessed each clustering model with two widely recognized clustering metrics (NMI and ARI) to demonstrate the performance of our model. All comparative methods were employed with the authors’ recommended default parameters or settings. The input data for each clustering method underwent the same preprocessing, and experiments were repeated five times on each dataset.

**Figure 2:**
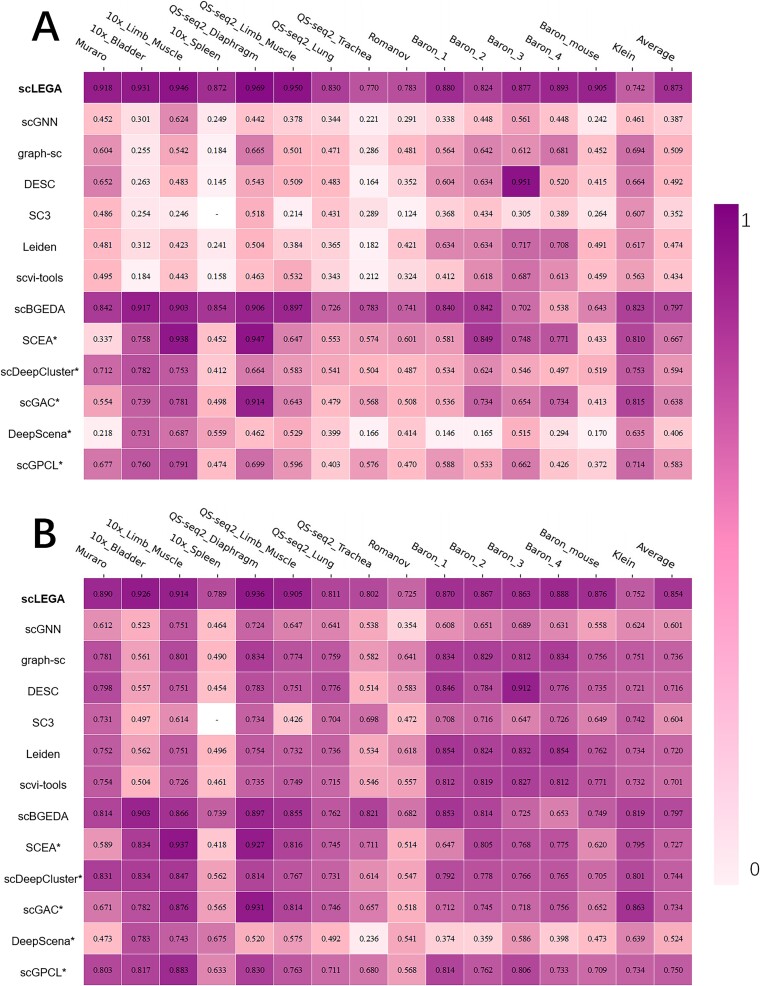
Clustering performance of scLEGA and twelve other clustering methods. Methods that require the specification of the number of clusters are marked with an asterisk (*). (A) ARI value, (B) NMI value.

Figure 2 A and B illustrate the impact of 13 clustering methods on fifteen scRNA-seq datasets. The white areas in the figure indicate the absence of clustering results due to time or space complexity constraints. It is evident from the figure that the overall results of scLEGA on the 15 datasets are better than the other 12 compared methods. The average ARI score improved by ~ 9.5%–147.9%, and the average NMI score increased by 7.2%–42.1%. Specifically, scLEGA achieved the best NMI and ARI values in 11 datasets. Although the metric values for Baron Pancreas 3, Klein, and Quake 10x Limb Muscle datasets were not the highest, they were similar to the highest values and superior to most methods. Notably, scLEGA’s performance surpassed that of community detection-based methods (scGNN, graph-sc, DESC, Leiden, scvi-tools). Like these methods, scLEGA employed the same strategy of avoiding manually setting the predicted number of clusters, resulting in exceptional outcomes. Moreover, even for methods that rely on a known number of cell types, scLEGA’s overall performance still exceeded that of k-means-based methods. In general, scLEGA demonstrated competitive performance in the inference of cell types.

### Validity of latent characteristics

**Figure 3:**
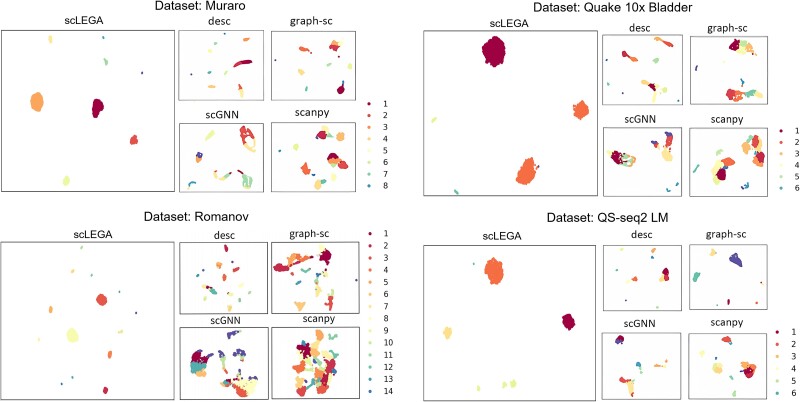
Comparison of UMAP visualization from four other deep models. (A) Romanov dataset, (B) Quake_10x_Bladder dataset, (C) Quake_Smart-seq2_Limb_Muscle dataset, and (D) Muraro dataset.

**Figure 4:**
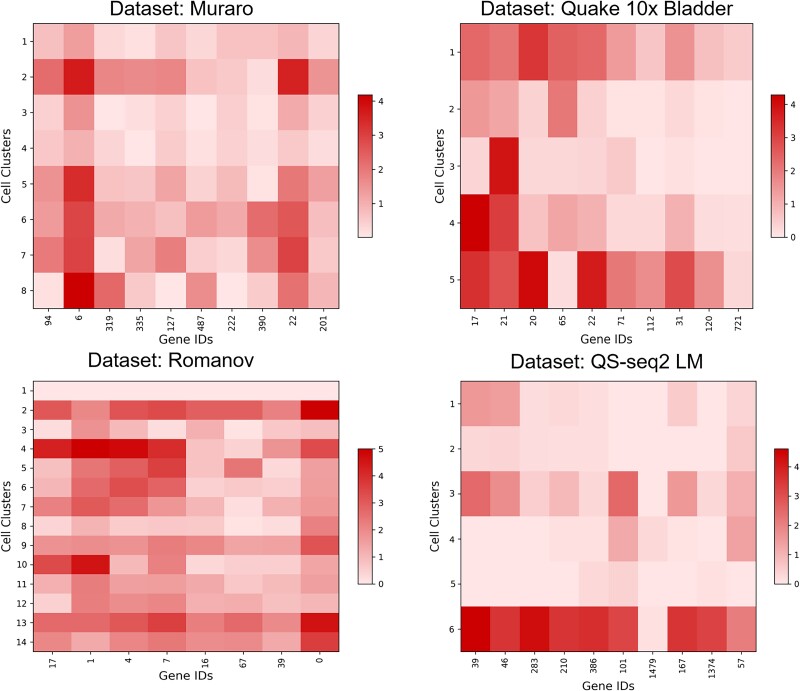
Expression heat plot of identified DEGs in different cell clusters on four datasets: Romanov, Quake 10x Bladder, Quake Smart-seq2 Limb Muscle, and Muraro.

To validate the efficacy of scLEGA’s potential features and to analyze the quality of cellular representation in clustering, we visualized the latent features obtained by scLEGA and four other community detection-based methods using UMAP on four datasets of varying sizes, as follows (the results for the other 11 datasets are displayed in Fig. S6–S20): Romanov (2881 cells), Quake_10x_Bladder (2500 cells), Quake_Smart-seq2_Limb_Muscle (1090 cells), and Muraro (2122 cells). As illustrated in Fig. 3, different colors in the graph represent different cell types. It is intuitively evident that, compared to the four other community detection-based methods, scLEGA performs the best in distinguishing real cell clusters and in making the boundaries between clusters more distinct. In contrast, other methods tend to mix different cell types. This indicates that scLEGA can learn useful information from actual datasets while reducing the impact of noise on the data. In order to better demonstrate the clustering ability of scLEGA, we also carried out differential expression analysis on the datasets and showed the results in Fig. 4, which illustrate scLEGA can effectively distinguish the DEGs for clustering.

**Figure 5:**
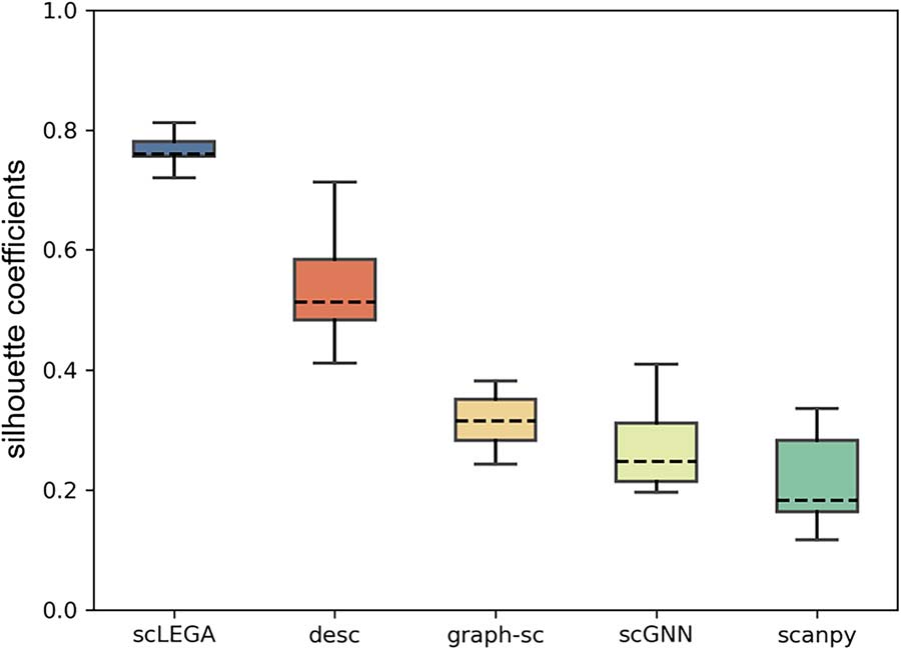
The silhouette coefficients obtained by scLEGA and four clustering methods based on community detection algorithms. Each box contains the results of 15 datasets (repeated experiments five times on each dataset).

To more clearly demonstrate scLEGA’s capability to differentiate unknown cells, we employed a common internal clustering evaluation: the Silhouette coefficient, to assess clustering performance. The Silhouette coefficient [44], which combines cohesion and separation, ranges from [−1, 1], with values closer to 1 indicating better clustering and values closer to −1 indicating poorer clustering. We conducted experimental comparisons using the latent features and predicted results of unknown cells obtained by the deep model. As shown in Fig. 5, compared to the other four community detection-based methods, scLEGA achieved the optimal clustering performance, signifying its eminent ability to identify unknown cells. Among the baseline methods, both graph-sc and scGNN utilized GAE for feature extraction, but due to treating representation learning and clustering as separate tasks, they achieved suboptimal performance. In contrast, DESC, which adopts a soft clustering strategy to simultaneously optimize feature extraction and clustering assignment, obtained a higher silhouette score. However, scLEGA not only considered the aforementioned methods, employing a multi-head attention mechanism combined with denoising and topological embedding to construct cellular representations, but it also took into account the contribution of genes with relatively low expression levels to cell type inference, thereby achieving the best performance.

### Adaptive selection of cluster centers

scLEGA employs the Leiden algorithm to identify and establish initial cluster centers. By analyzing the distribution of cells in a latent space, the model adaptively assigns labels to each cell, implementing a soft partitioning strategy that does not rely on a predetermined number of groups. This process, independent of the prior knowledge of the subpopulation count, provides robust support for exploring unknown or complex biological systems. Furthermore, scLEGA integrates denoising and topological embedding based on a multi-head attention mechanism, resulting in closer cell representations of similar cells in the latent embedding, thereby distinguishing different cellular states.

**Figure 6:**
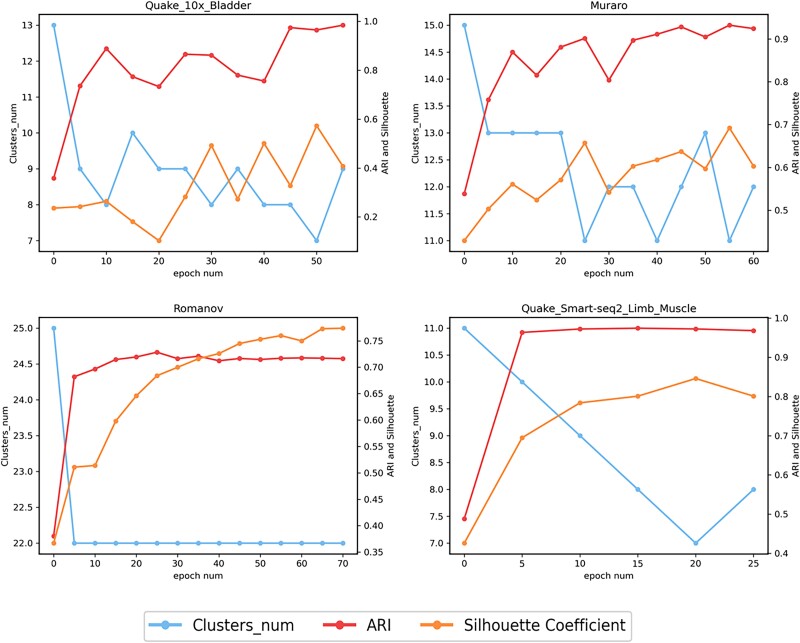
In the clustering stage, the cluster center adaptively adjusts with the training epochs, and the change in ARI and Silhouette coefficient with the training epochs.

During each iteration of the clustering phase, cells can evaluate their similarity to others through the attention mechanism, continuously adjusting the number and position of cluster centers to select the most appropriate ones, enhancing the flexibility and adaptability of clustering. Interestingly, as observed in Fig. 6 (the results for the other 11 datasets are displayed in Fig. S21–S35), the Leiden algorithm initially tends to set a larger number of cluster centers. Although this strategy may seem overly granular at the start, it provides the model with greater flexibility to explore the underlying structure of the data. As the model training progresses, some initially set cluster centers are deemed redundant, and the model gradually prunes these, removing unreasonable or unstable categories. This adaptive pruning process not only reduces the impact of noise but also ensures that the remaining cluster centers more accurately represent the true patterns in the data. Ultimately, by dynamically adjusting the quantity and location of cluster centers, the model effectively enhances the overall performance of clustering, ensuring the accuracy and reliability of the clustering results.

### Model stability

**Figure 7:**
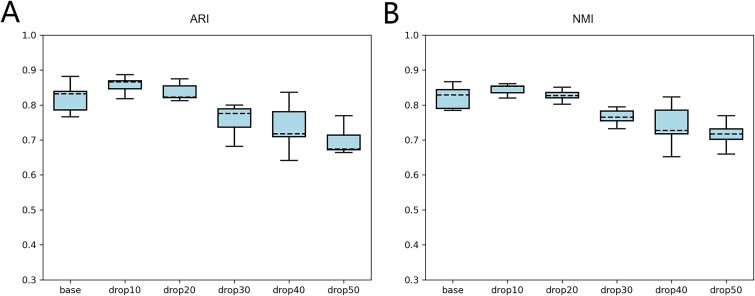
Clustering performance of scLEGA on eight scRNA-seq datasets (repeated experiments five times on each dataset) with 10%, 20%, 30%, 40%, and 50% dropout rates. The base is the performance of scLEGA without manual dropout. (A) ARI value, (B) NMI value.

Given the characteristic sparsity of scRNA-seq data, to validate the model’s stability with sparse datasets, we employed a manual dropout approach. We conducted five experiments across eight scRNA-seq replicates, utilizing the average ARI and NMI to assess clustering performance, thereby examining the stability and robustness of scLEGA. We randomly set 10%–50% of gene expression values to zero within the actual scRNA-seq dataset. As illustrated in Fig. 7, intriguingly, we observed that manual dropout rates of 10%–20% yielded better results than the baseline scenario, with no significant decline at 30%–50% dropout rates. This indicates that our model can effectively extract features from sparse data for cell type inference, demonstrating robustness and stability with sparse scRNA-seq data. It is well-known that dropout is considered a form of noise, and the denoising capability of the DAE played a role in mitigating this noise. Moreover, in scenarios with more missing values, genes with lower expression levels may have a greater impact. Since scLEGA thoroughly considers the contribution of genes with relatively lower expression to cell type classification, the results at lower manual dropout rates showed an improvement over the baseline model.

### Ablation study

**Figure 8:**
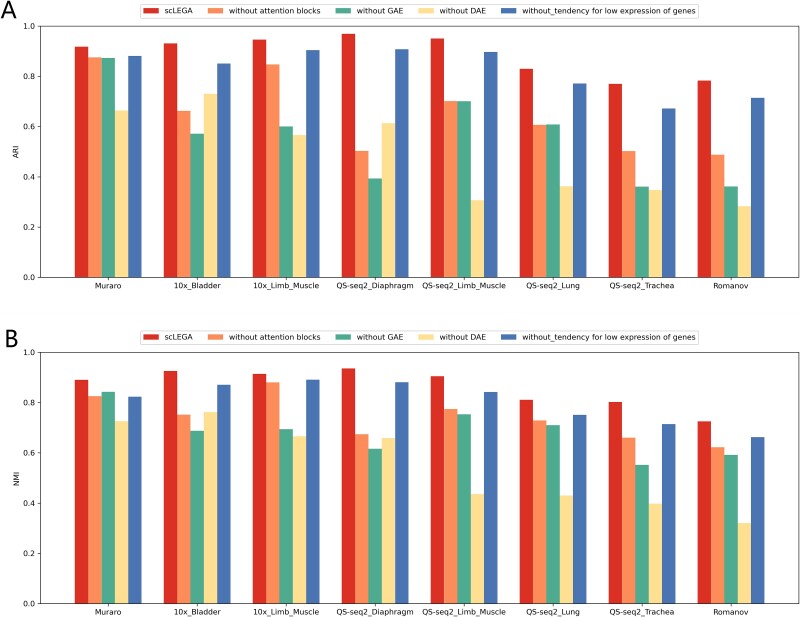
The scLEGA model underwent ablation experiments on eight scRNA-seq datasets. The experiments tested the average ARI and NMI values under four scenarios involving the removal of various components, including the absence of a multi-head attention mechanism module (without attention block), the absence of a GCN-based GAE (without GAE), the absence of a low-expression optimized DAE (without DAE), and not utilizing the modified ZINB loss (without tendency for low expression of genes). (A) ARI value, (B) NMI value.

To evaluate the importance of individual components within scLEGA, we removed various elements and assessed the performance of scLEGA without the multi-head attention mechanism (without attention block), without the GCN-based GAE (without GAE), without the low-expression optimized DAE (without DAE), and without the modified ZINB loss (without tendency for low expression of genes). The experimental results displayed the average ARI and NMI values across eight datasets. As illustrated in Fig. 8, scLEGA’s clustering performance was superior, and a performance decline was observed when the attention layer was replaced by a multi-layer perceptron. The removal of the low-expression optimized DAE, which diminished the denoising capability for scRNA-seq data, also led to a performance decrease. Moreover, this removal equated to the absence of the modified ZINB loss as well, resulting in a more significant decline in some datasets. Additionally, tests were conducted without the GCN-based GAE, where the keys and values for the attention layer were sourced from embeddings generated by the DAE. The findings indicated that the lack of topological structure information in the data precipitated a reduction in model performance. Lastly, substituting the modified ZINB loss with the standard ZINB loss still resulted in a performance downturn, confirming that scLEGA indeed capitalizes on the low-expression optimized DAE to adequately consider the contribution of genes with relatively low expression levels to cell type classification, thereby improving the precision of cell type identification.

## Discussion

In this work, we introduce a cell type inference method based on the scLEGA deep model, which employs a novel ZINB loss function that fully considers the contribution of genes with relatively low expression levels to cell type classification. The scLEGA model integrates two distinct scRNA-seq clustering strategies through a multi-head attention mechanism. It utilizes a DAE based on the ZINB model to extract low-dimensional features and address dropout events, while the GAE leverages neighbor information to guide dimensionality reduction. The iterative fusion of denoising and topological embedding in scLEGA facilitates the acquisition of cluster-friendly cell representations in the hidden embedding, where similar cells are brought closer together. Our experimental results demonstrate that the proposed scLEGA framework effectively achieves clustering, dimensionality reduction, and visualization of single-cell data, exhibiting robust denoising capabilities, as well as stability and robustness. Moreover, genes with lower expression levels are adequately considered, enabling the model to learn more cluster-friendly representations. Additionally, scLEGA utilizes the Leiden algorithm to determine initial cluster centers, obviating the need for prior knowledge of group numbers. While scLEGA accounts for cell topological embeddings obtained from the GCN-based GAE and potential features of low-expression genes from the low-expression optimized DAE, the multi-head attention mechanismrequires computation and allocation of different attentions to central and neighboring nodes during clustering, which increases computational time and resource consumption. Despite our framework’s adept handling of the sparsity characteristic of scRNA-seq data, there remains room for improvement to further address this feature. In the future, to better analyze different scRNA-seq datasets, we plan to embed an imputation mechanism within scLEGA to more effectively clean the raw data and reduce noise.

Overall, scRNA-seq technology can be utilized to reveal heterogeneity among cells, identify new cell types, trace developmental pathways, and understand cellular behavioral changes under disease conditions. In biomedical research, scRNA-seq has been employed to study various complex biological processes, including the tumor microenvironment, neuroscience, developmental biology, and diabetes. However, these downstream analyses are predicated on the ability to infer cell types from scRNA-seq data, prompting the development of many advanced deep learning models for this purpose. In the future, deep learning frameworks, including scLEGA, will be further researched to enhance performance while reducing resource and time consumption, thereby successfully leveraging scRNA-seq data for the benefit of humanity.

**Key Points**

- We develop an attention-based deep clustering model with a tendency for low expression of genes called the scLEGA. It solves the problem that the existing deep models often ignore the role of low expression genes in clustering, which makes these models cannot be widely used in various datasets.
- scLEGA modified the ZINB loss of DAE by introducing two hyperparameters to adjust the proportion and tendency of low-expressed genes, to better obtain the potential characteristics of the scRNA-seq data. What’s more, by employing a multi-head attention mechanism, the features derived from DAE and GAE are integrated, mapping the scRNA-seq data to a more suitable feature space, and an unsupervised algorithm to refine the clustering.
- scLEGA does not need to know the number of clusters in advance, expanding its scope of application. The robustness and efficiency of scLEGA are proved by various experiments.

## Supplementary Material

Supplementary_bbae371

bbae371

## Data Availability

The implementation of scLEGA is available at: https://github.com/Masonze/scLEGA-main. The data that support the findings of this study are openly available at https://github.com/Masonze/scLEGA-main/tree/master/Data. scLEGA is implemented in Python 3 (version 3.8) using PyTorch (version 1.10.0 + cu113). All experiments are conducted on an NVIDIA 3080ti GPU with 12 GB of memory.
