# Supplementary material for: scLEGA: an attention-based deep clustering method with a tendency for low expression of genes on single-cell RNA-seq data
Source: Brief Bioinform. 2024 Jul 26;25(5):bbae371. doi: 10.1093/bib/bbae371 (PMC11281828; doi:10.1093/bib/bbae371)
Supplement: Supplementary_bbae371 [file supplementary_bbae371.docx]

**Table S1**: Summary of the real scRNA-seq datasets for training the two hyper-parameters.

| **Index** | **Dataset Name** | **Number of Cell** | **Cell groups** | **Genes** | **Cell source** | **Platform** |
| --- | --- | --- | --- | --- | --- | --- |
| 1 | Chen | 12089 | 46 | 16383 | mouse | Drop-seq |
| 2 | Mouse | 2100 | 16 | 20670 | mouse | Atlas project |
| 3 | Petropoulos | 1529 | 5 | 21749 | Human | ArrayExpress |
| 4 | YAN | 90 | 7 | 16383 | Human | Tang |
| 5 | Zeisel | 3005 | 9 | 16383 | Mouse | STRT-Seq |
| 6 | 10X_PBMC | 4271 | 8 | 16653 | Human | 10 X |
| 7 | Mouse bladder cell | 2746 | 16 | 20670 | Mouse | Microwell-seq |
| 8 | Mouse ES cells | 2717 | 4 | 20670 | Mouse | Droplet barcoding |
| 9 | Worm neuron cell | 4186 | 10 | 13488 | Worm | scRNA-seq |
| 10 | PBMC_68k | 68579 | 10 | 20387 | Human | 10 X |
| 11 | Shekhar_mouse_retina | 27499 | 19 | 13166 | mouse | scRNA-seq |
| 12 | kolo | 704 | 3 | 38653 | Mouse | unknown |
| 13 | Lawlor | 638 | 8 | 26616 | Human | unknown |
| 14 | Muraro | 3072 | 11 | 19059 | Human | CEL-seq2 |
| 15 | Baron_human | 8569 | 14 | 17499 | Human | inDrop |
| 16 | quake_10x_heart_and_aorta | 624 | 5 | 16383 | Mouse | 10 X Genomics |

**Table S2**: Summary of the real scRNA-seq datasets for validate our model.

| **Index** | **Dataset Name** | **Number of Cell** | **Cell groups** | **Genes** | **Cell source** | **Platform** |
| --- | --- | --- | --- | --- | --- | --- |
| 17 | Klein | 2717 | 4 | 24047 | Mouse | Illumina HiSeq |
| 18 | Baron Pancreas 1 | 1937 | 14 | 16016 | Human | inDrop |
| 19 | Baron Pancreas 2 | 1724 | 14 | 16310 | Human | inDrop |
| 20 | Baron Pancreas 3 | 3605 | 14 | 16678 | Human | inDrop |
| 21 | Baron Pancreas 4 | 1303 | 14 | 15720 | Human | inDrop |
| 22 | Baron Pancreas mouse | 1886 | 13 | 14861 | Mouse | inDrop |
| 23 | Muraro | 2122 | 9 | 18819 | Human | CEL-seq2 |
| 24 | Quake 10x Bladder | 2500 | 4 | 16867 | Mouse | 10 X Genomics |
| 25 | Quake 10x Spleen | 9552 | 5 | 15621 | Mouse | 10 X Genomics |
| 26 | Quake 10x Limb Muscle | 3909 | 6 | 16512 | Mouse | 10 X Genomics |
| 27 | Quake Smart-seq2 Diaphragm | 870 | 5 | 17973 | Mouse | Smart-seq2 |
| 28 | Quake Smart-seq2 Trachea | 1350 | 4 | 19992 | Mouse | Smart-seq2 |
| 29 | Quake Smart-seq2 Lung | 1676 | 11 | 19390 | Mouse | Smart-seq2 |
| 30 | Quake Smart-seq2 Limb Muscle | 1090 | 6 | 18320 | Mouse | Smart-seq2 |
| 31 | Romanov | 2881 | 7 | 21143 | Human | Illumina HiSeq |

**Table S3**: Summary of the optimal value of two hyper-parameters for 16 datasets

| **Index** | **Dataset Name** | **Loss rate** | **Tailor rate** |
| --- | --- | --- | --- |
| 1 | Chen | 8.95 | 0.644 |
| 2 | Mouse | 1.65 | 0.601 |
| 3 | Petropoulos | 7.69 | 0.883 |
| 4 | YAN | 2.38 | 0.596 |
| 5 | Zeisel | 1.73 | 0.981 |
| 6 | 10X_PBMC | 1.42 | 0.670 |
| 7 | Mouse bladder cell | 1.48 | 0.506 |
| 8 | Mouse ES cells | 5.50 | 0.975 |
| 9 | Worm neuron cell | 1.93 | 0.990 |
| 10 | PBMC_68k | 8.24 | 0.999 |
| 11 | Shekhar_mouse_retina | 1.34 | 0.650 |
| 12 | kolo | 2.06 | 0.912 |
| 13 | Lawlor | 3.36 | 0.932 |
| 14 | Muraro | 3.34 | 0.985 |
| 15 | Baron_human | 1.82 | 0.665 |
| 16 | quake_10x_heart_and_aorta | 1.57 | 0.997 |


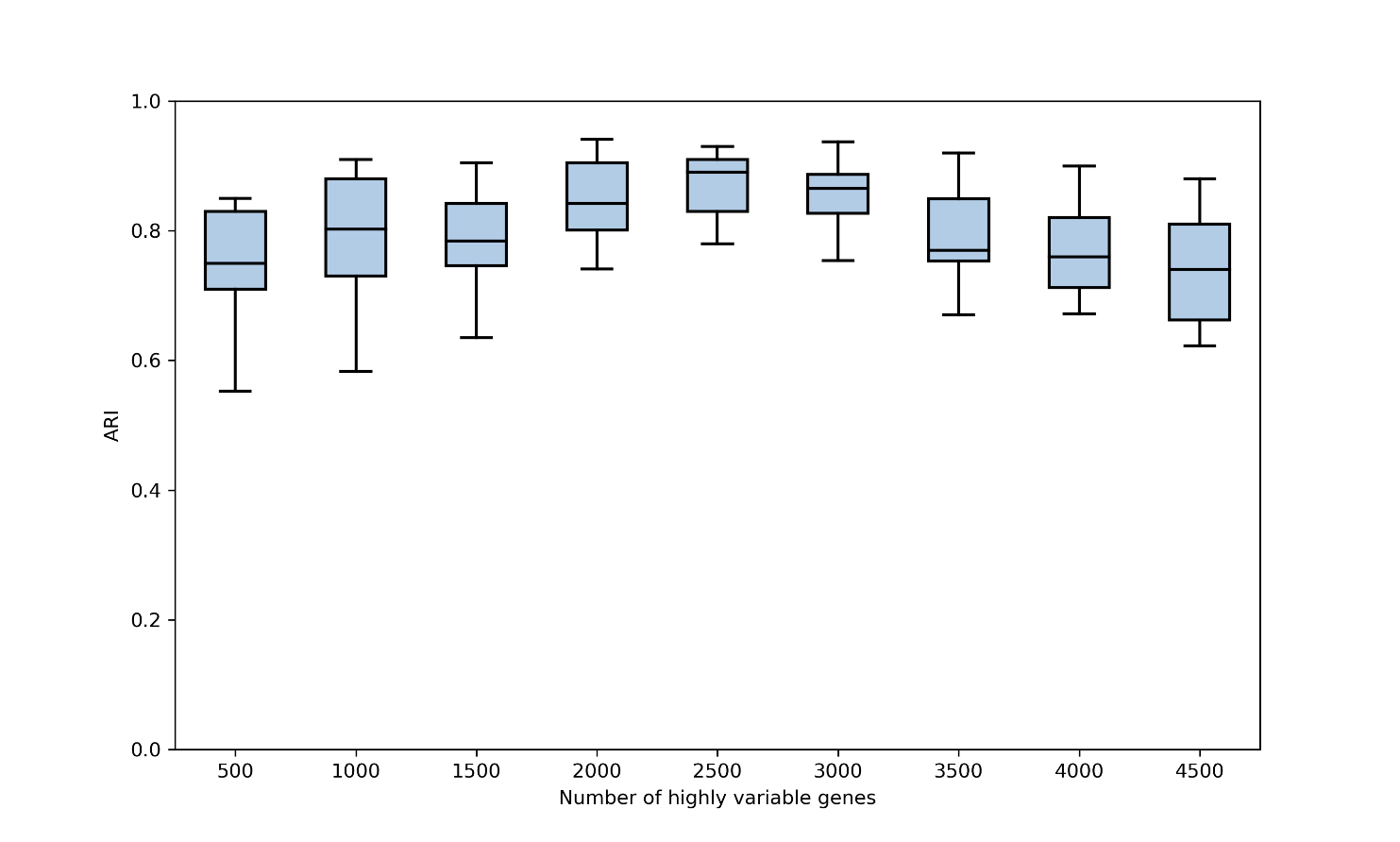


**Figure S1**: Comparison of different configurations for the number of highly variable genes.


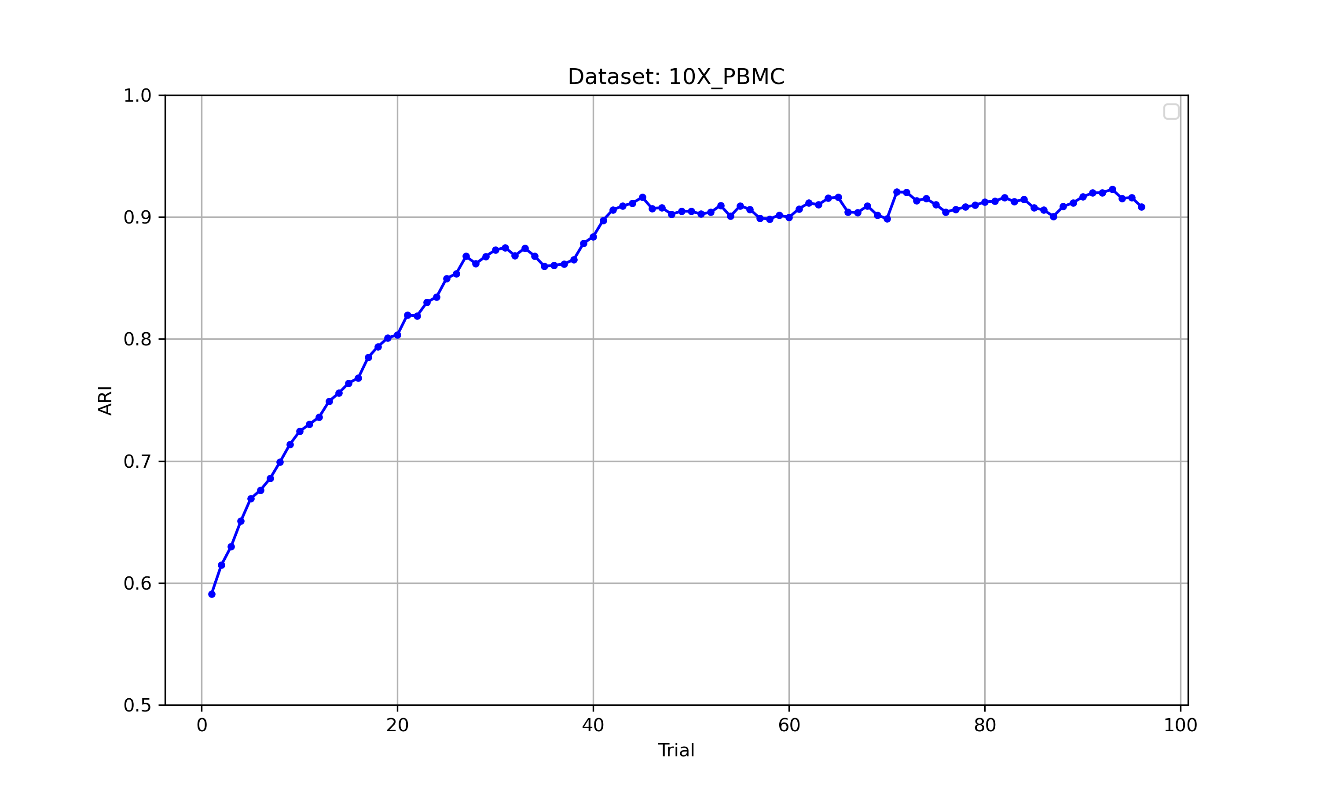


**Figure S2:** The optimization history for 10X_PBMC using Optuna.


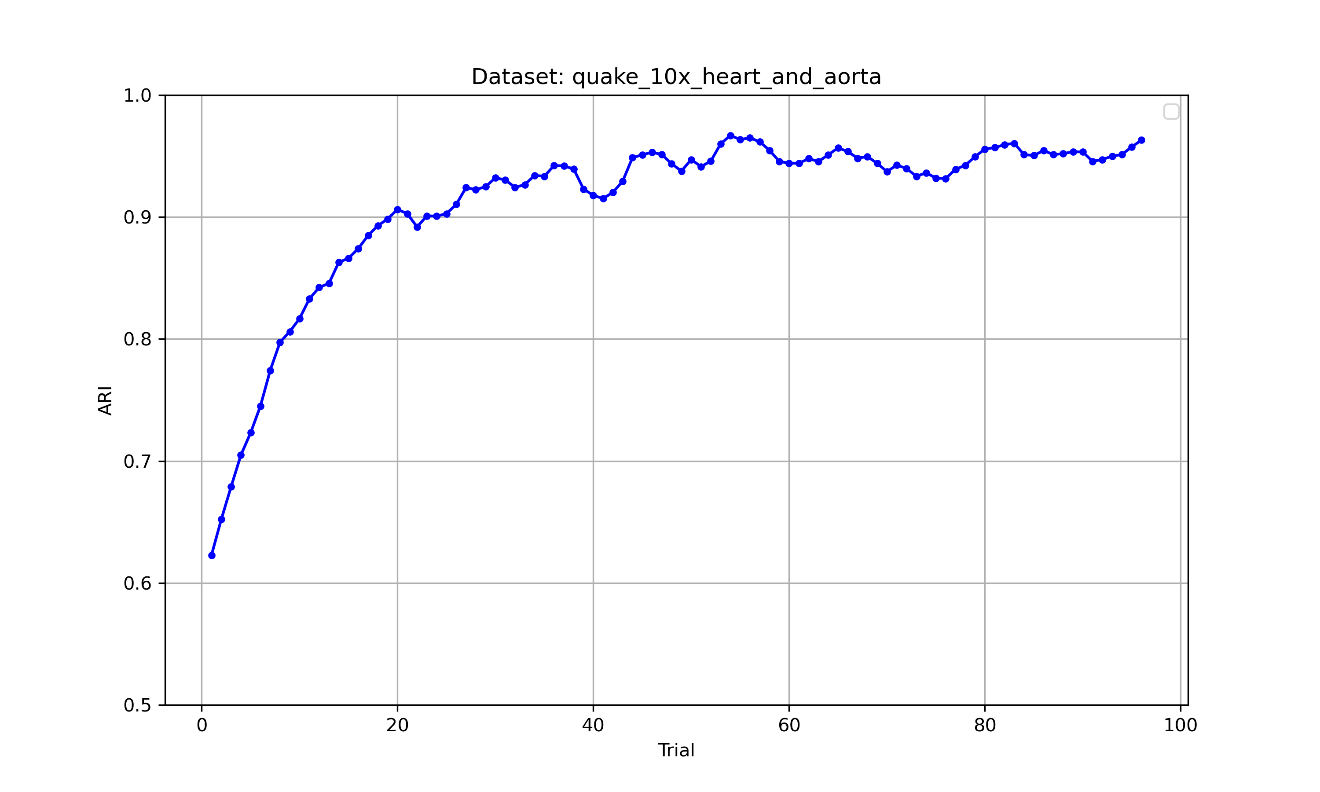


**Figure S3:** The optimization history for quake_10x_heart_and_aorta using Optuna.


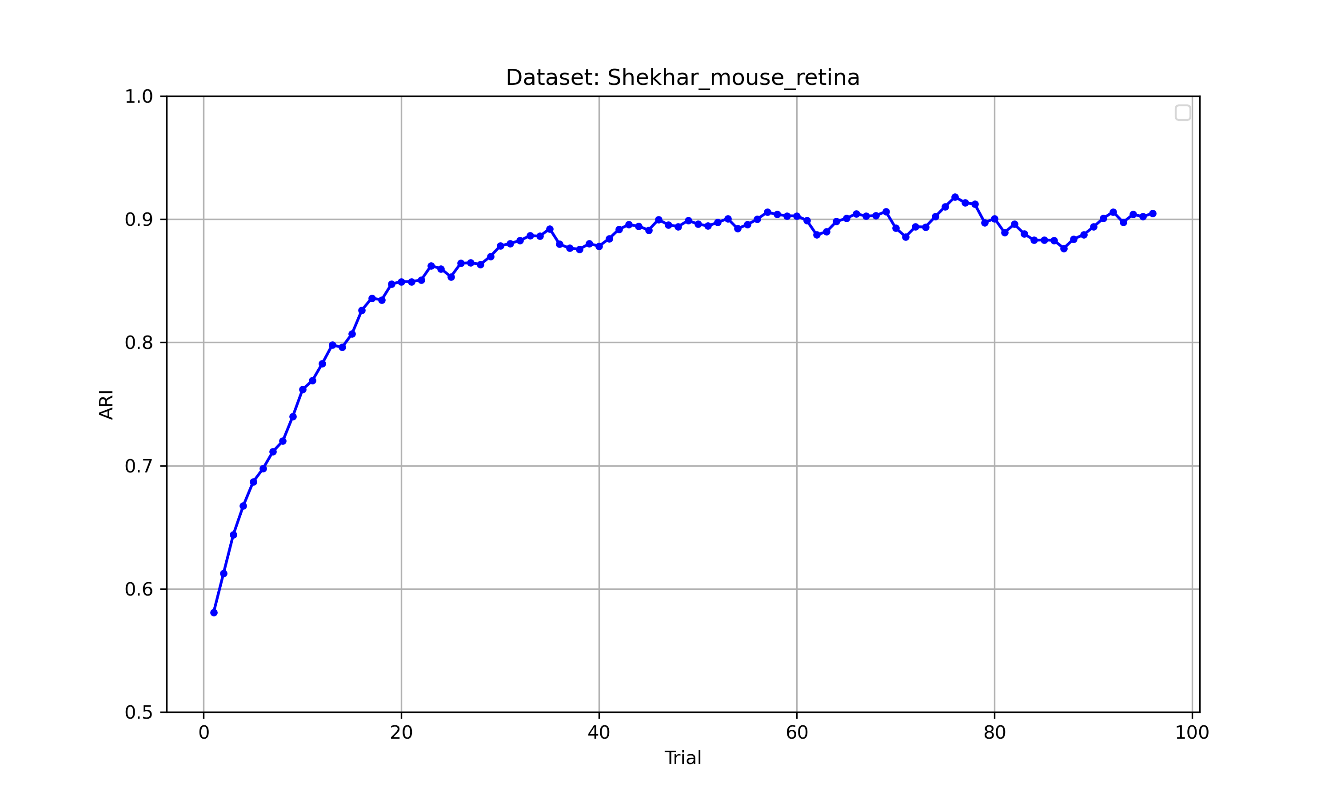


**Figure S4:** The optimization history for Shekhar_mouse_retina using Optuna.


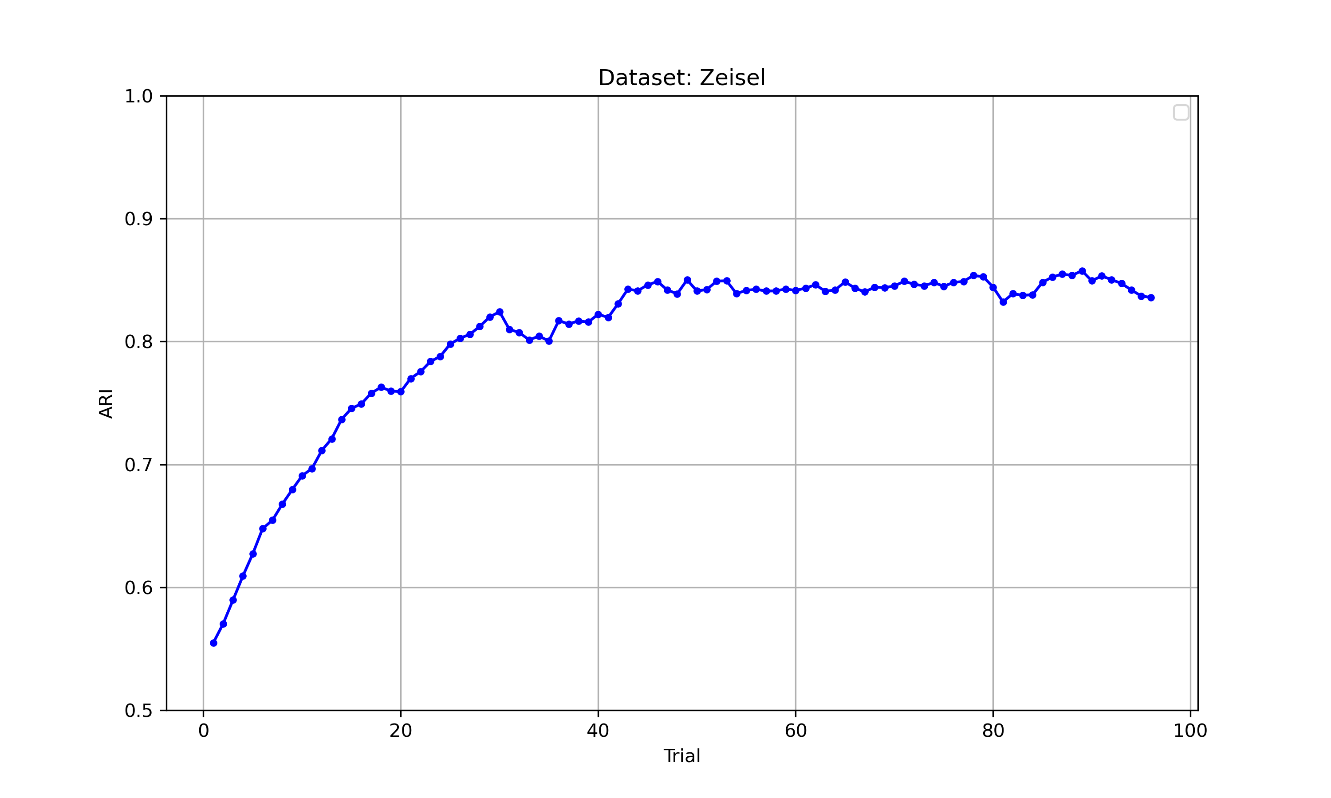


**Figure S4:** The optimization history for Zeisel using Optuna.


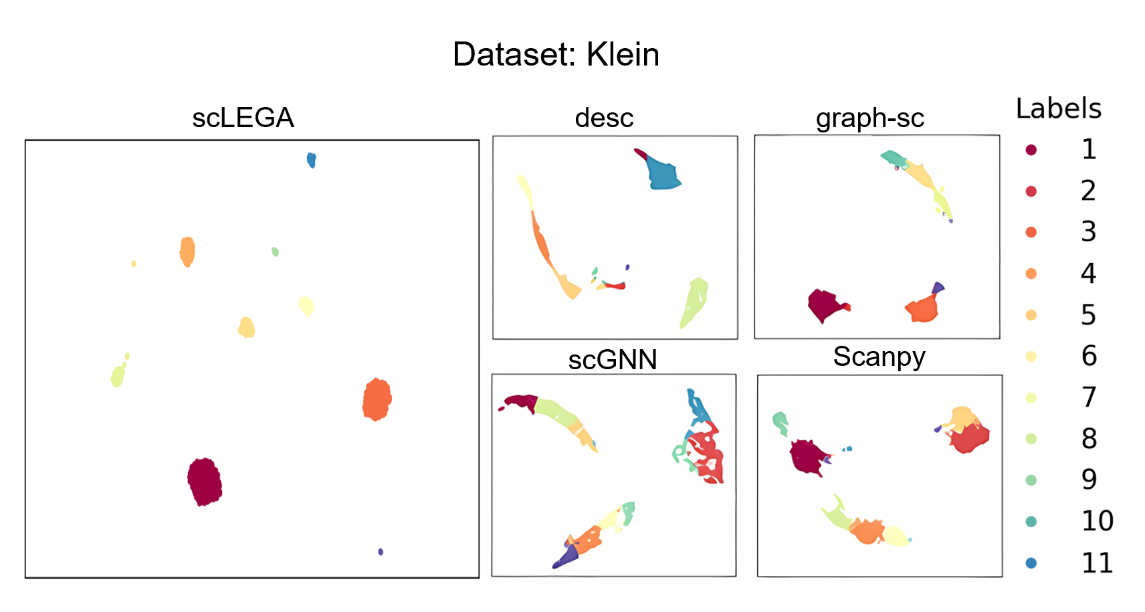


**Figure S6**: Comparison of UMAP visualization from four other deep models for the Klein dataset.


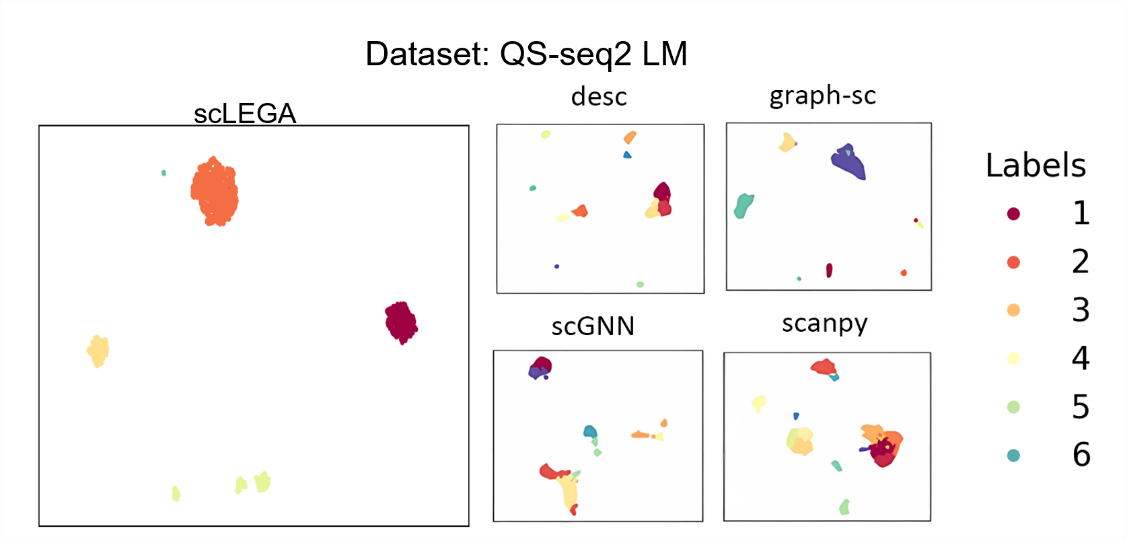


**Figure S7**: Comparison of UMAP visualization from four other deep models for the Quake_Smart-seq2_Limb_Muscle dataset.


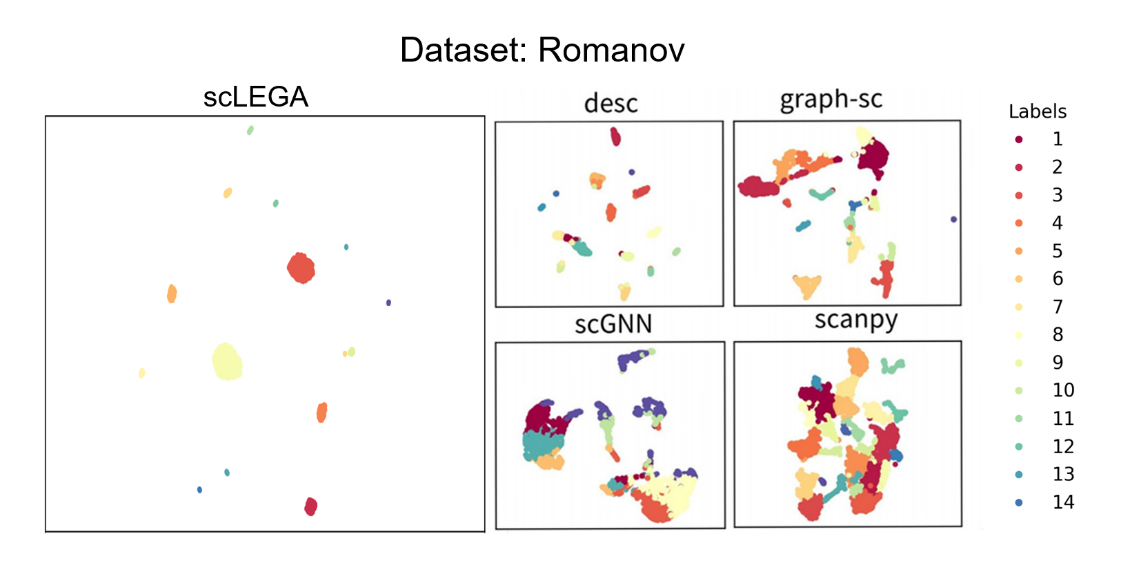


**Figure S8**: Comparison of UMAP visualization from four other deep models for the Romanov dataset.


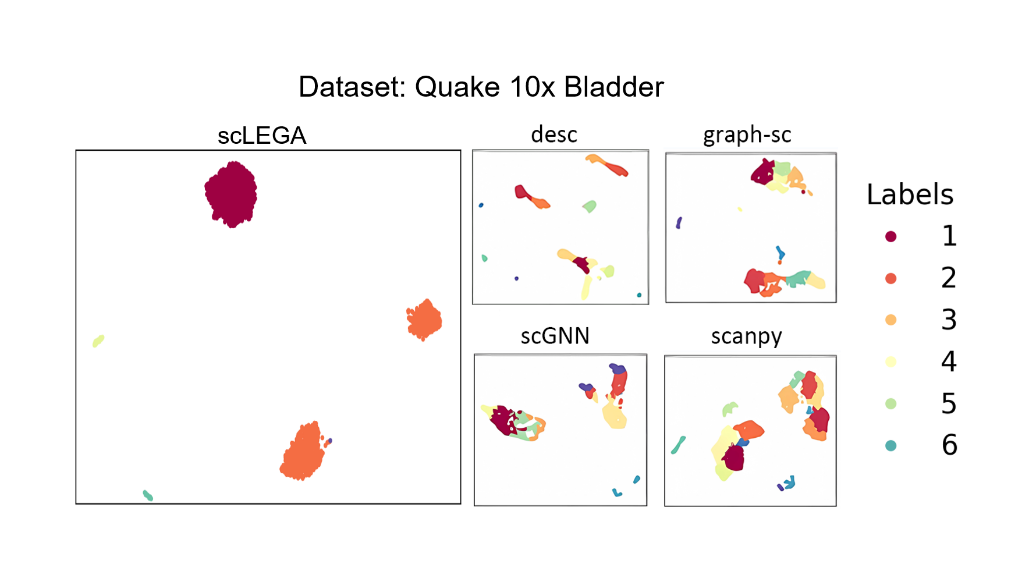


**Figure S9**: Comparison of UMAP visualization from four other deep models for the Quake_10x_Bladder dataset.


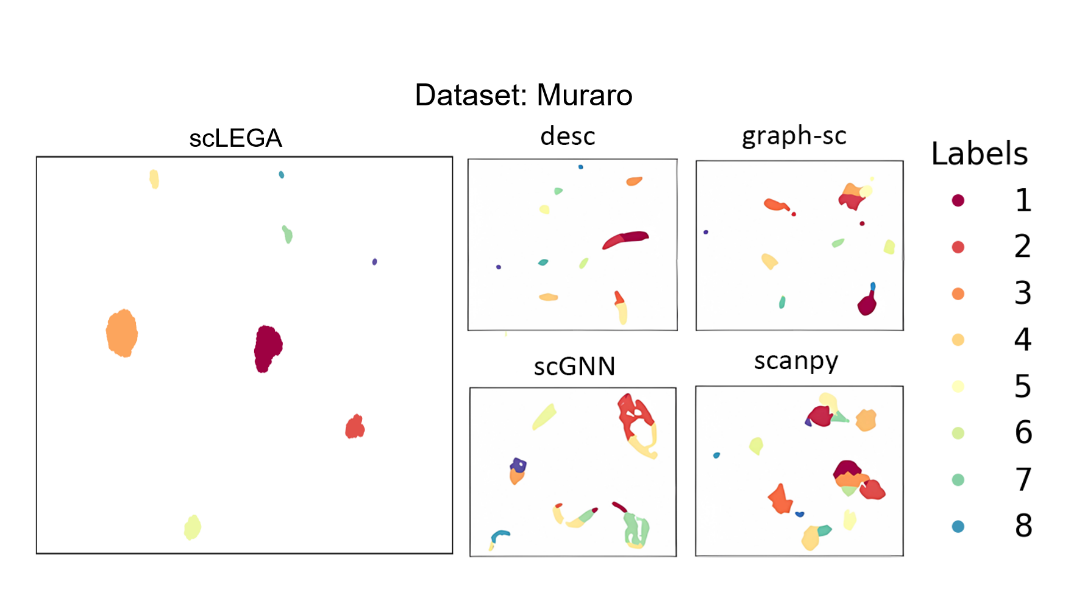


**Figure S10**: Comparison of UMAP visualization from four other deep models for the Muraro dataset.


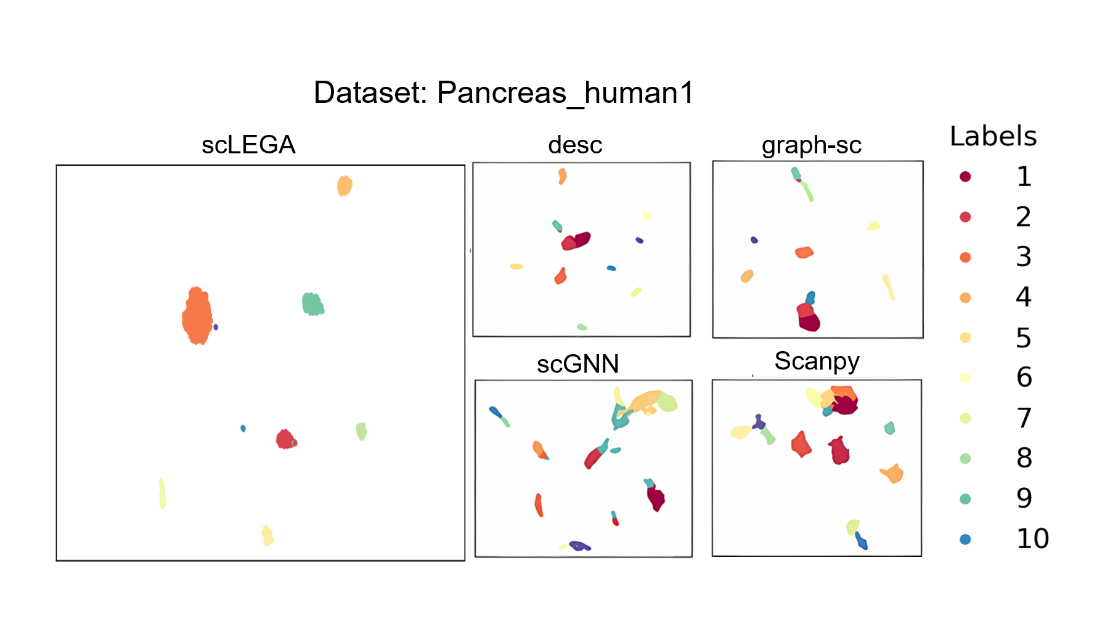


**Figure S11**: Comparison of UMAP visualization from four other deep models for the Pancreas_human1 dataset.


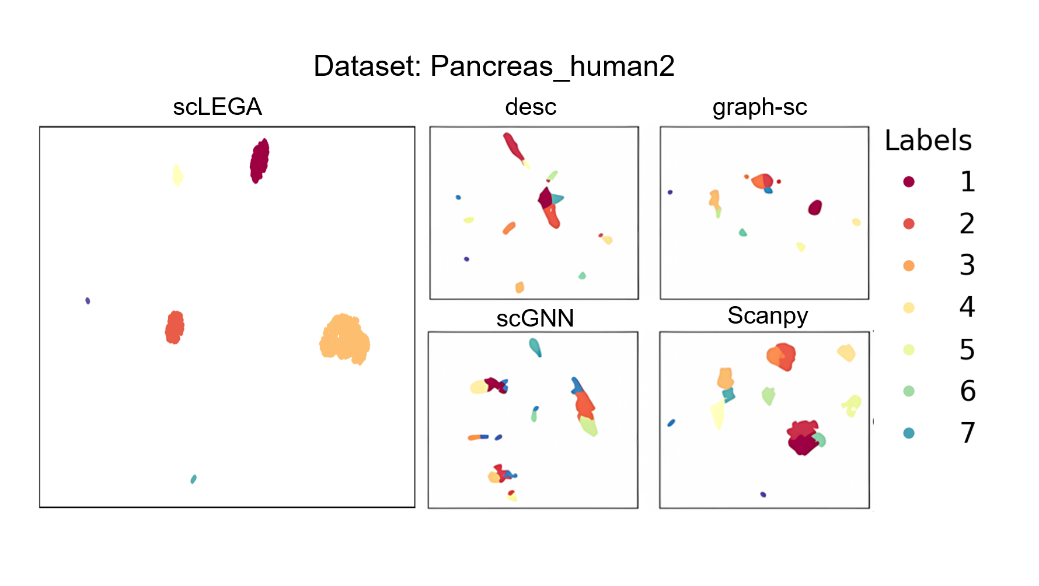


**Figure S12**: Comparison of UMAP visualization from four other deep models for the Pancreas_human2 dataset.


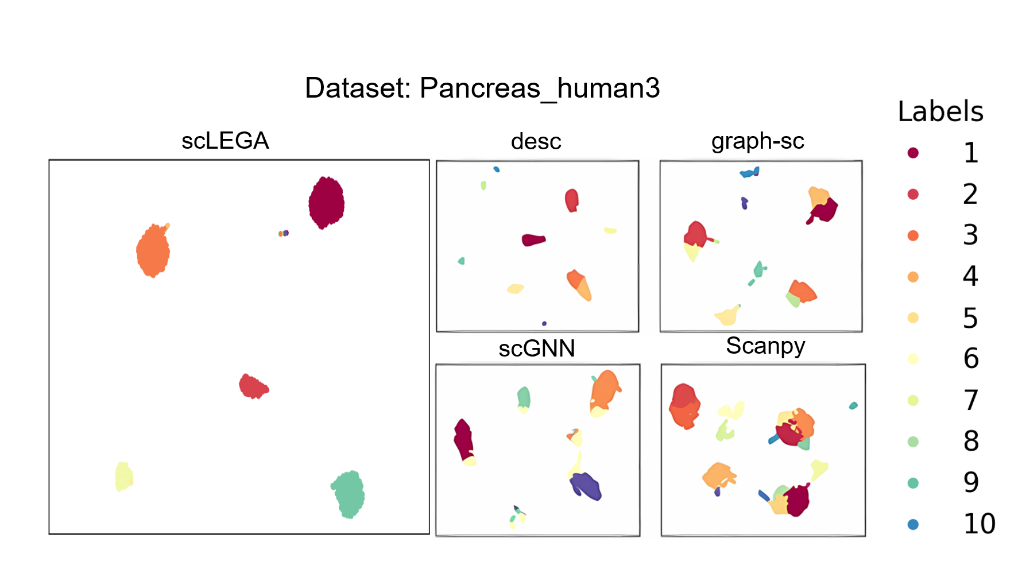


**Figure S13**: Comparison of UMAP visualization from four other deep models for the Pancreas_human3 dataset.


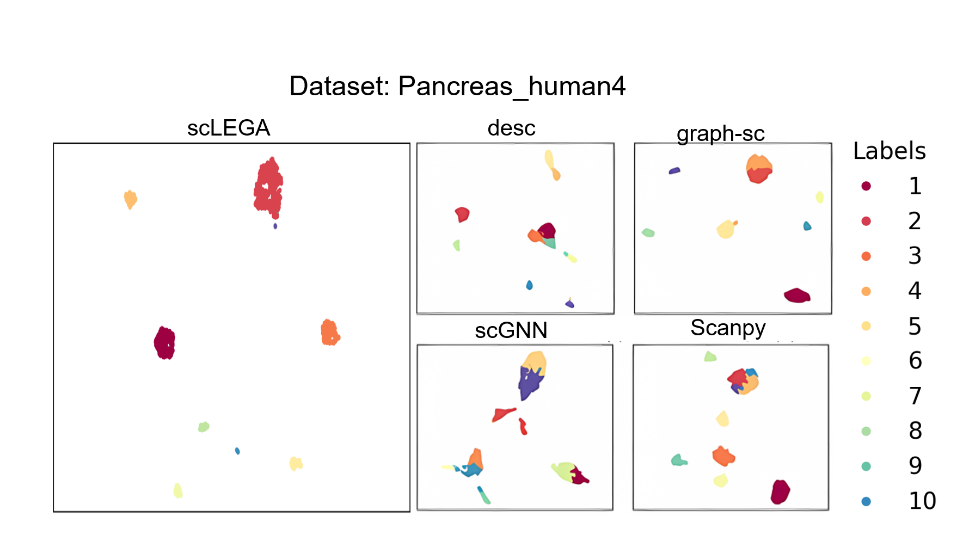


**Figure S14**: Comparison of UMAP visualization from four other deep models for the Pancreas_human4 dataset.


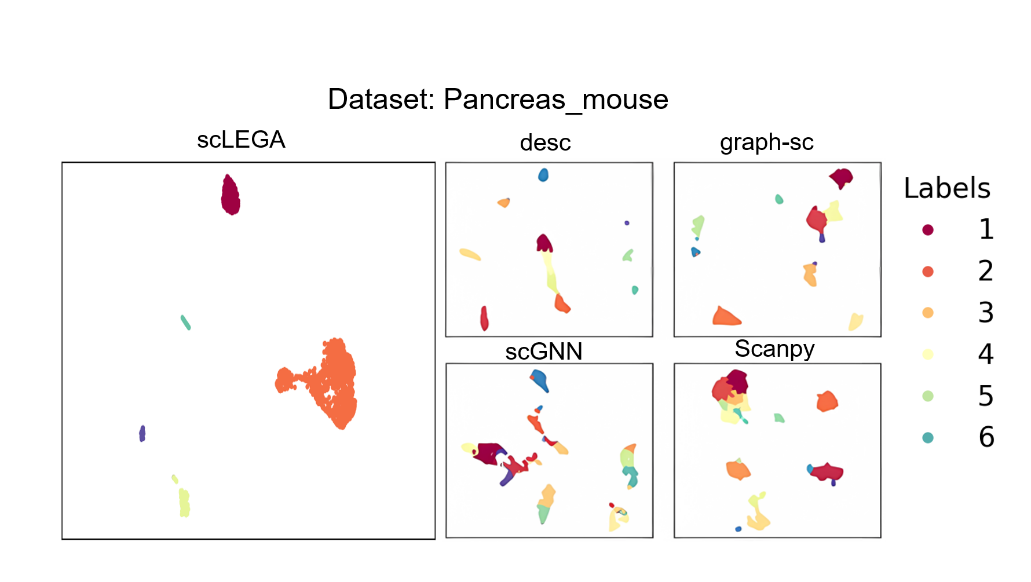


**Figure S15**: Comparison of UMAP visualization from four other deep models for the Pancreas_mouse dataset.


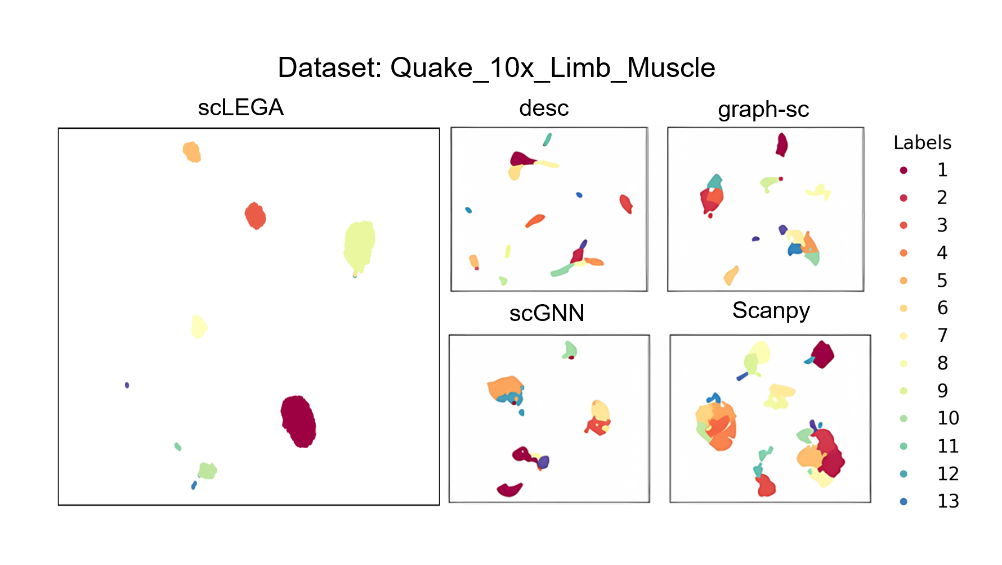


**Figure S16**: Comparison of UMAP visualization from four other deep models for the Quake_10x_Limb_Muscle dataset.


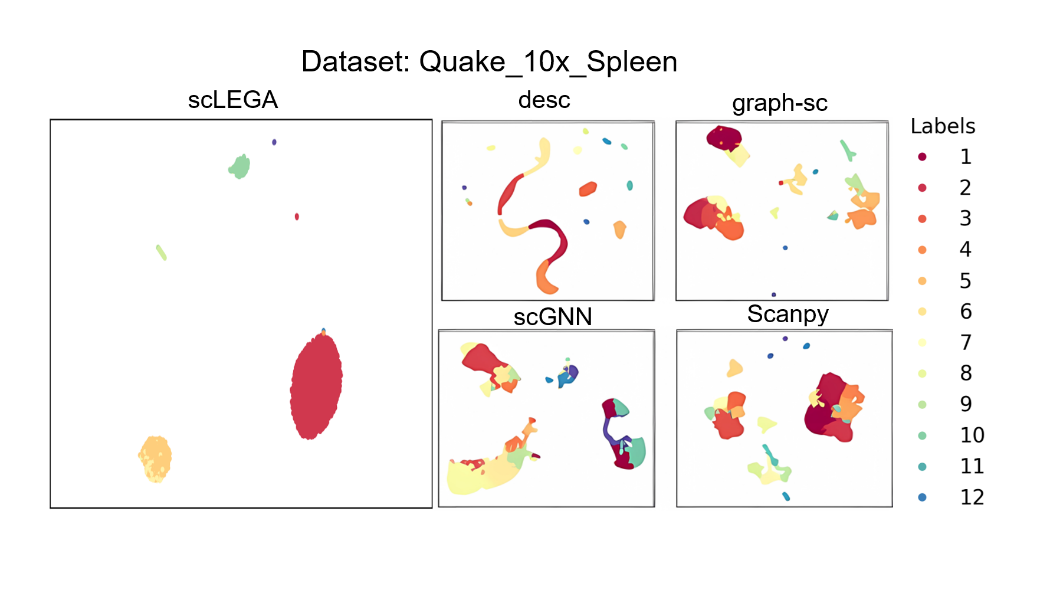


**Figure S17**: Comparison of UMAP visualization from four other deep models for the Quake_10x_Spleen dataset.


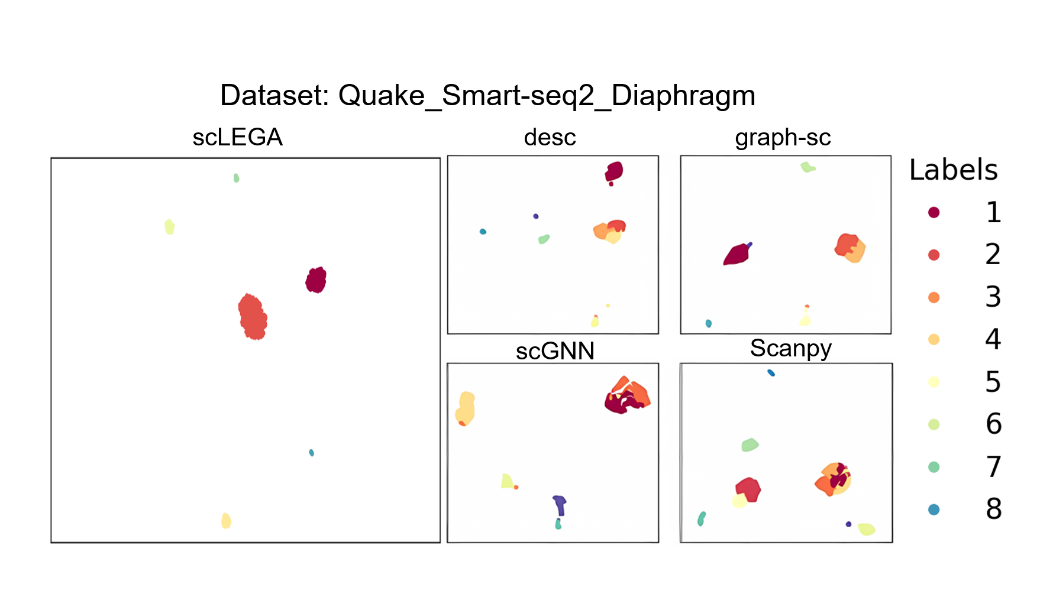


**Figure S18**: Comparison of UMAP visualization from four other deep models for the Quake_Smart-seq2_Diaphragm dataset.


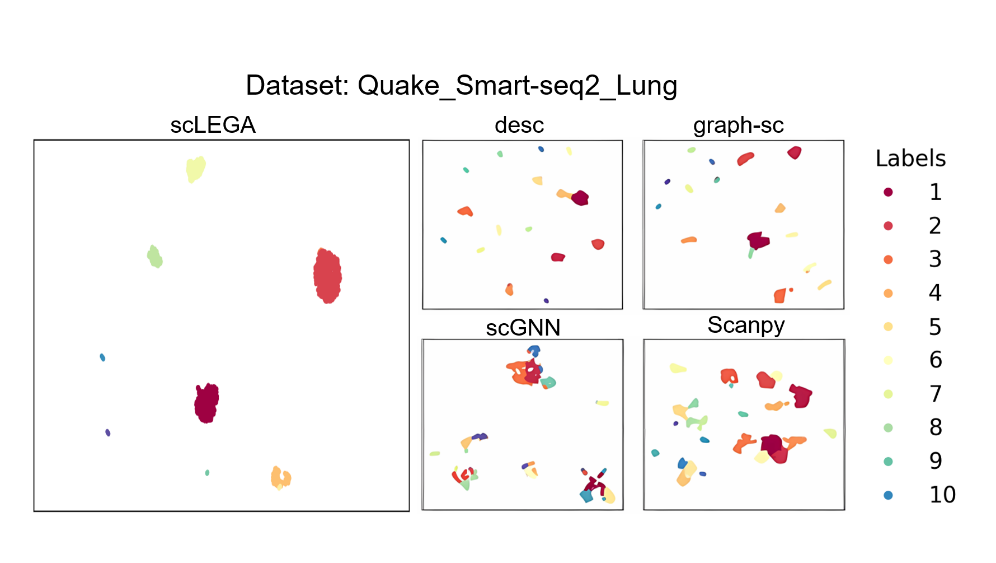


**Figure S19**: Comparison of UMAP visualization from four other deep models for the Quake_Smart-seq2_Lung dataset.


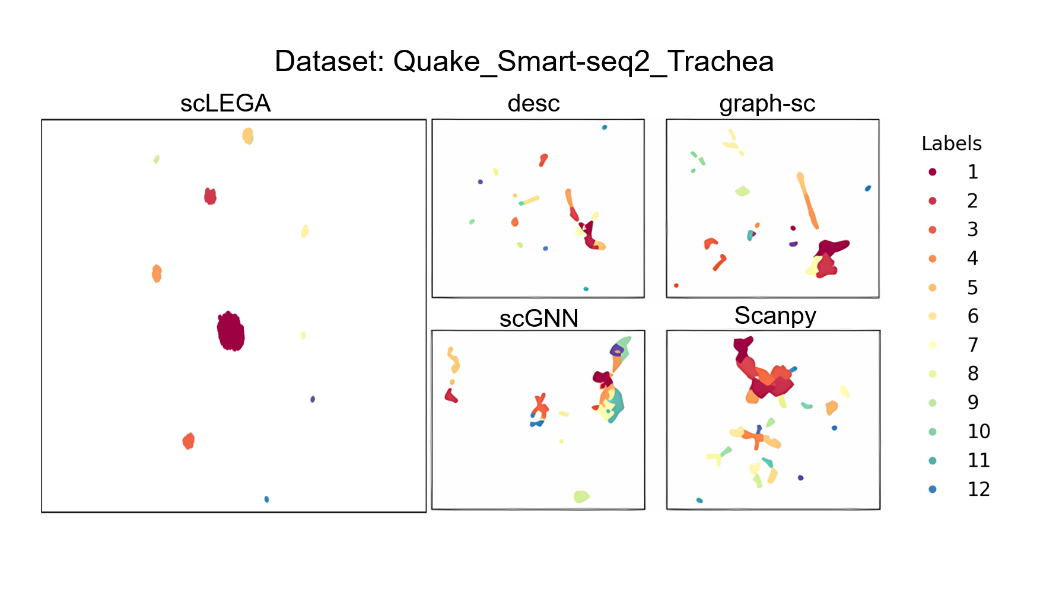


**Figure S20**: Comparison of UMAP visualization from four other deep models for the Quake_Smart-seq2_Trachea dataset.


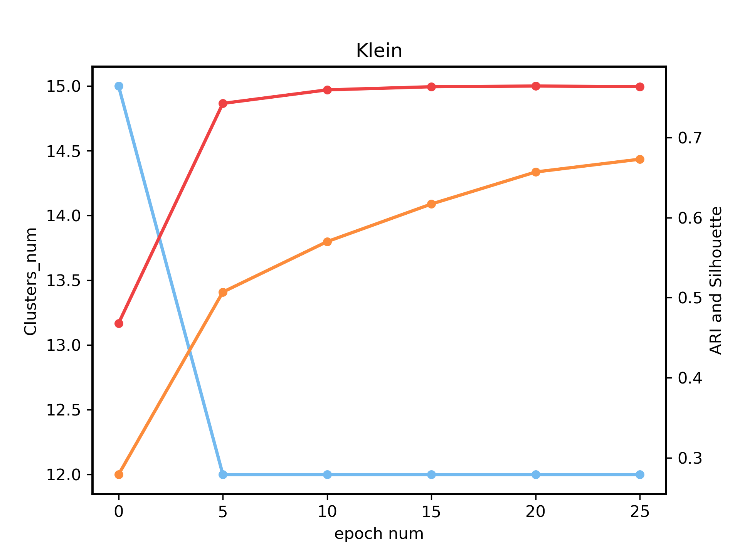


**Figure S21**: In the clustering stage, the cluster center adaptively adjusts with the training epochs, and the change of ARI and Silhouette Coefficient with the training epochs for Klein dataset.


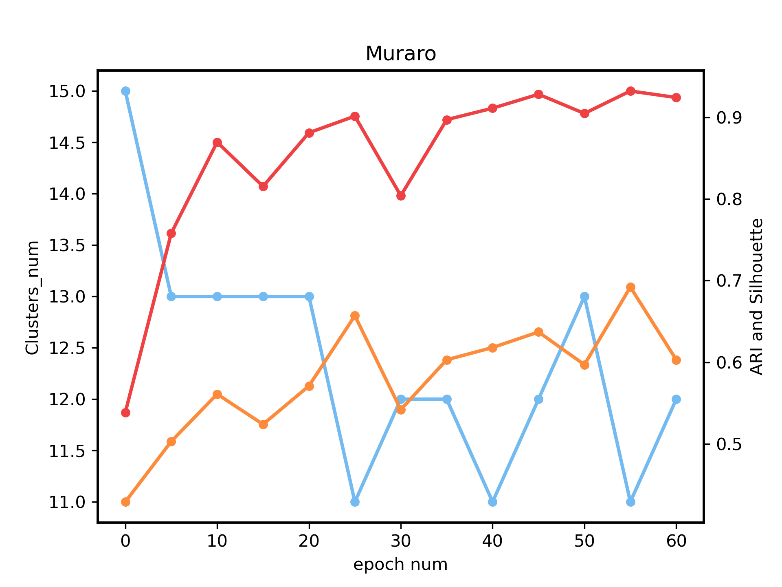


**Figure S22**: In the clustering stage, the cluster center adaptively adjusts with the training epochs, and the change of ARI and Silhouette Coefficient with the training epochs for Muraro dataset.


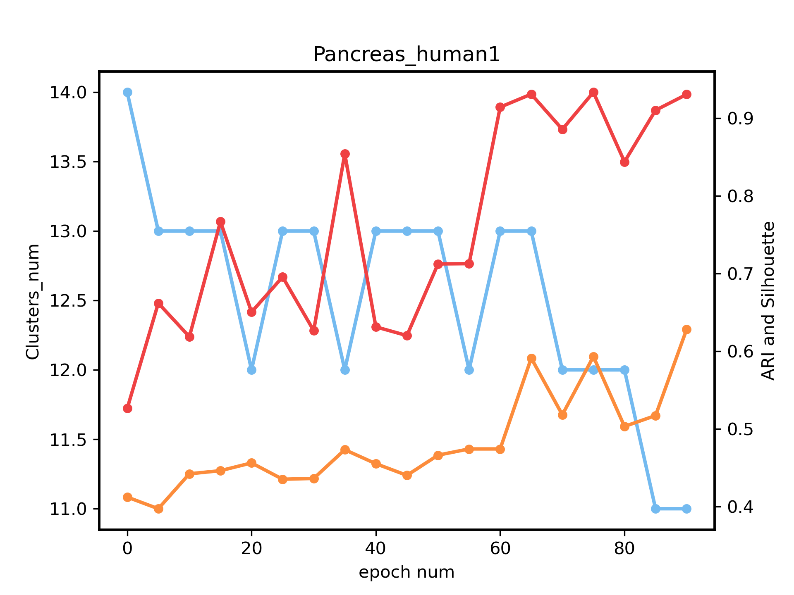


**Figure S23**: In the clustering stage, the cluster center adaptively adjusts with the training epochs, and the change of ARI and Silhouette Coefficient with the training epochs for Pancreas_human1 dataset.


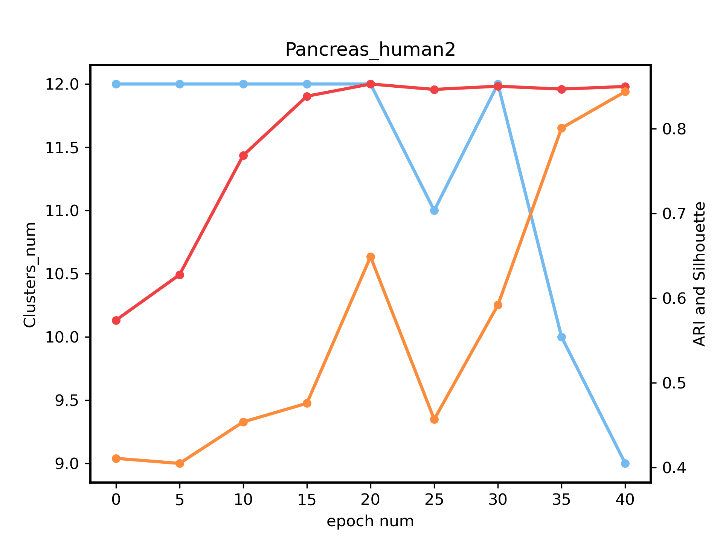


**Figure S24**: In the clustering stage, the cluster center adaptively adjusts with the training epochs, and the change of ARI and Silhouette Coefficient with the training epochs for Pancreas_human2 dataset.


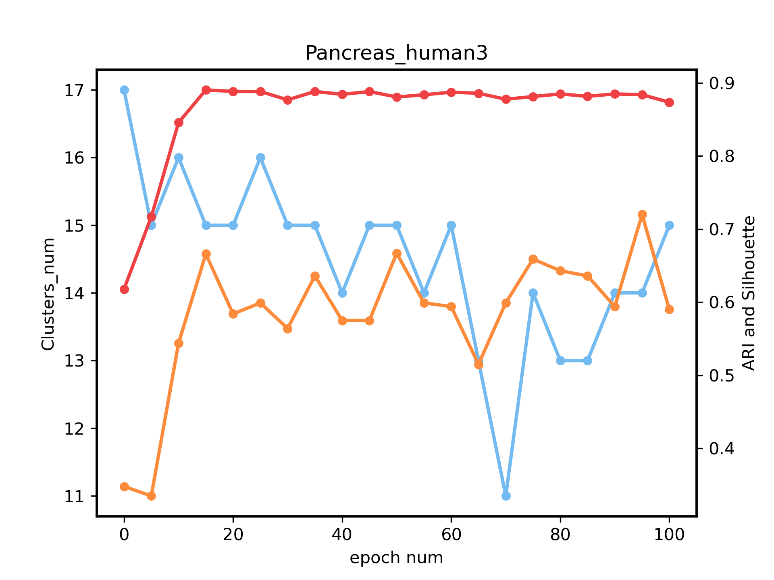


**Figure S25**: In the clustering stage, the cluster center adaptively adjusts with the training epochs, and the change of ARI and Silhouette Coefficient with the training epochs for Pancreas_human3 dataset.


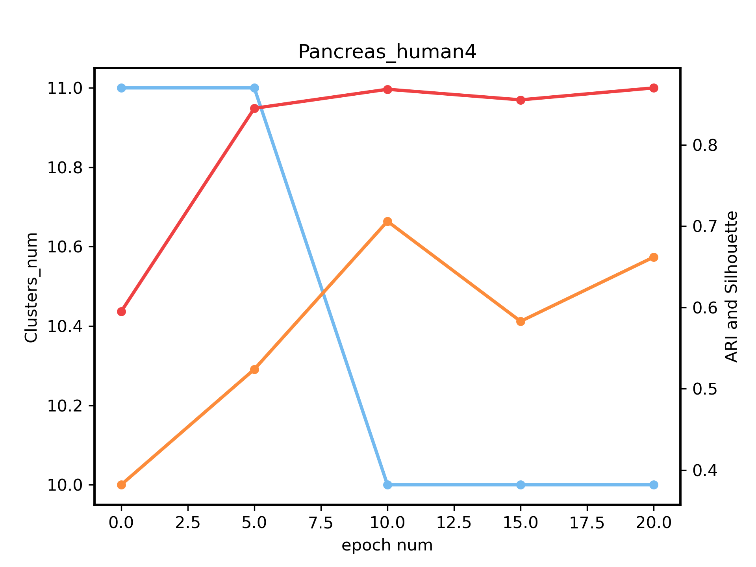


**Figure S26**: In the clustering stage, the cluster center adaptively adjusts with the training epochs, and the change of ARI and Silhouette Coefficient with the training epochs for Pancreas_human4 dataset.


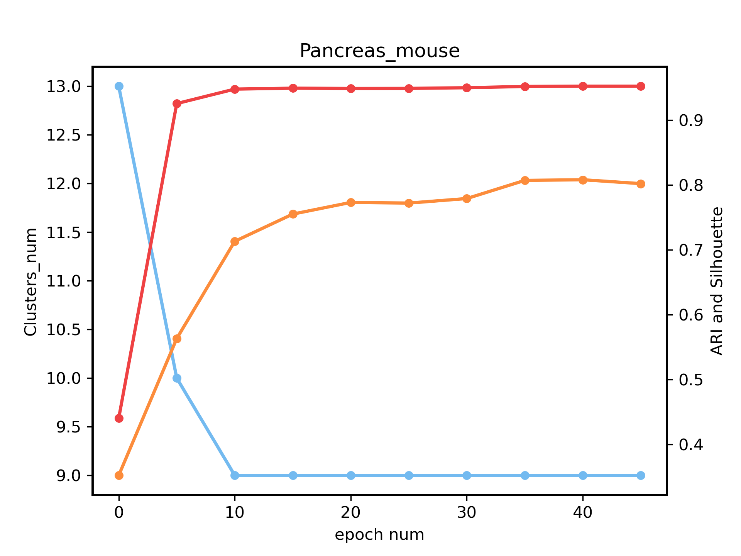


**Figure S27**: In the clustering stage, the cluster center adaptively adjusts with the training epochs, and the change of ARI and Silhouette Coefficient with the training epochs for Pancreas_mouse dataset.


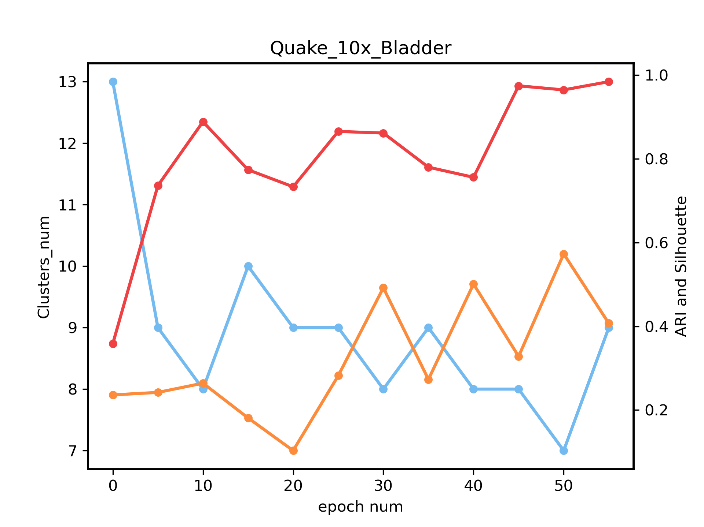


**Figure S28**: In the clustering stage, the cluster center adaptively adjusts with the training epochs, and the change of ARI and Silhouette Coefficient with the training epochs for Quake_10x_Bladder dataset.


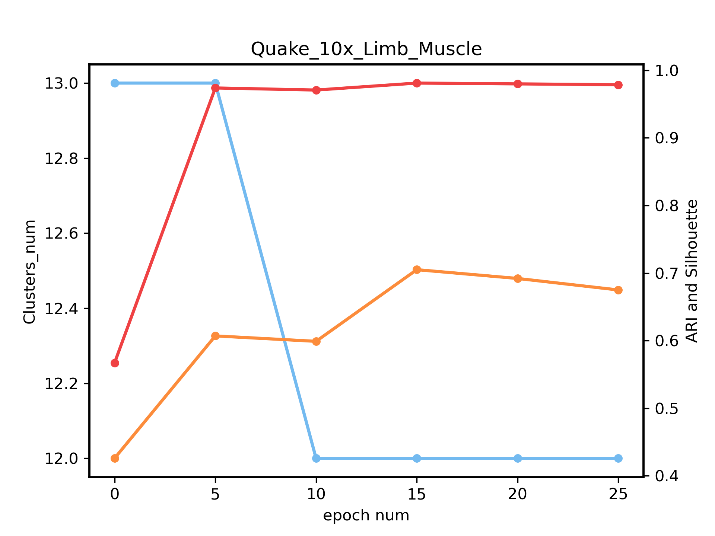


**Figure S29**: In the clustering stage, the cluster center adaptively adjusts with the training epochs, and the change of ARI and Silhouette Coefficient with the training epochs for Quake_10x_Limb_Muscle dataset.


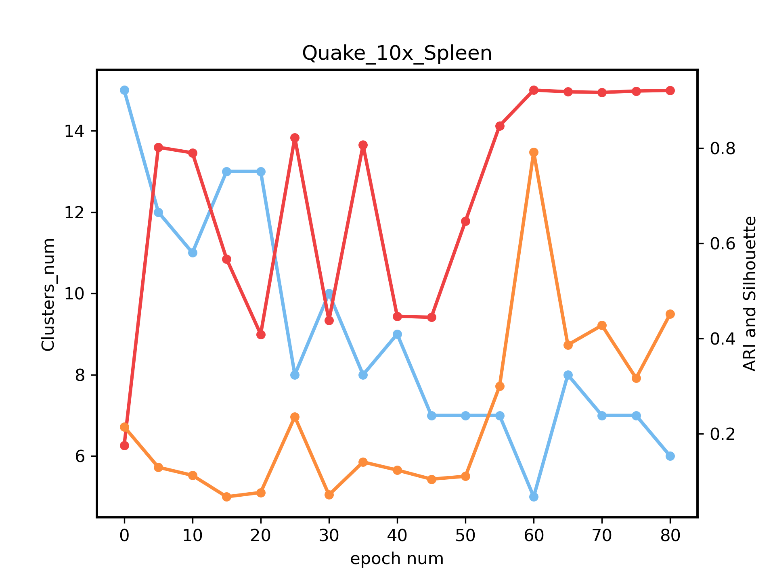


**Figure S30**: In the clustering stage, the cluster center adaptively adjusts with the training epochs, and the change of ARI and Silhouette Coefficient with the training epochs for Quake_10x_Spleen dataset.


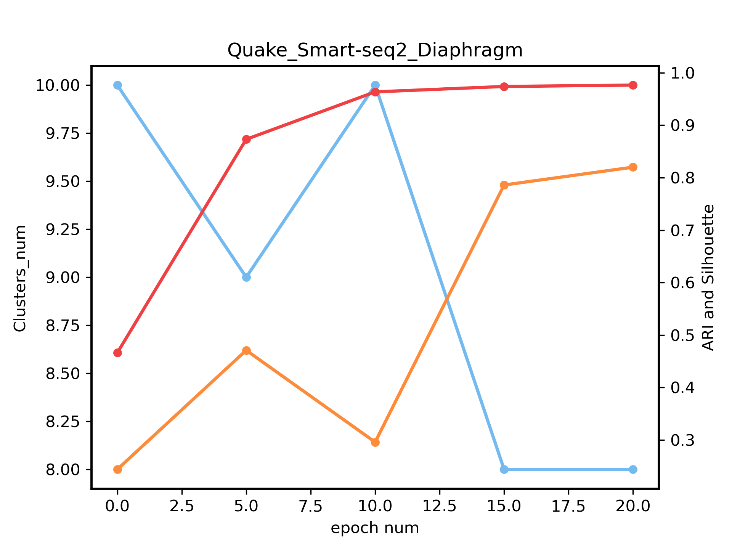


**Figure S31**: In the clustering stage, the cluster center adaptively adjusts with the training epochs, and the change of ARI and Silhouette Coefficient with the training epochs for Quake_Smart-seq2_Diaphragm dataset.


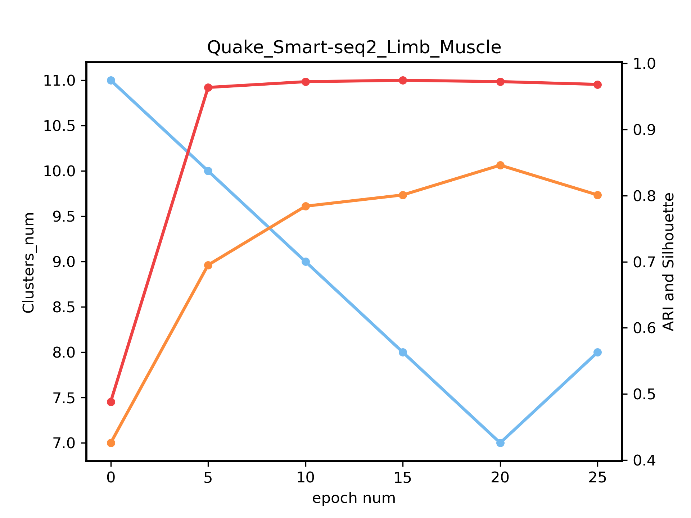


**Figure S32**: In the clustering stage, the cluster center adaptively adjusts with the training epochs, and the change of ARI and Silhouette Coefficient with the training epochs for Quake_Smart-seq2_Limb_Muscle dataset.


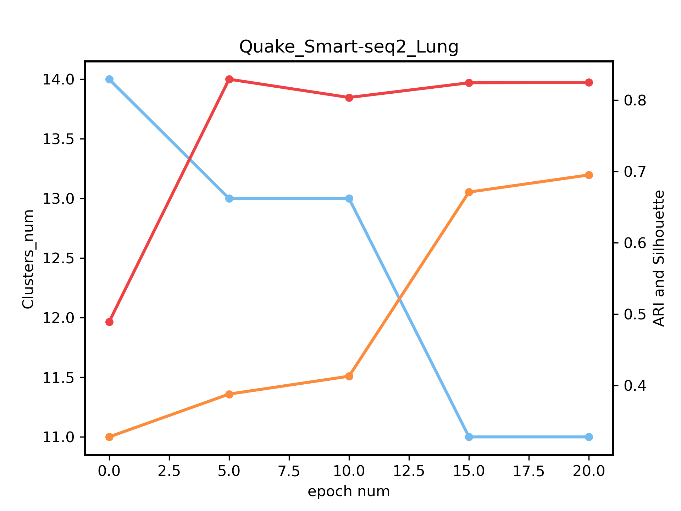


**Figure S33**: In the clustering stage, the cluster center adaptively adjusts with the training epochs, and the change of ARI and Silhouette Coefficient with the training epochs for Quake_Smart-seq2_Lung dataset.


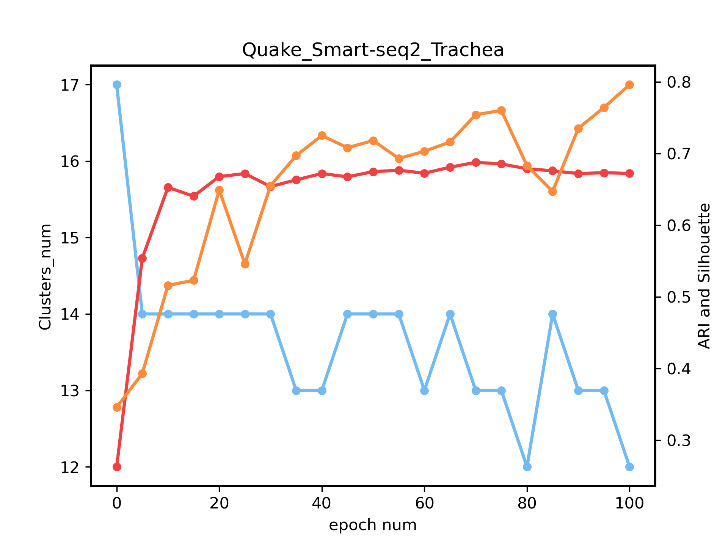


**Figure S34**: In the clustering stage, the cluster center adaptively adjusts with the training epochs, and the change of ARI and Silhouette Coefficient with the training epochs for Quake_Smart-seq2_Trachea dataset.


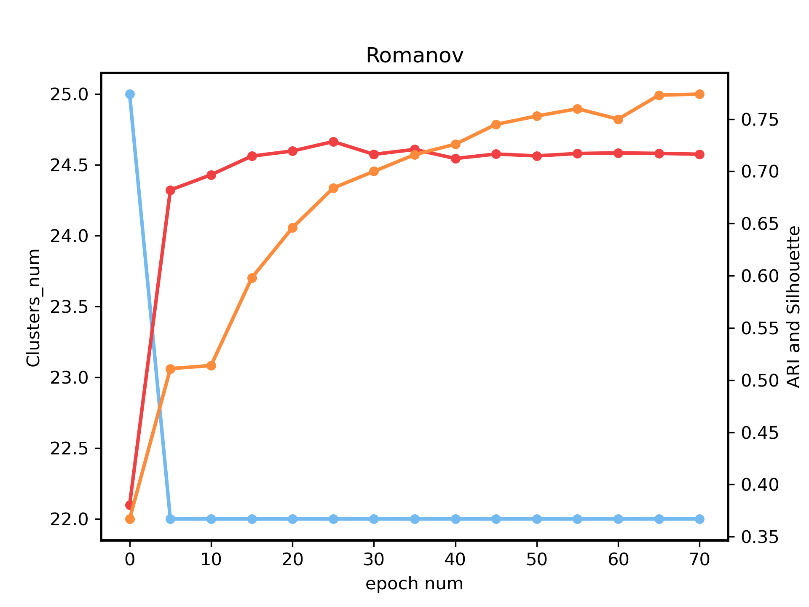


**Figure S35**: In the clustering stage, the cluster center adaptively adjusts with the training epochs, and the change of ARI and Silhouette Coefficient with the training epochs for Romanov dataset.
