# Supplementary figures and images for: scLEGA: an attention-based deep clustering method with a tendency for low expression of genes on single-cell RNA-seq data
Source: Brief Bioinform. 2024 Jul 26;25(5):bbae371. doi: 10.1093/bib/bbae371 (PMC11281828; doi:10.1093/bib/bbae371)

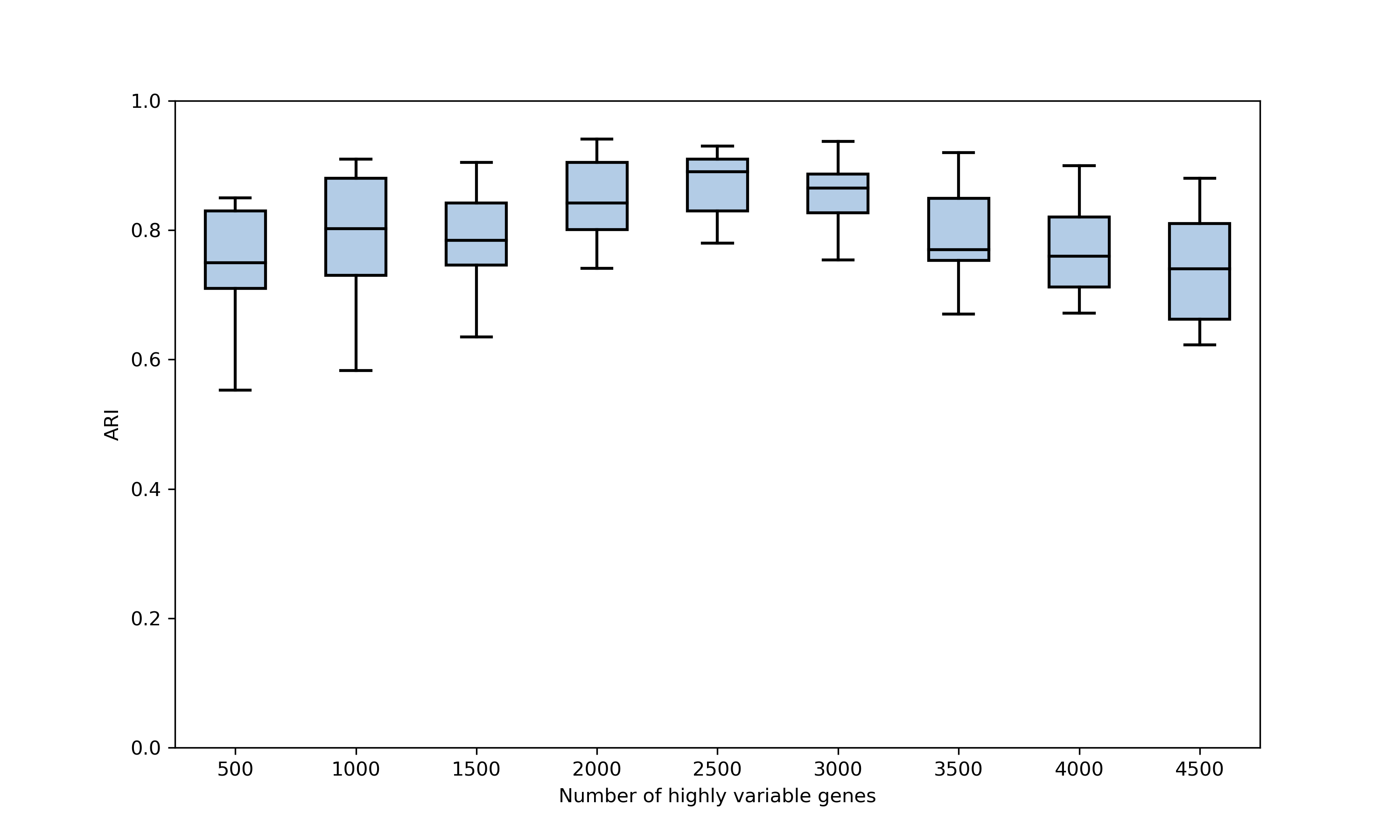

Supplement: bbae371 [file bbae371.zip › Figure S1.tif]

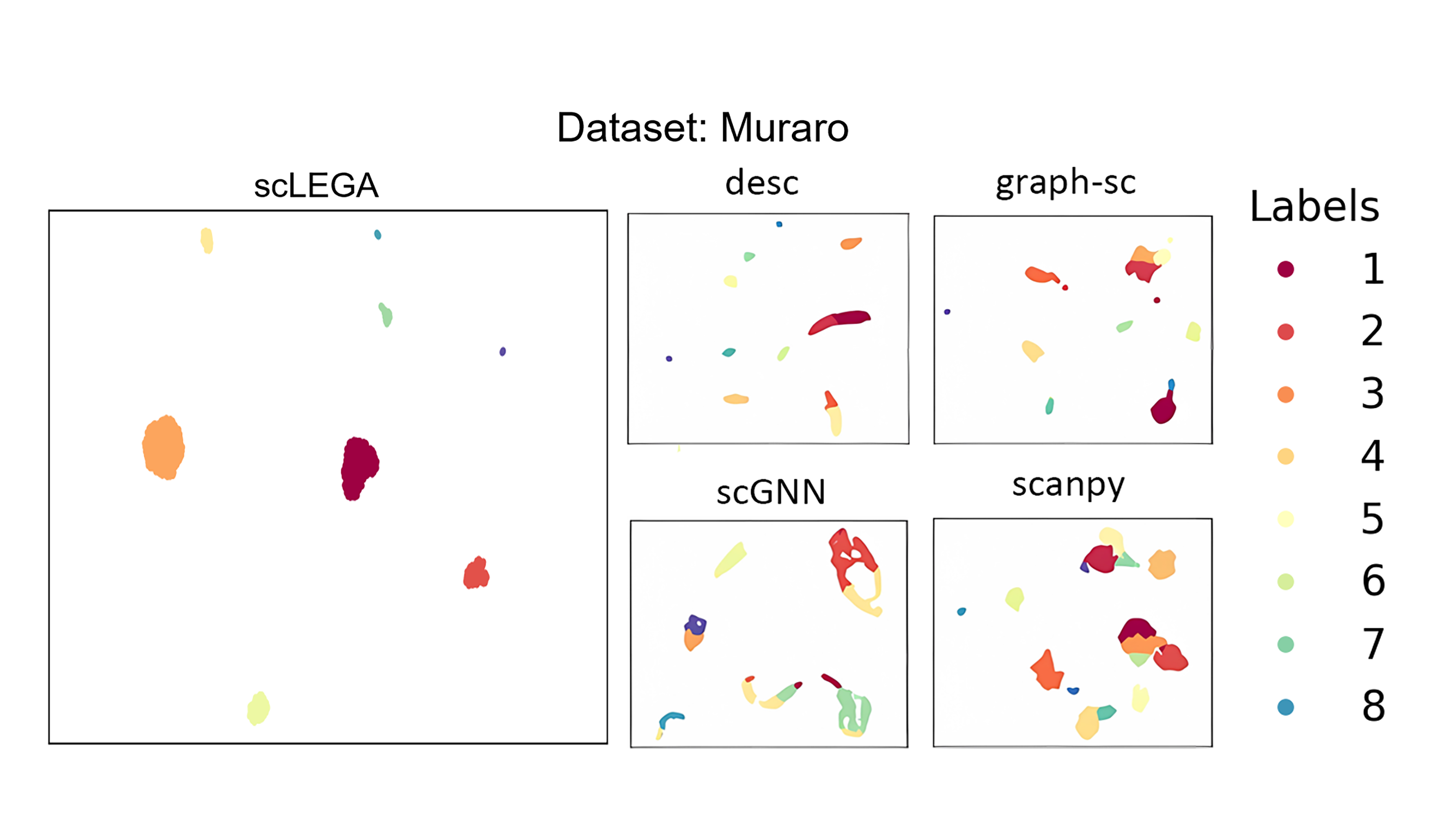

Supplement: bbae371 [file bbae371.zip › Figure S10.tif]

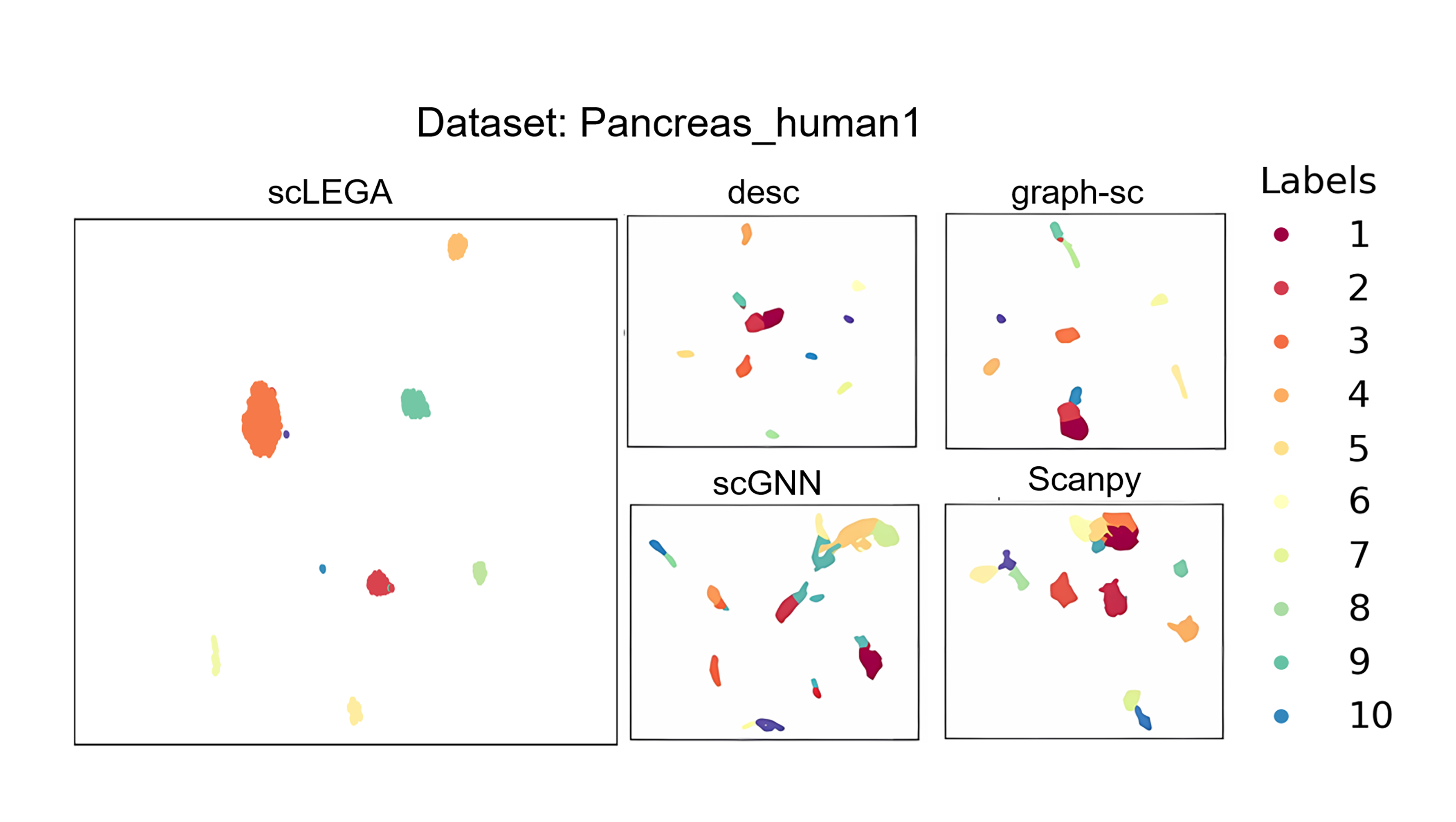

Supplement: bbae371 [file bbae371.zip › Figure S11.tif]

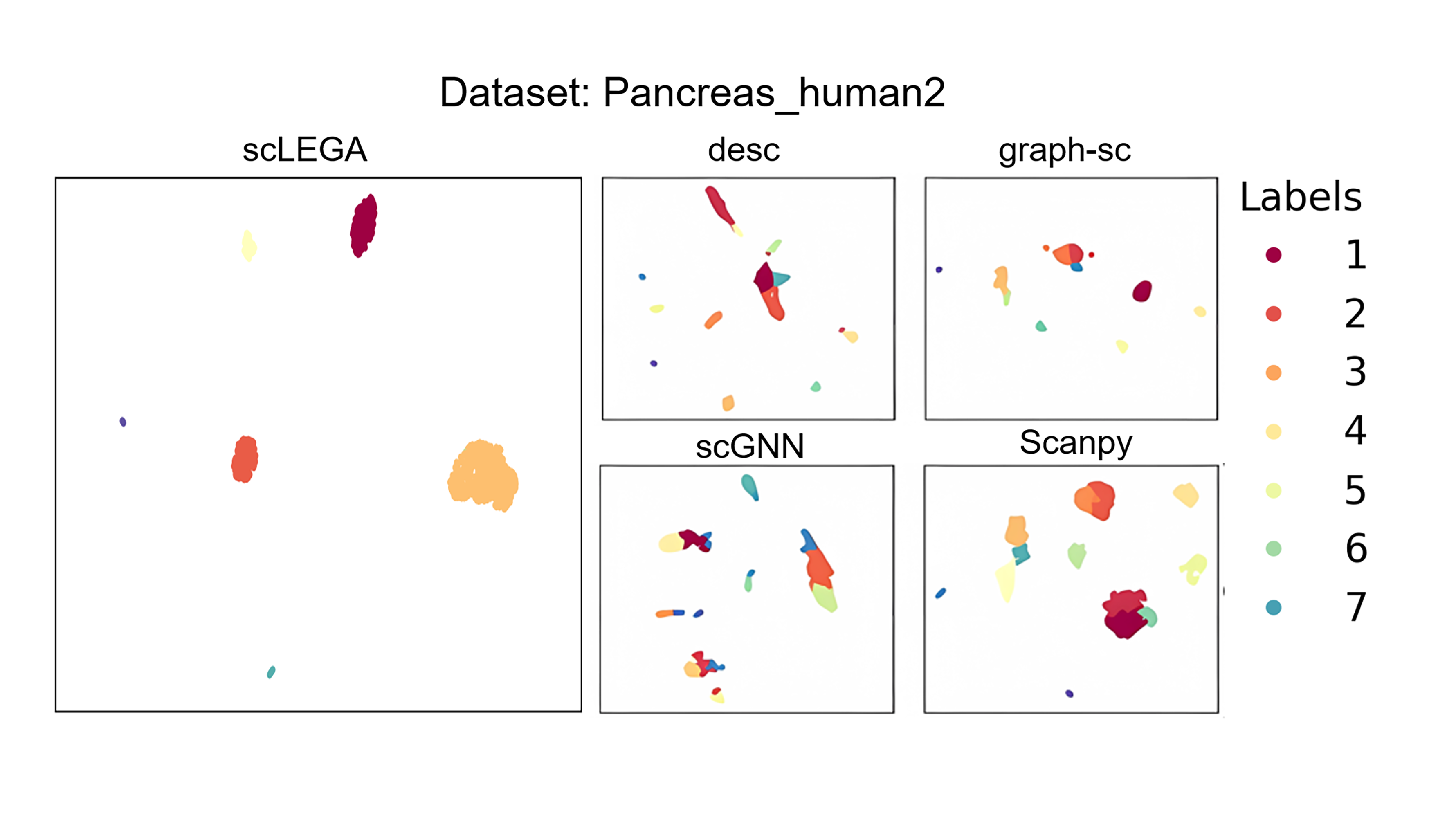

Supplement: bbae371 [file bbae371.zip › Figure S12.tif]

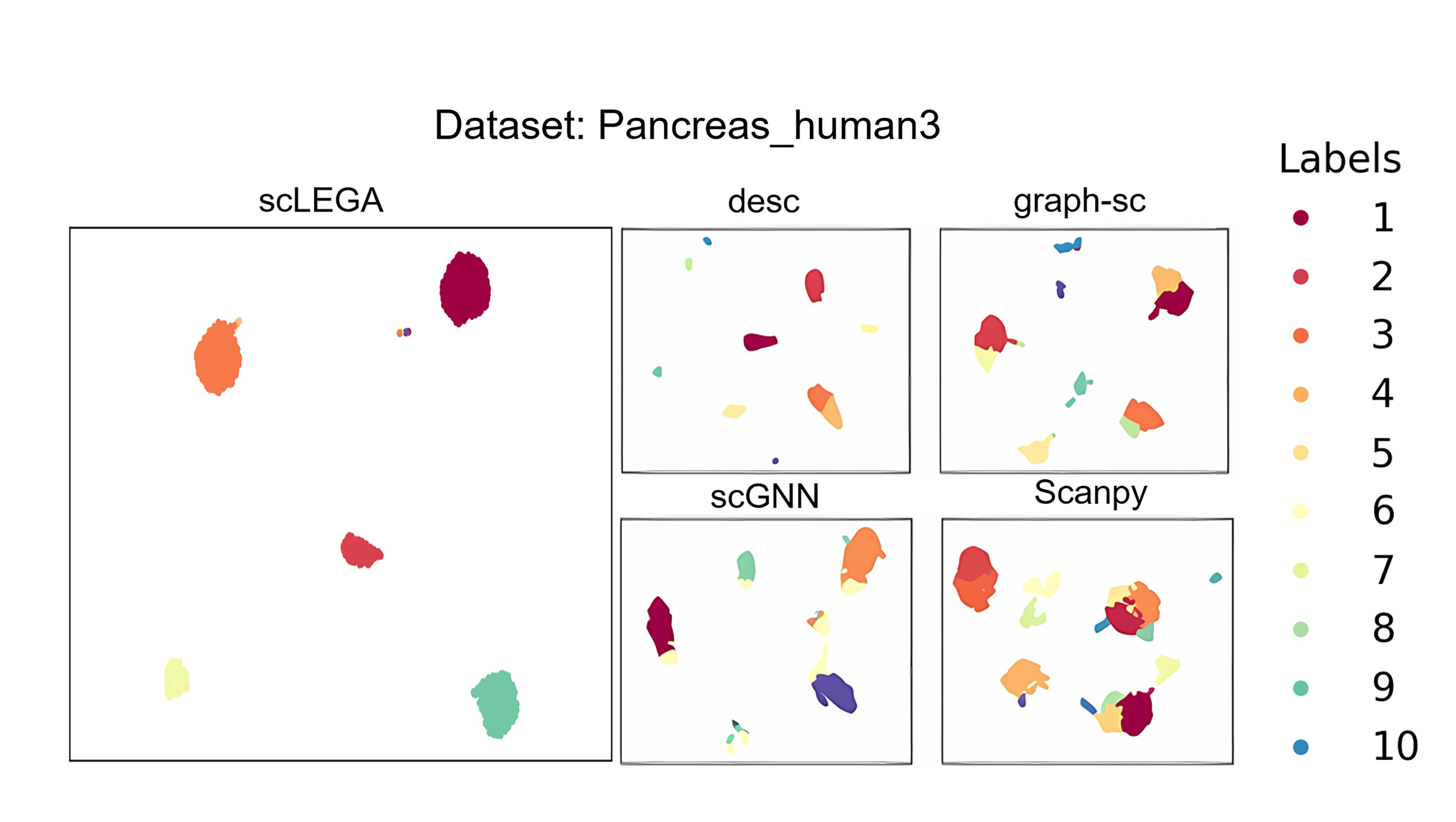

Supplement: bbae371 [file bbae371.zip › Figure S13.tif]

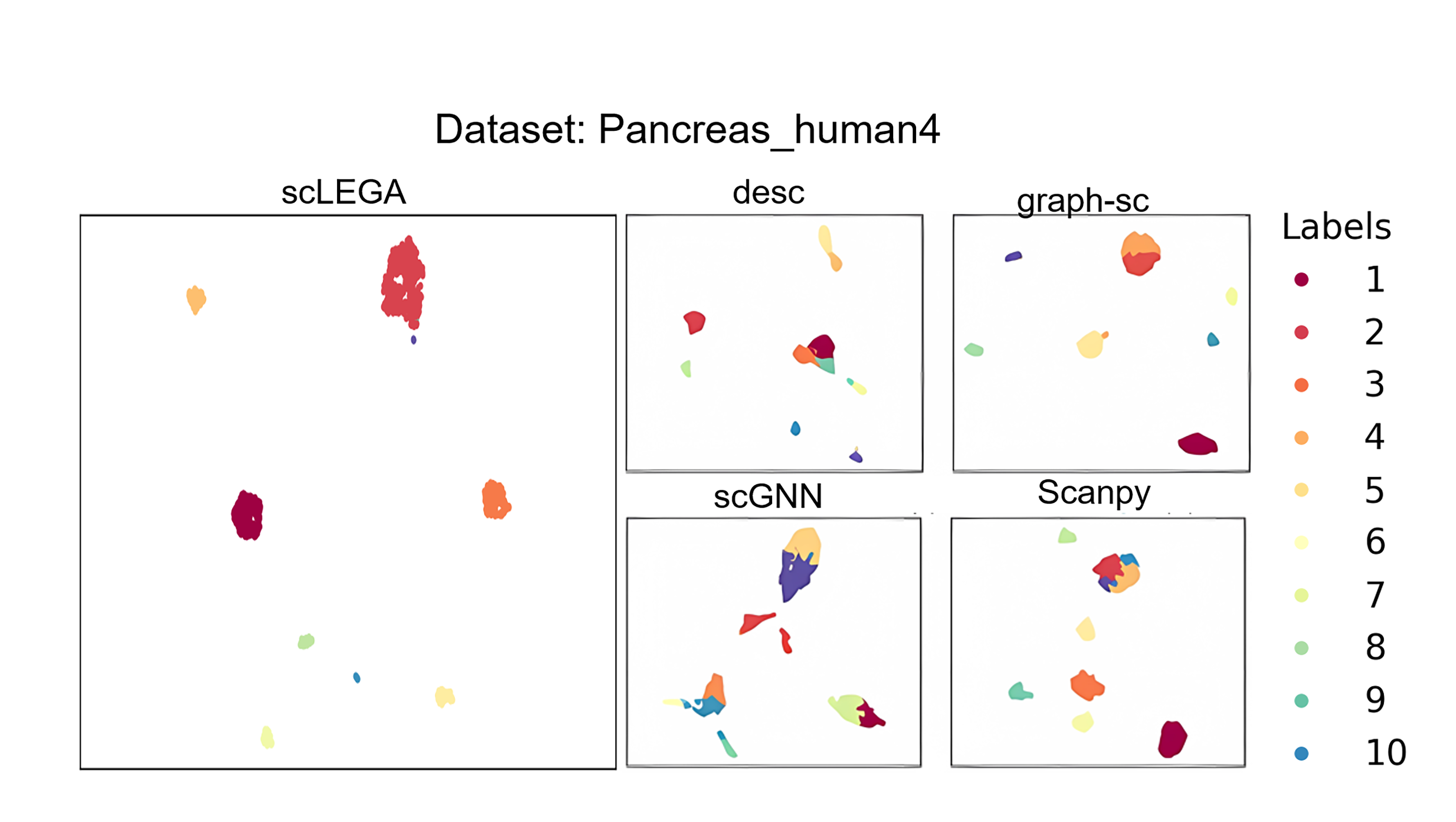

Supplement: bbae371 [file bbae371.zip › Figure S14.tif]

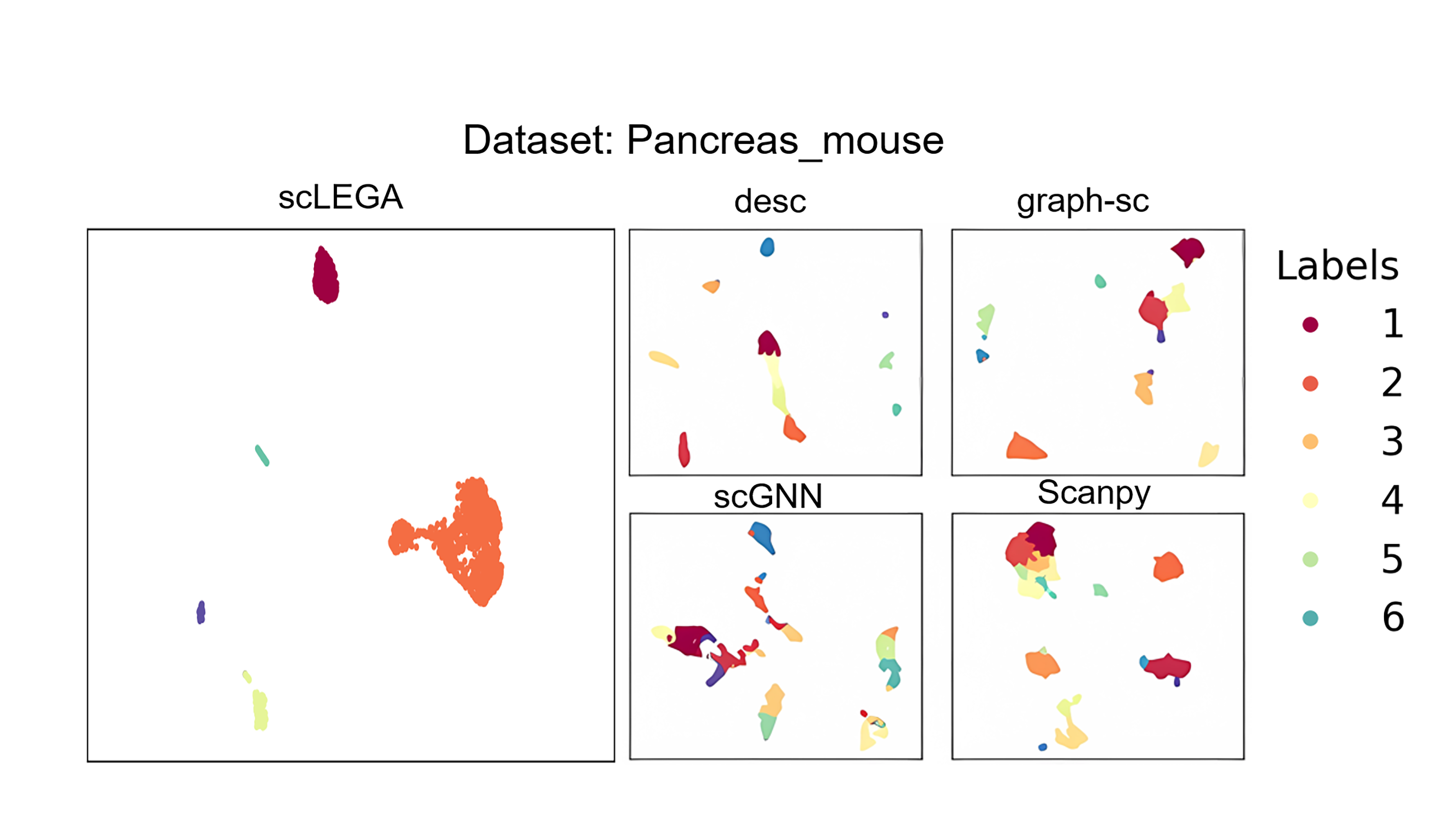

Supplement: bbae371 [file bbae371.zip › Figure S15.tif]

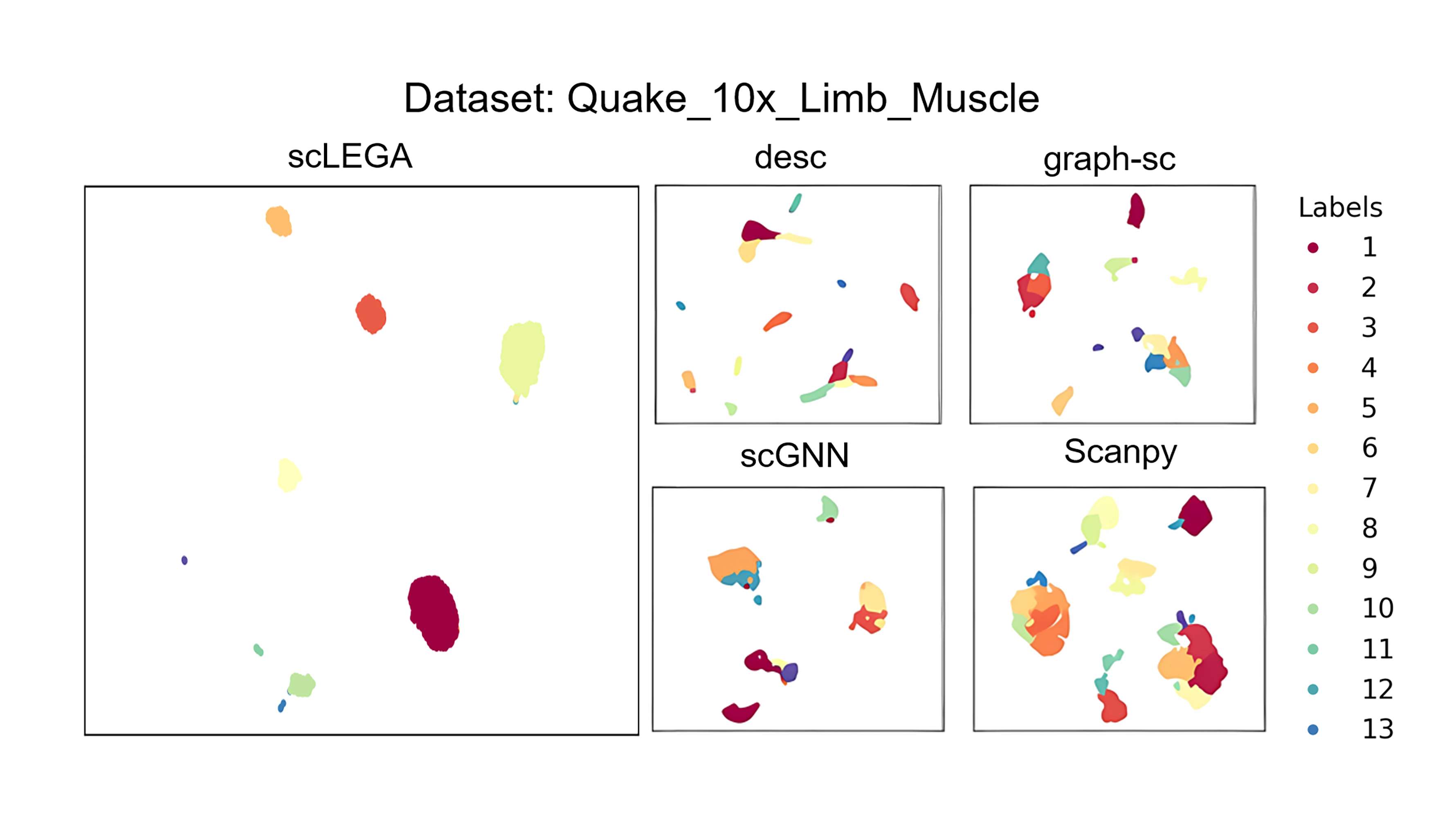

Supplement: bbae371 [file bbae371.zip › Figure S16.tif]

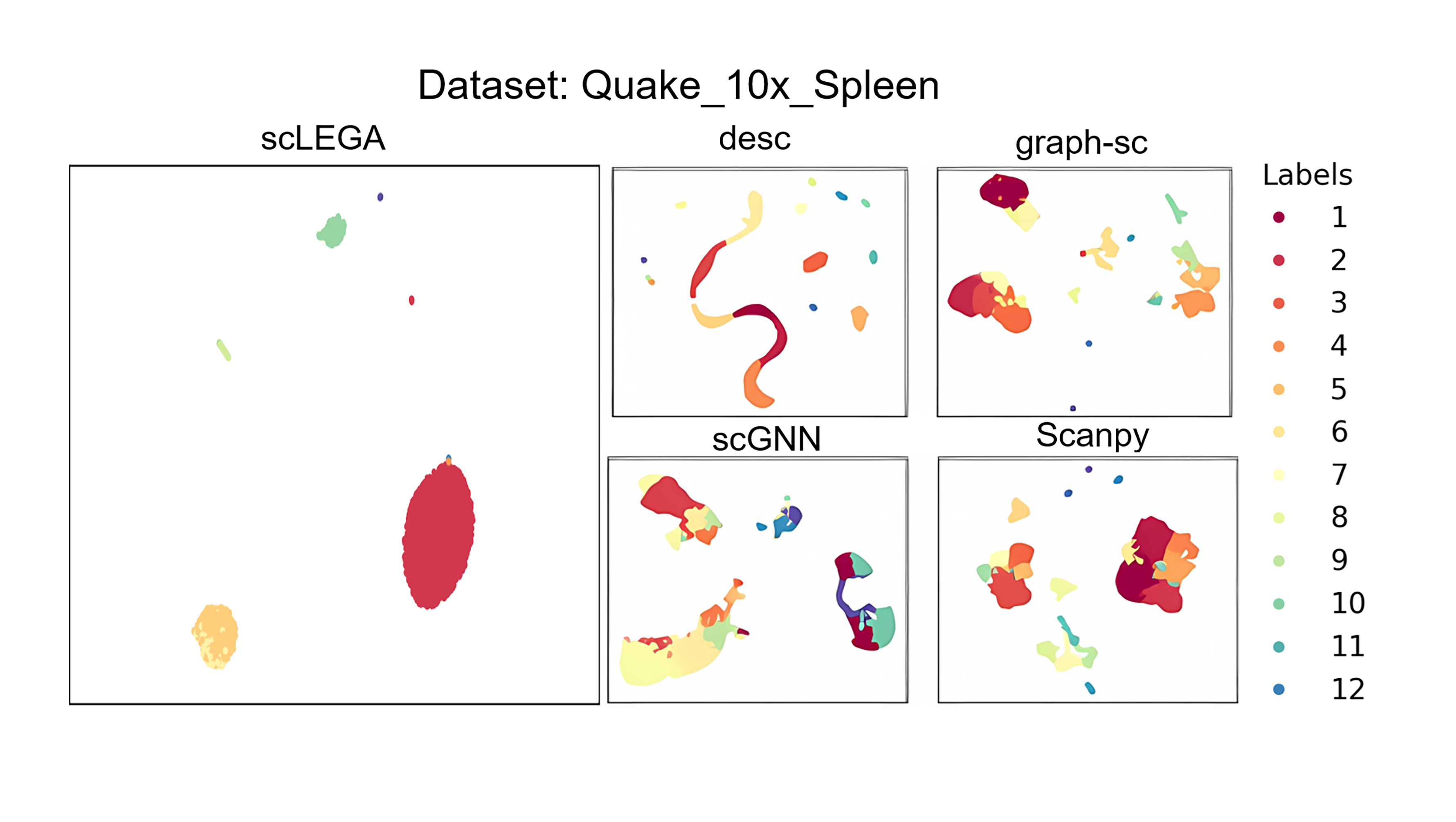

Supplement: bbae371 [file bbae371.zip › Figure S17.tif]

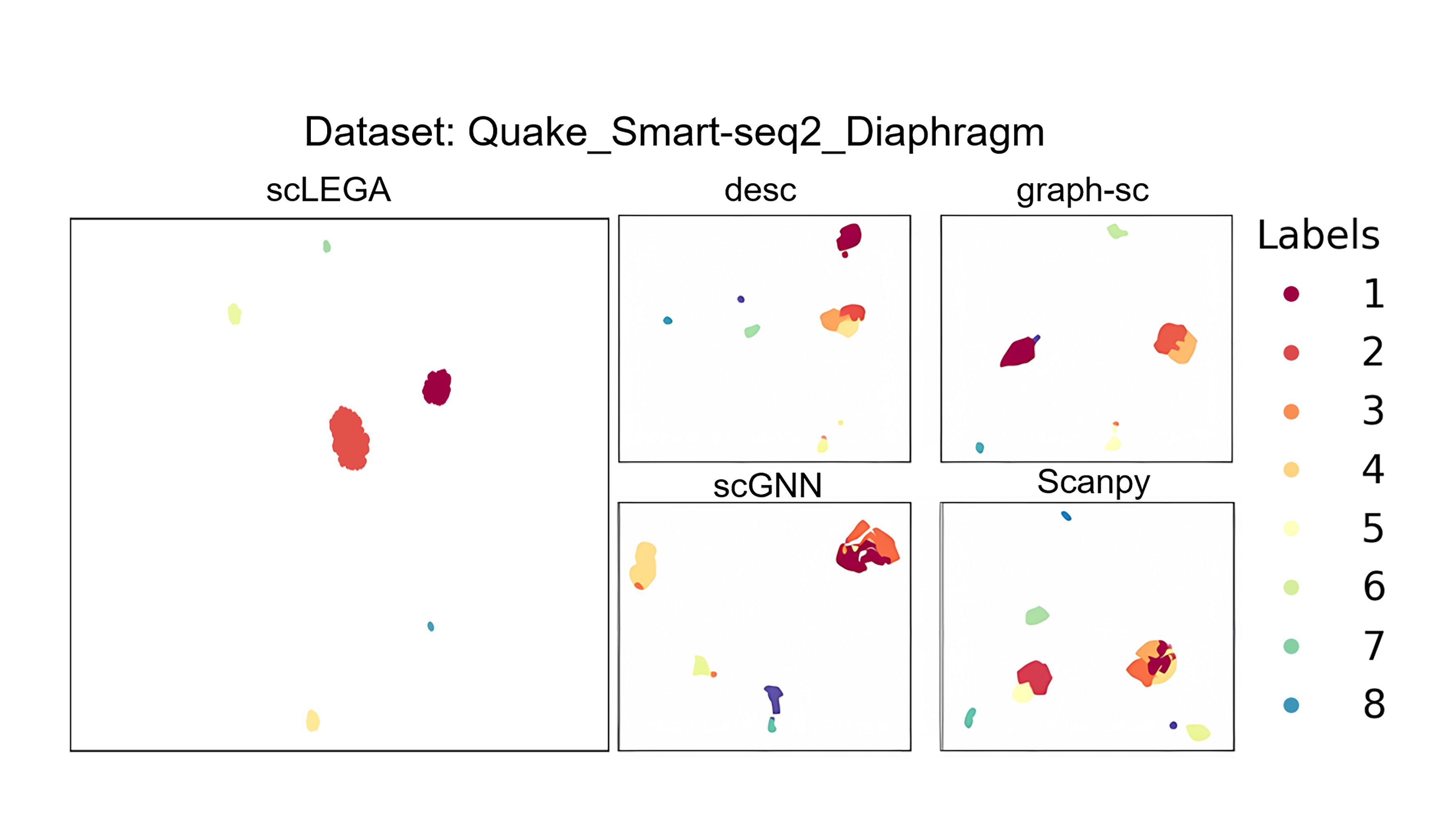

Supplement: bbae371 [file bbae371.zip › Figure S18.tif]

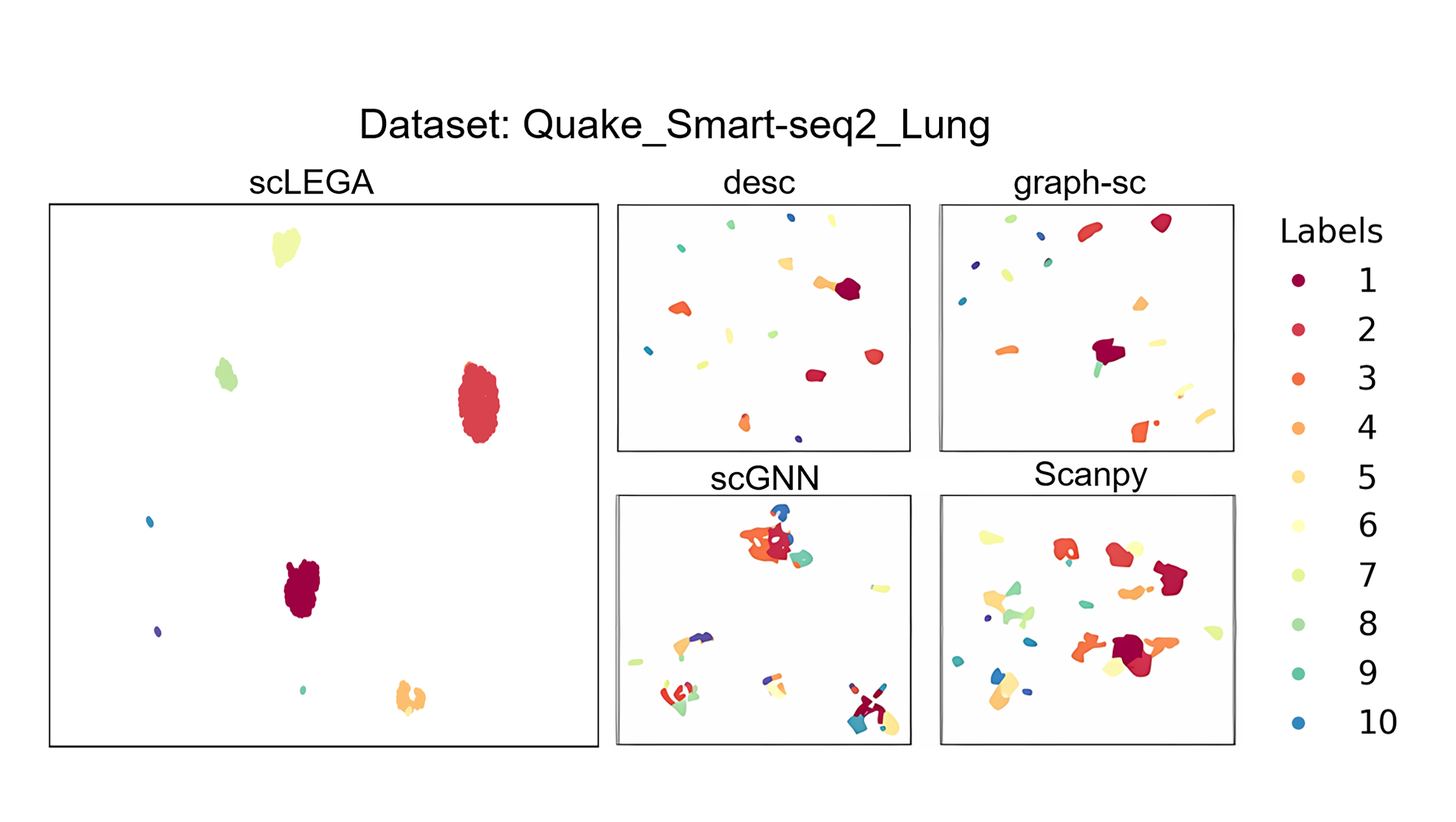

Supplement: bbae371 [file bbae371.zip › Figure S19.tif]

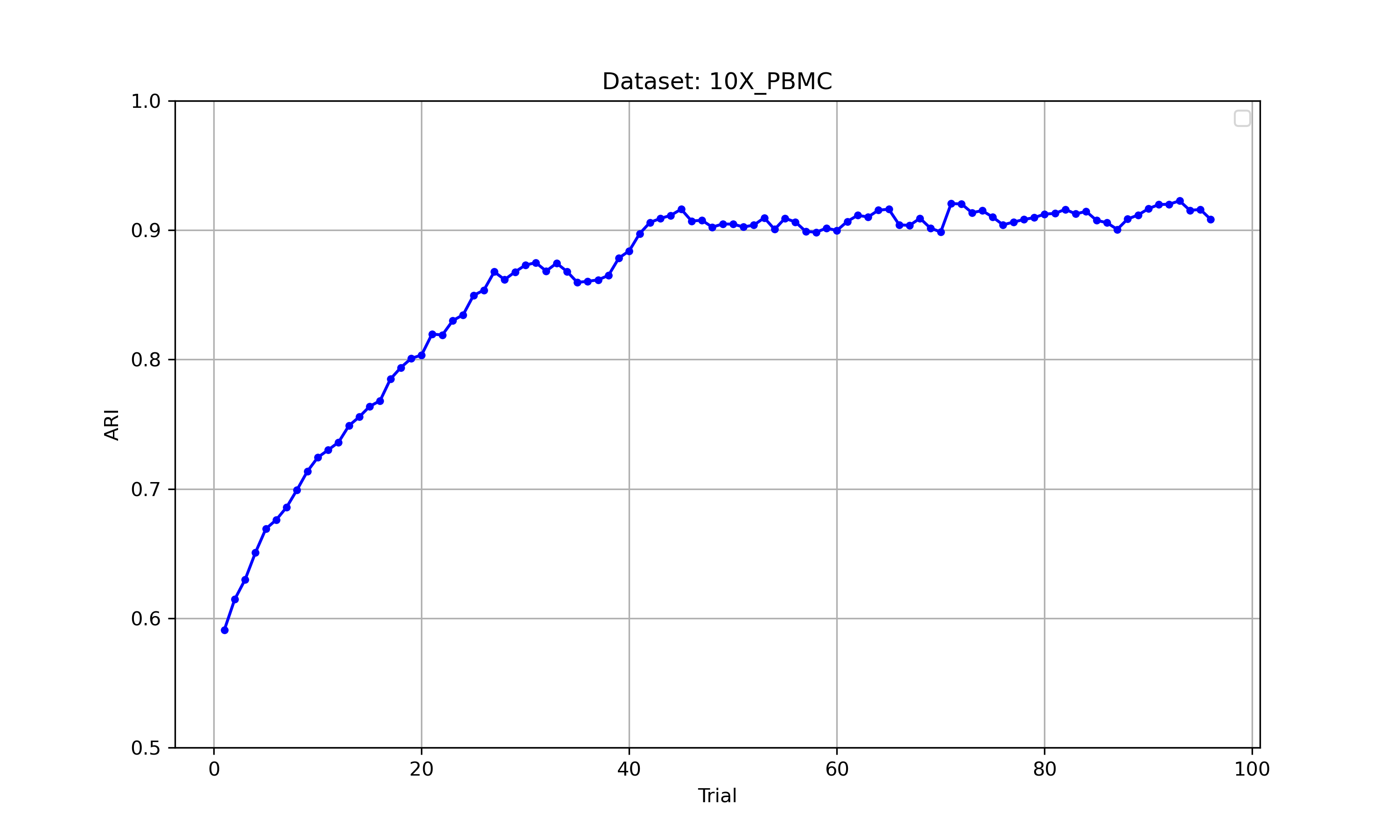

Supplement: bbae371 [file bbae371.zip › Figure S2.tif]

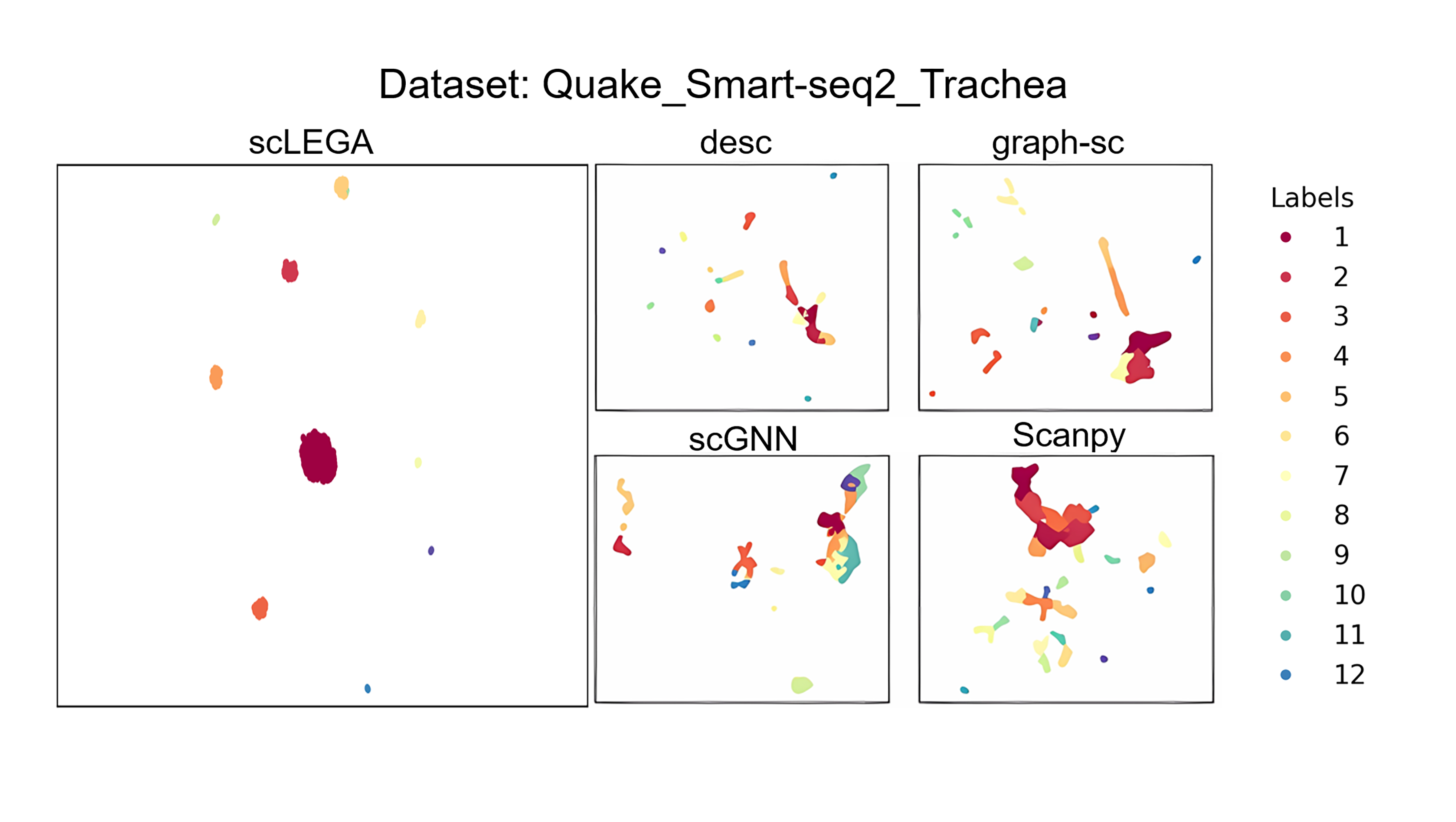

Supplement: bbae371 [file bbae371.zip › Figure S20.tif]

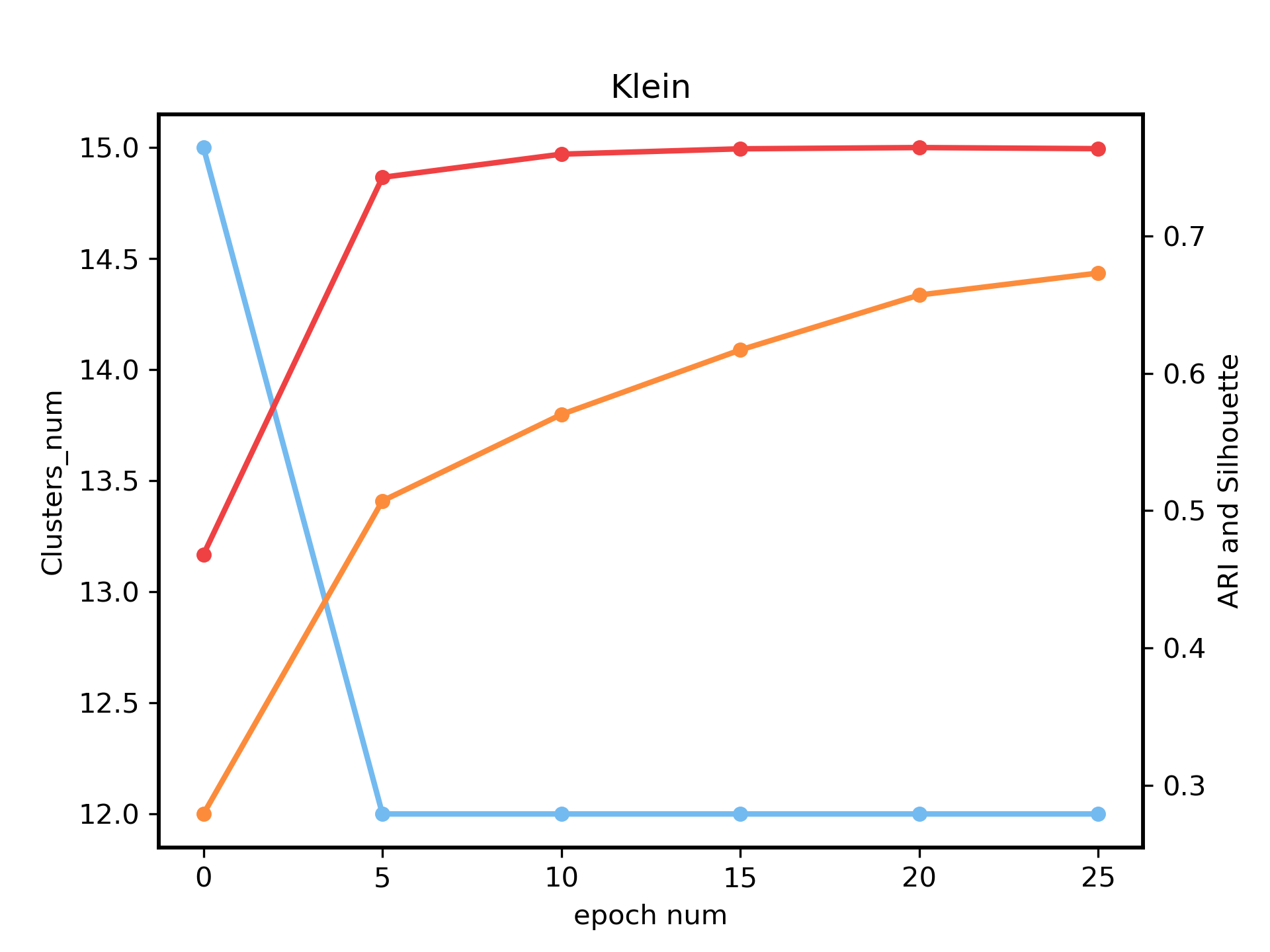

Supplement: bbae371 [file bbae371.zip › Figure S21.tif]

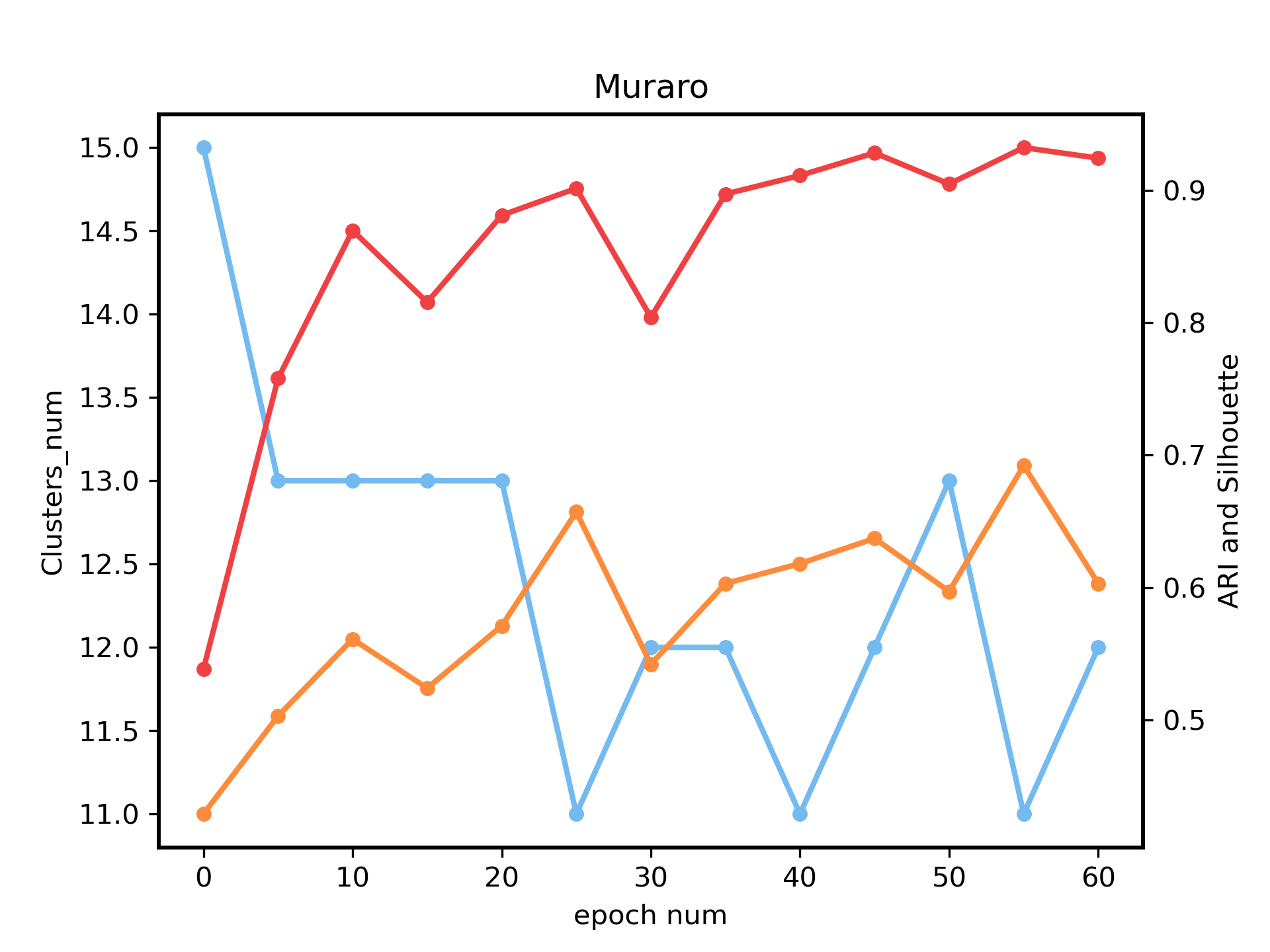

Supplement: bbae371 [file bbae371.zip › Figure S22.tif]

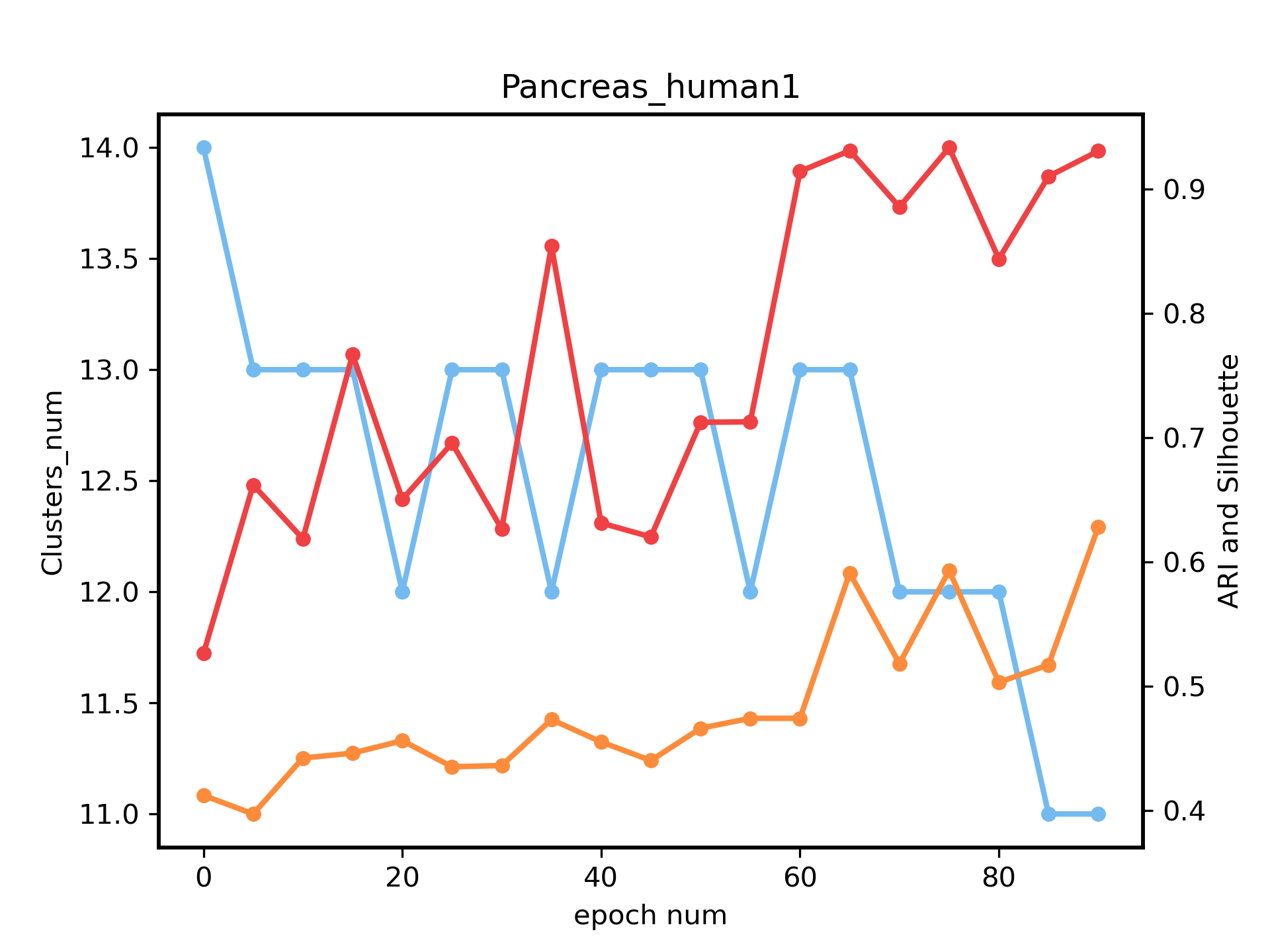

Supplement: bbae371 [file bbae371.zip › Figure S23.tif]

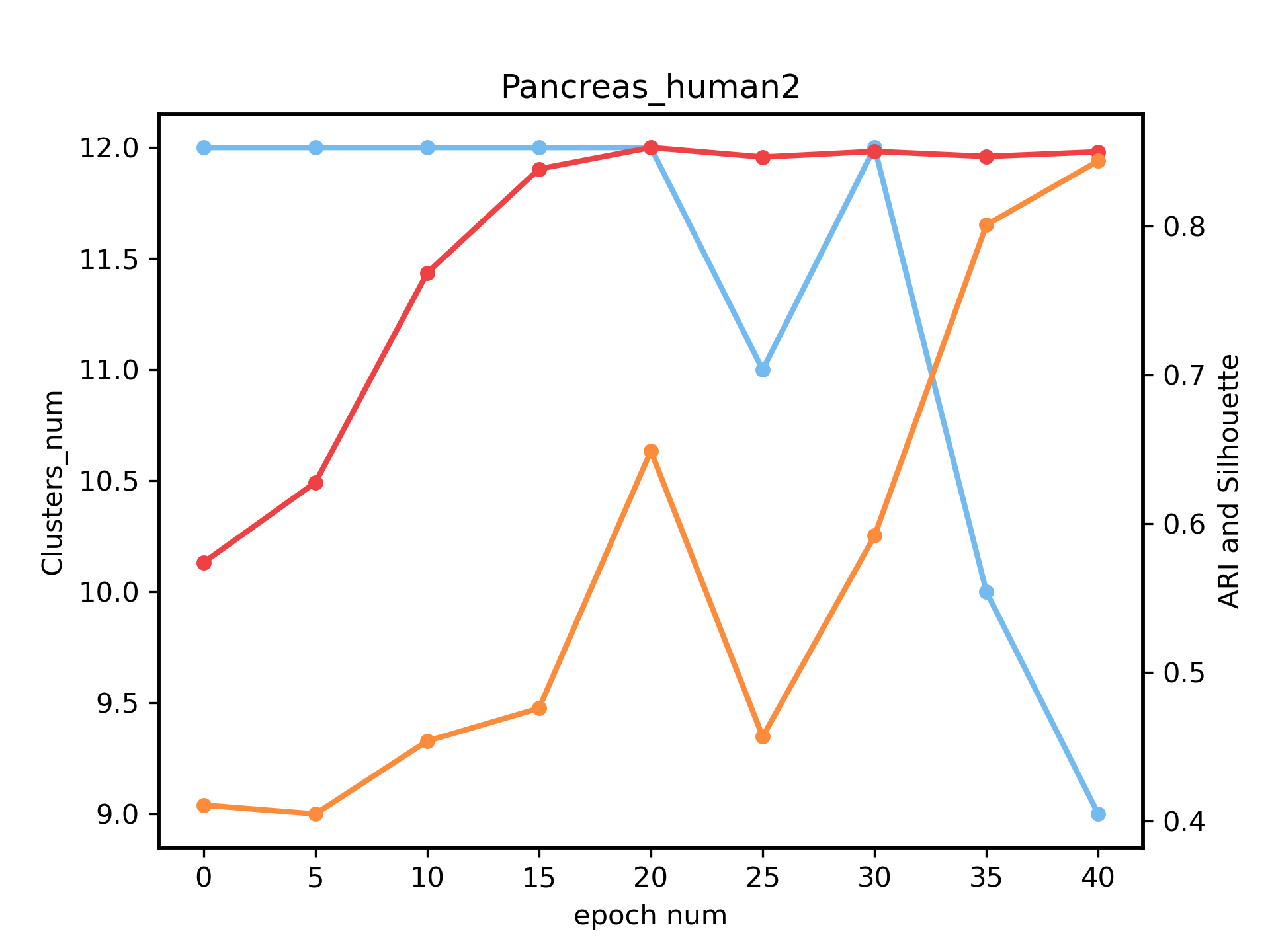

Supplement: bbae371 [file bbae371.zip › Figure S24.tif]

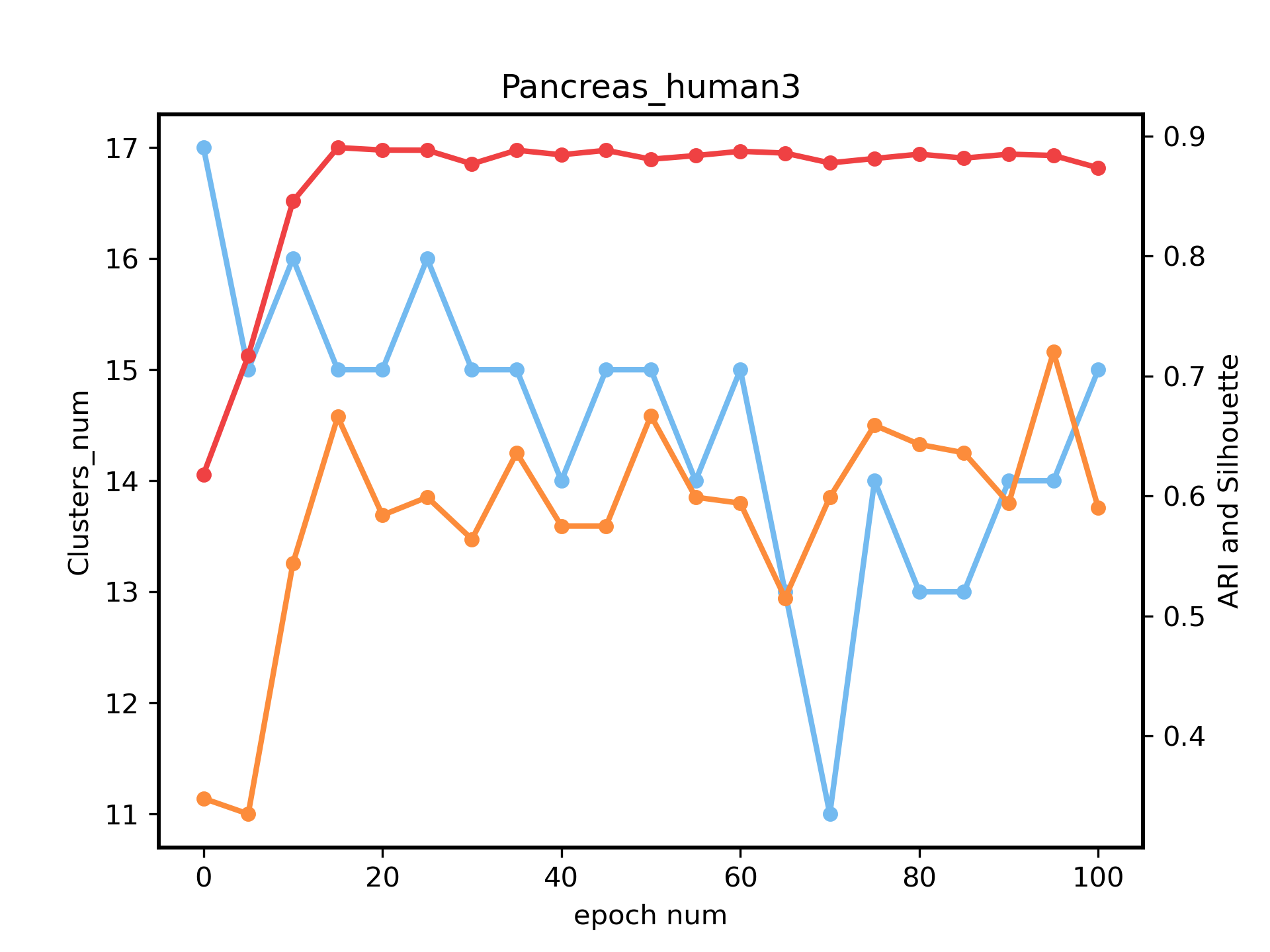

Supplement: bbae371 [file bbae371.zip › Figure S25.tif]

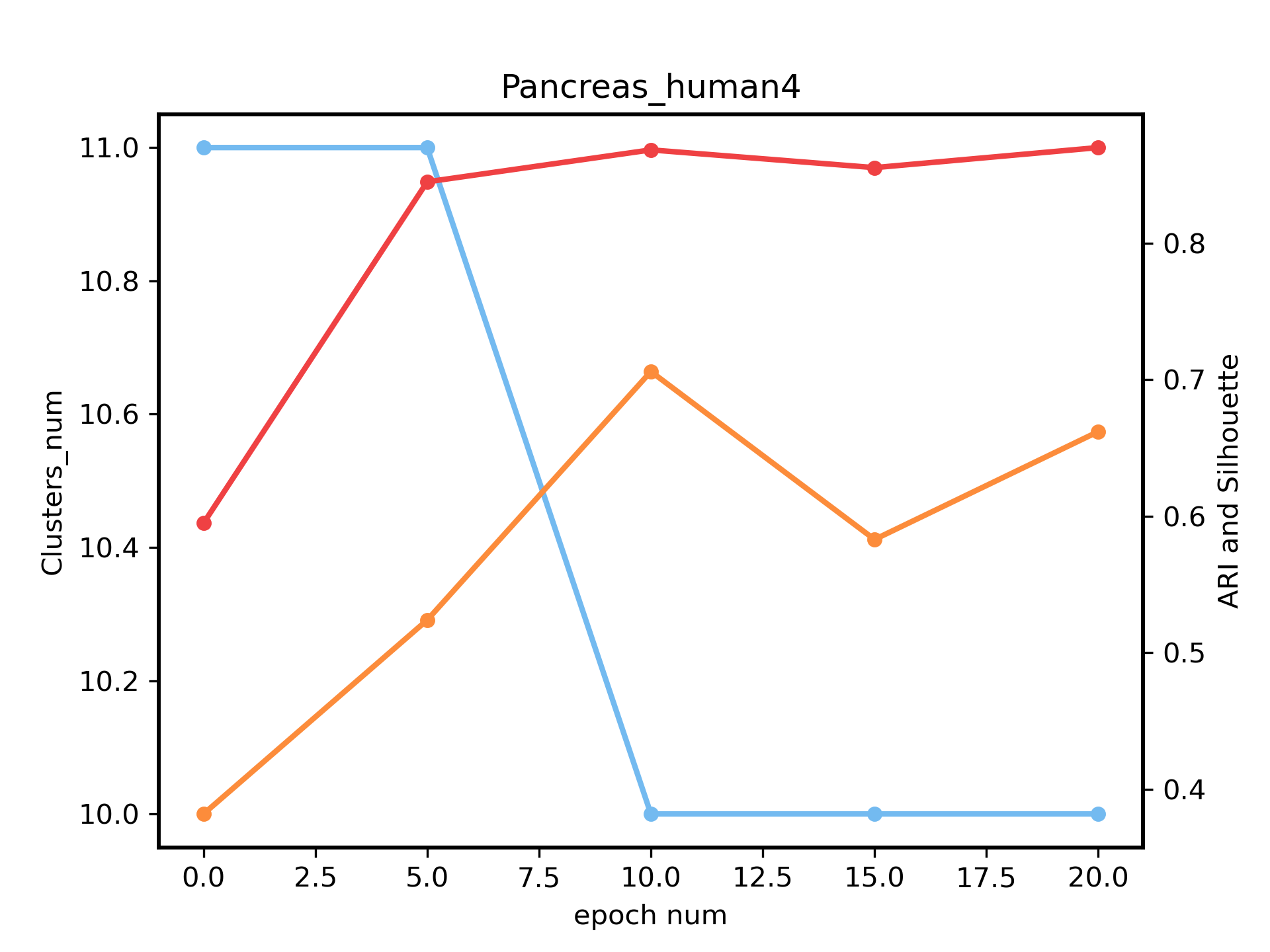

Supplement: bbae371 [file bbae371.zip › Figure S26.tif]

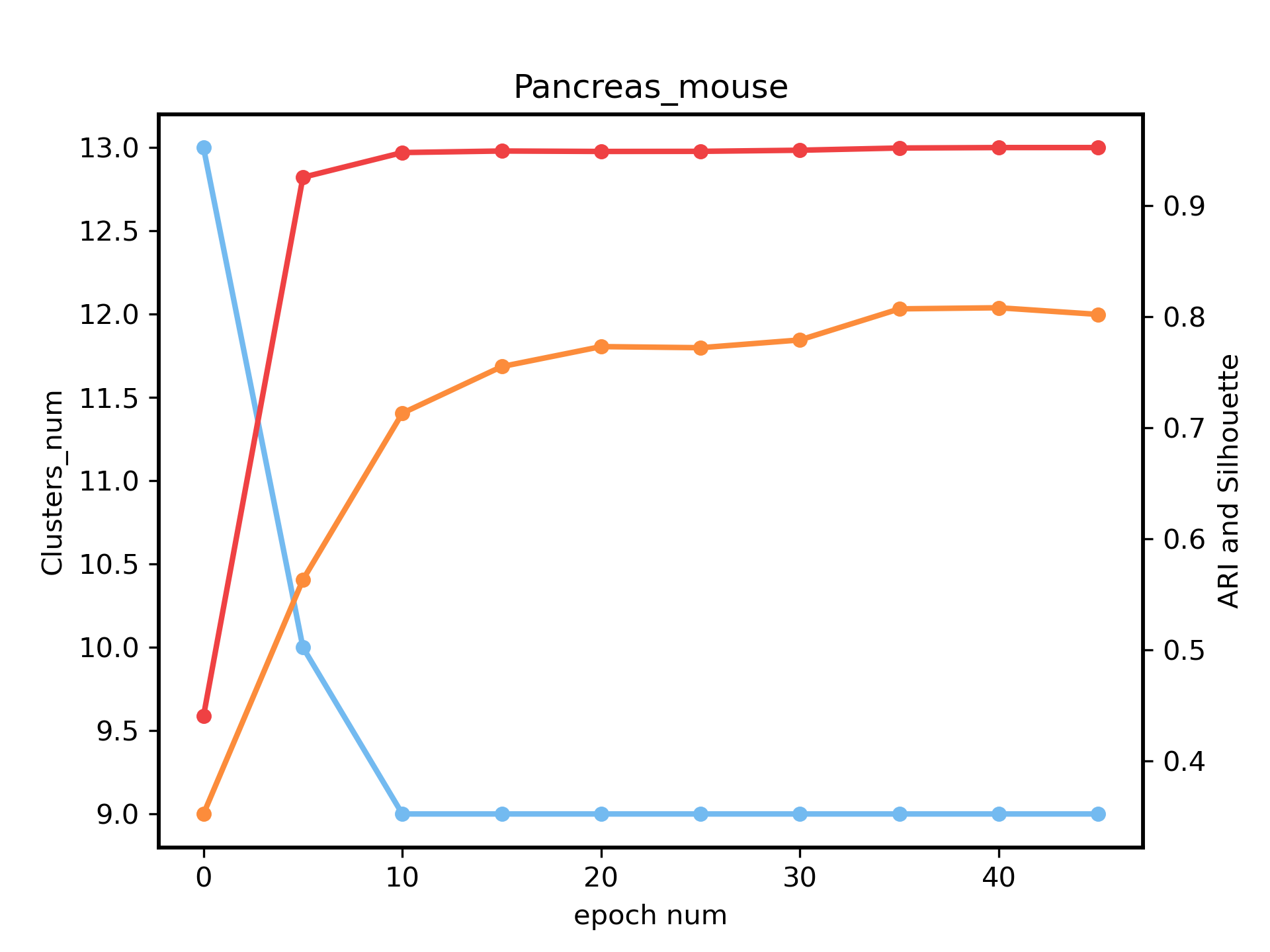

Supplement: bbae371 [file bbae371.zip › Figure S27.tif]

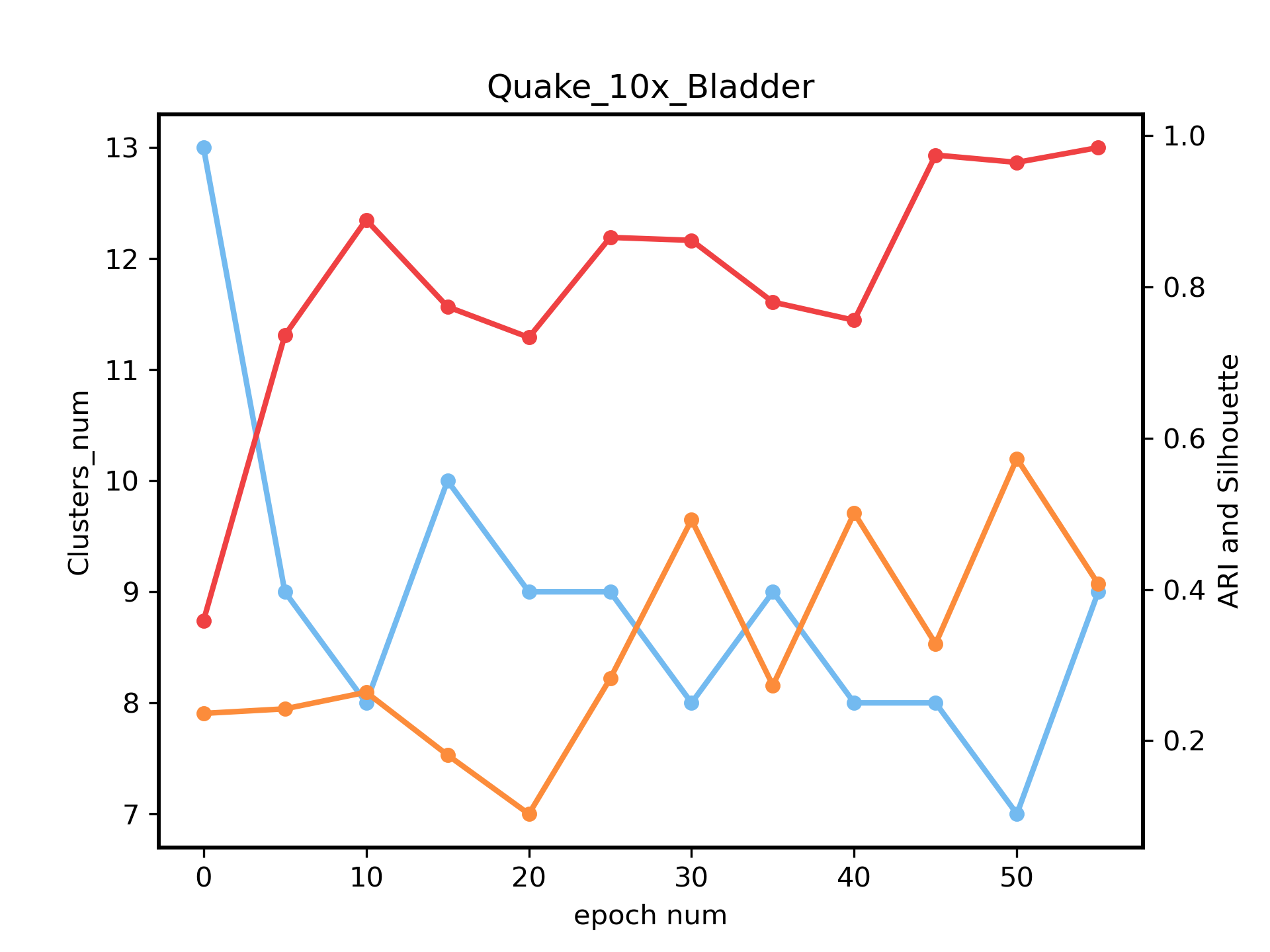

Supplement: bbae371 [file bbae371.zip › Figure S28.tif]

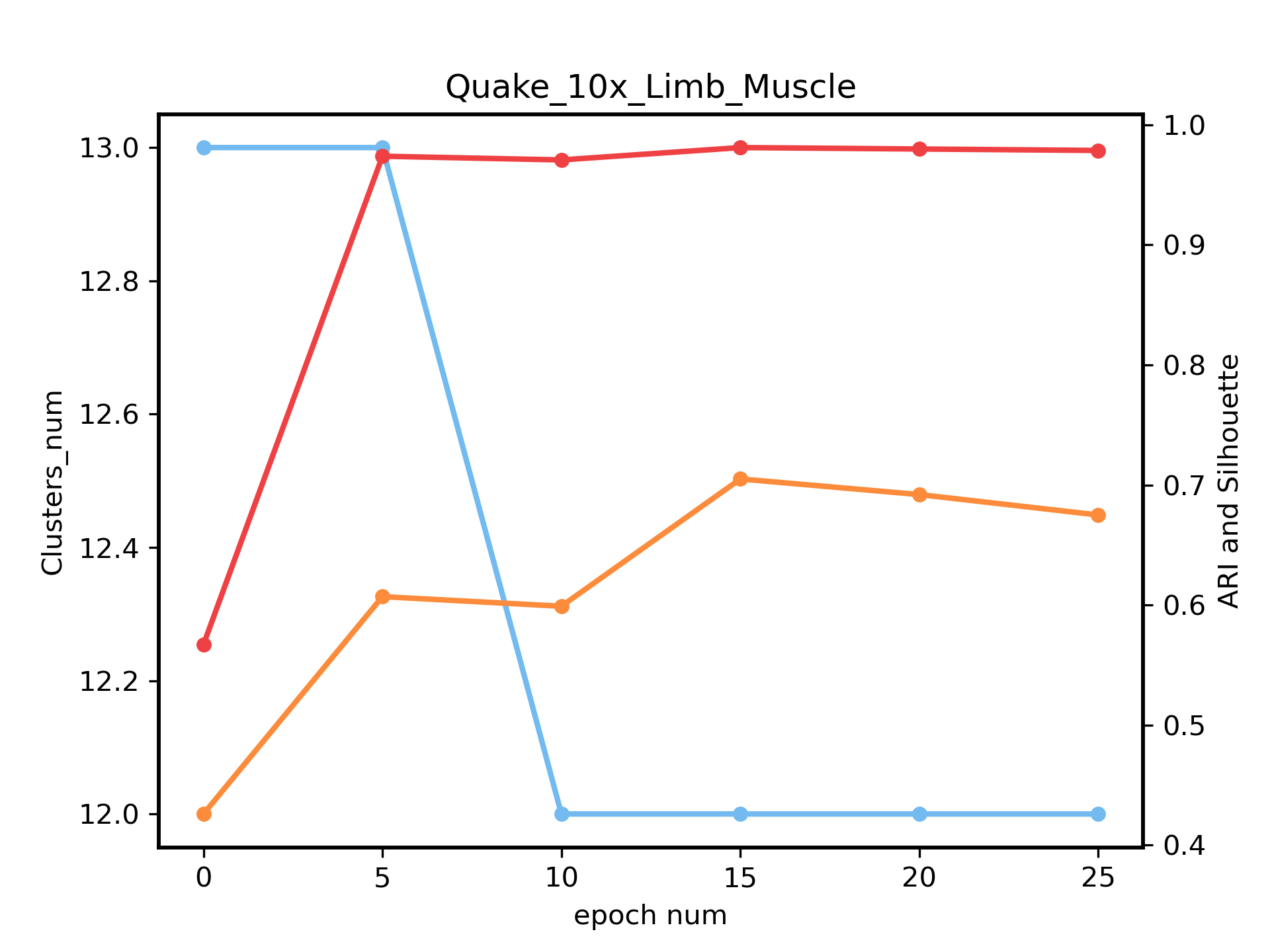

Supplement: bbae371 [file bbae371.zip › Figure S29.tif]

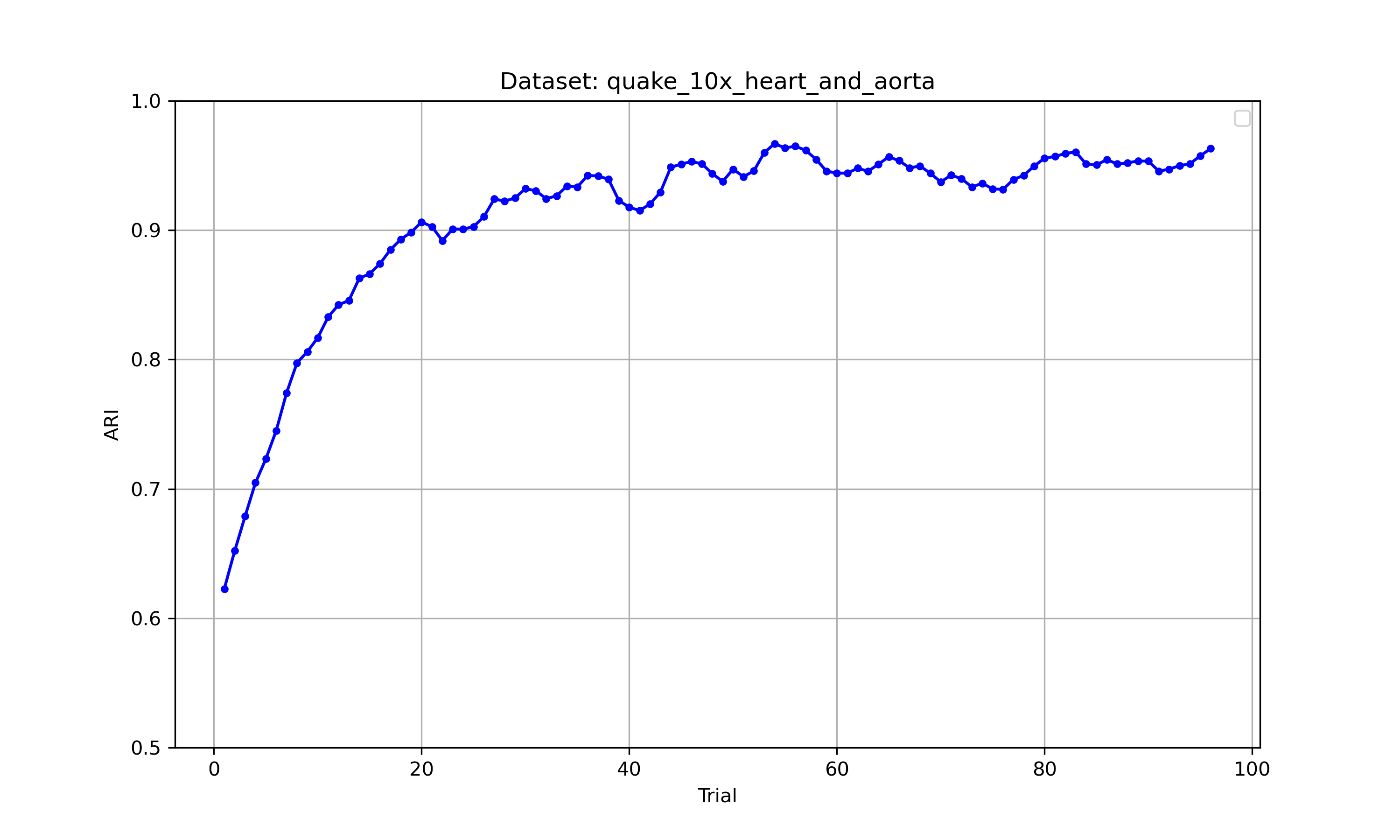

Supplement: bbae371 [file bbae371.zip › Figure S3.tif]

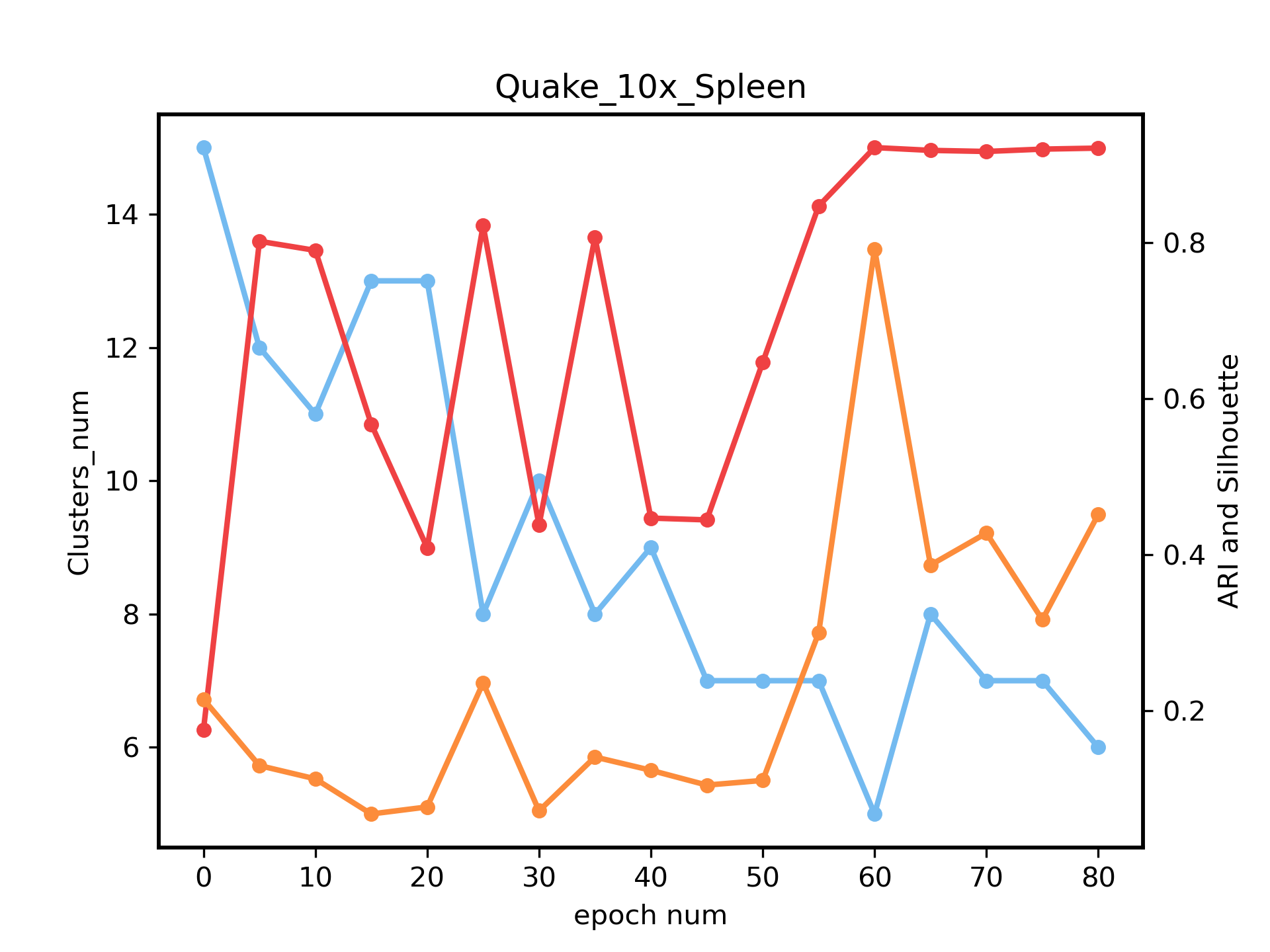

Supplement: bbae371 [file bbae371.zip › Figure S30.tif]

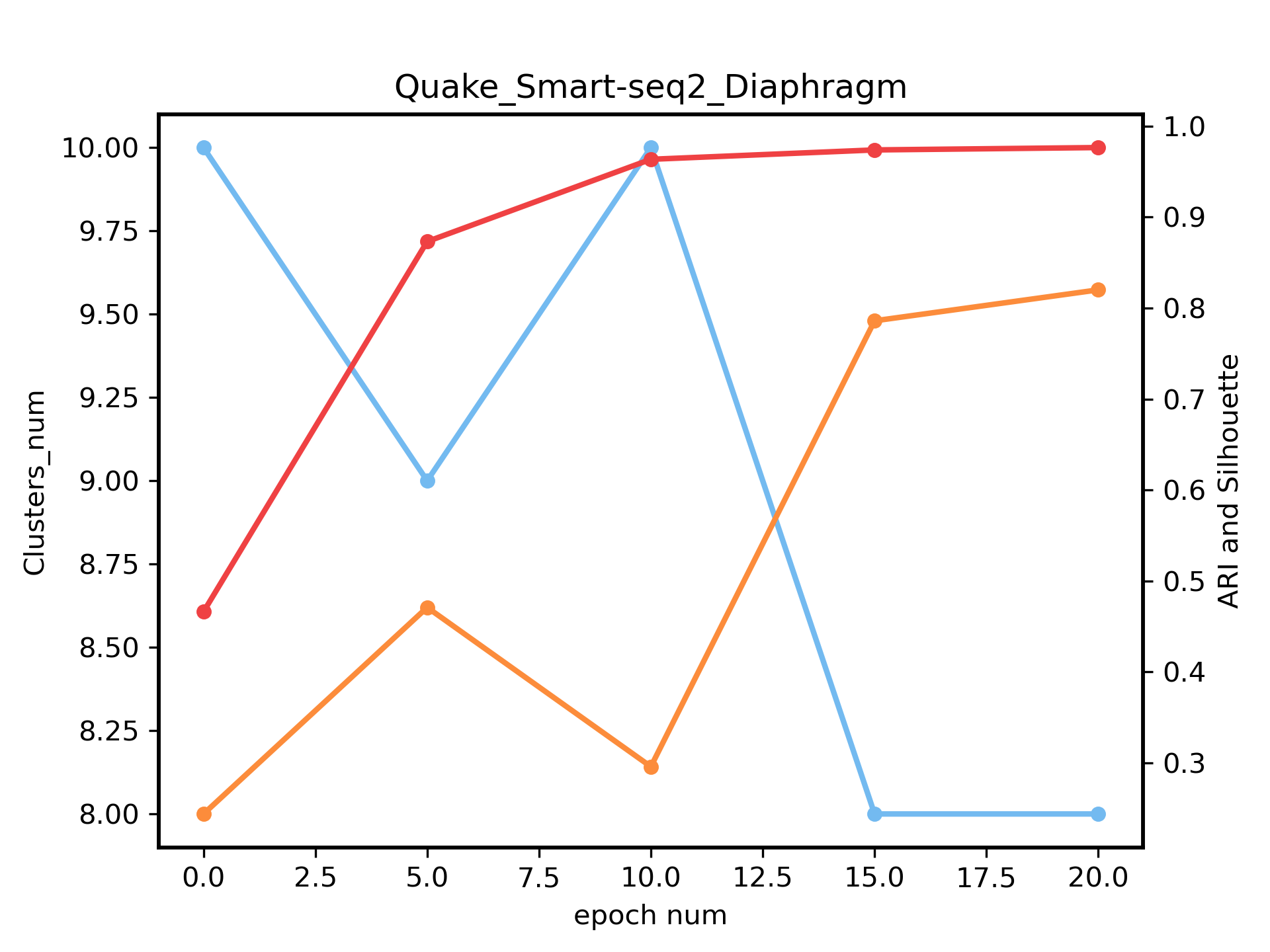

Supplement: bbae371 [file bbae371.zip › Figure S31.tif]

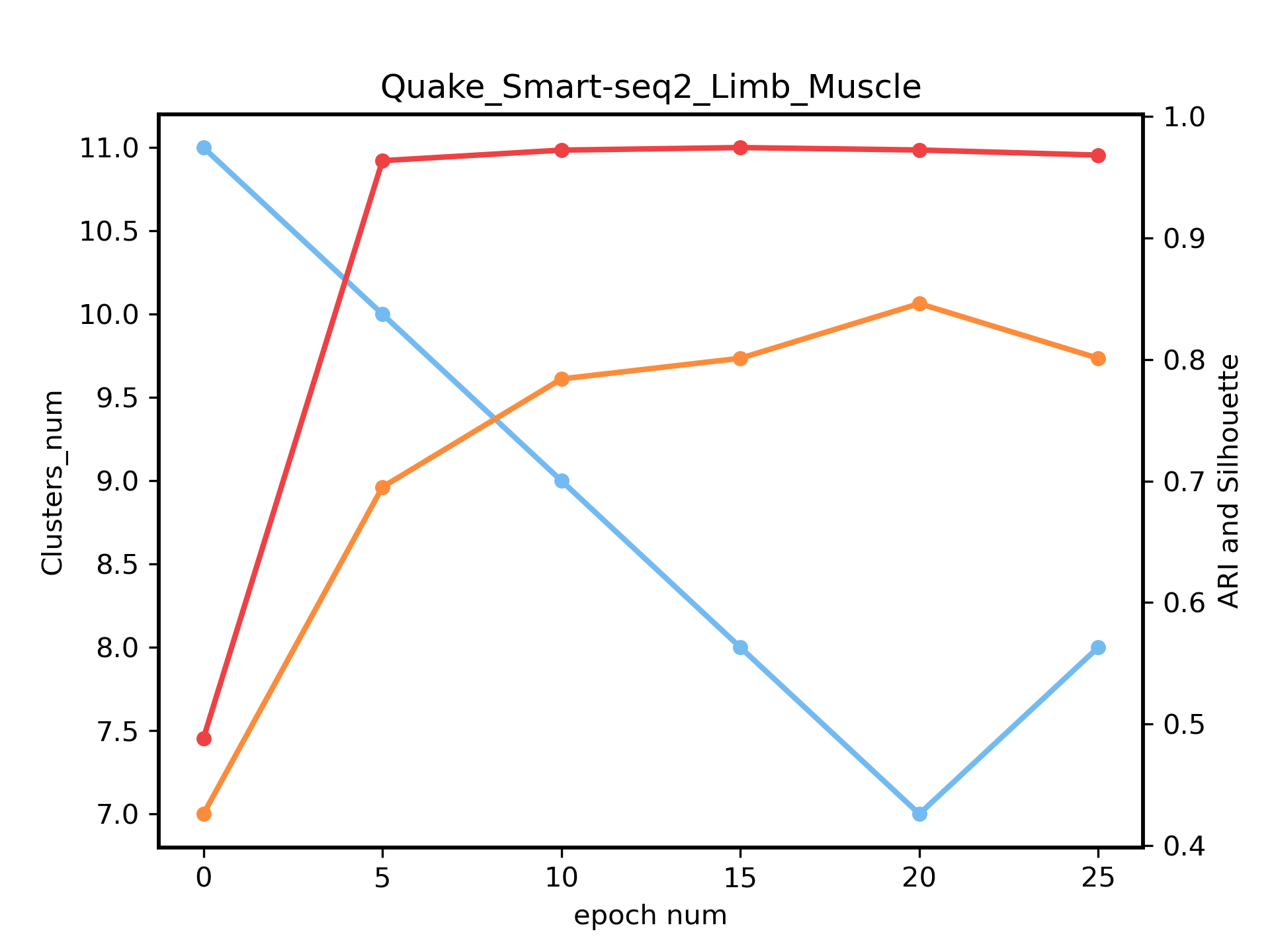

Supplement: bbae371 [file bbae371.zip › Figure S32.tif]

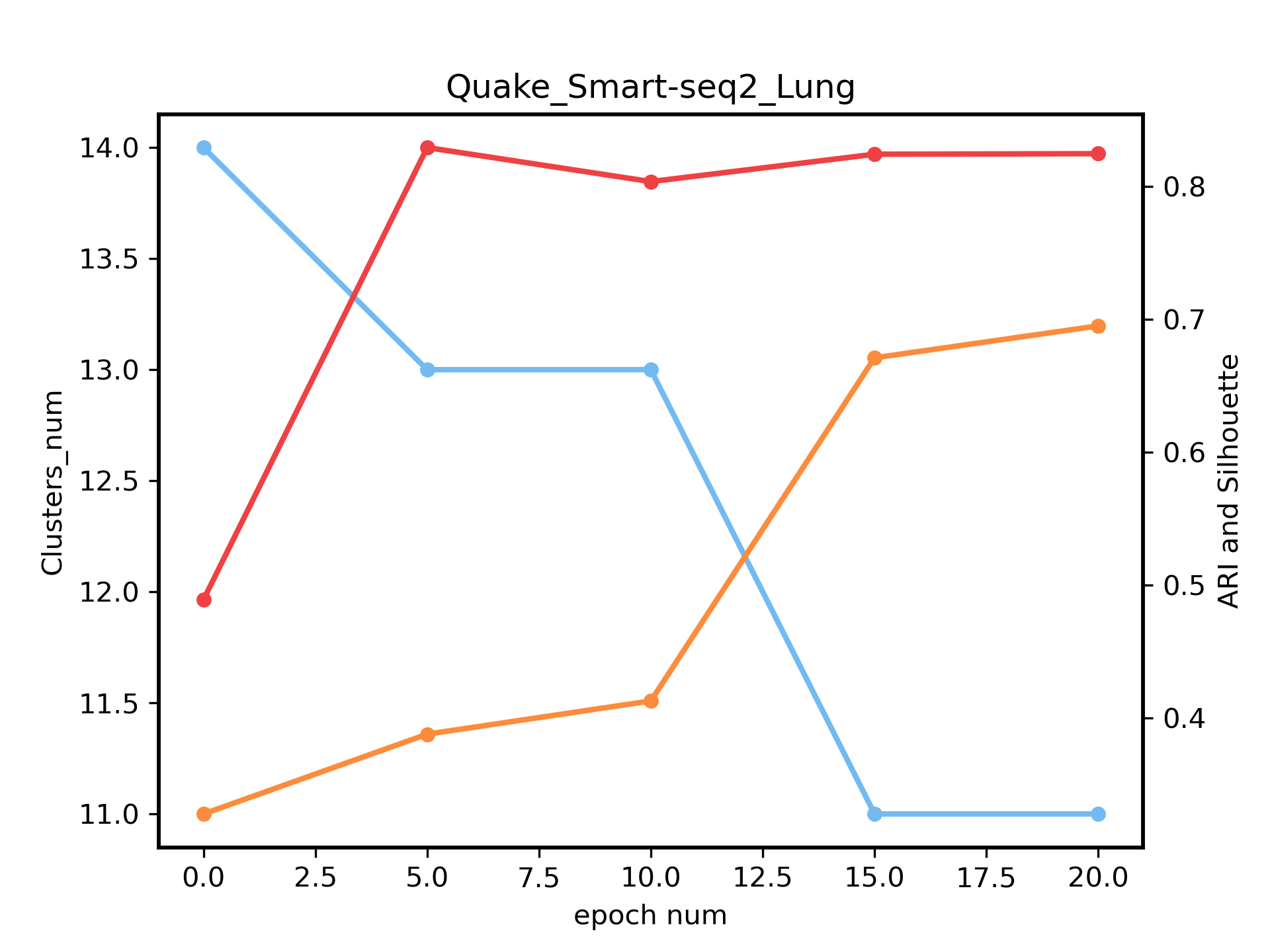

Supplement: bbae371 [file bbae371.zip › Figure S33.tif]

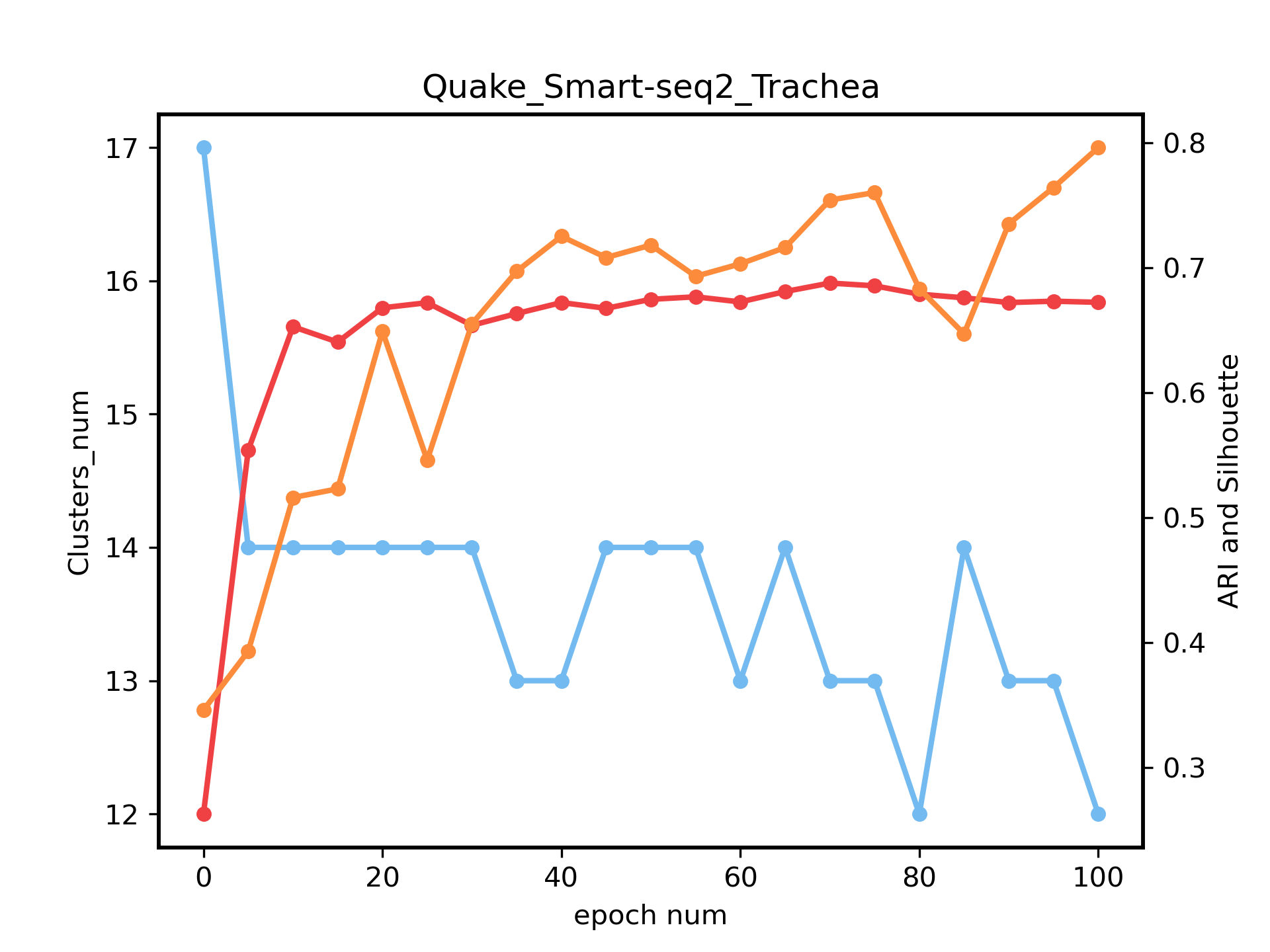

Supplement: bbae371 [file bbae371.zip › Figure S34.tif]

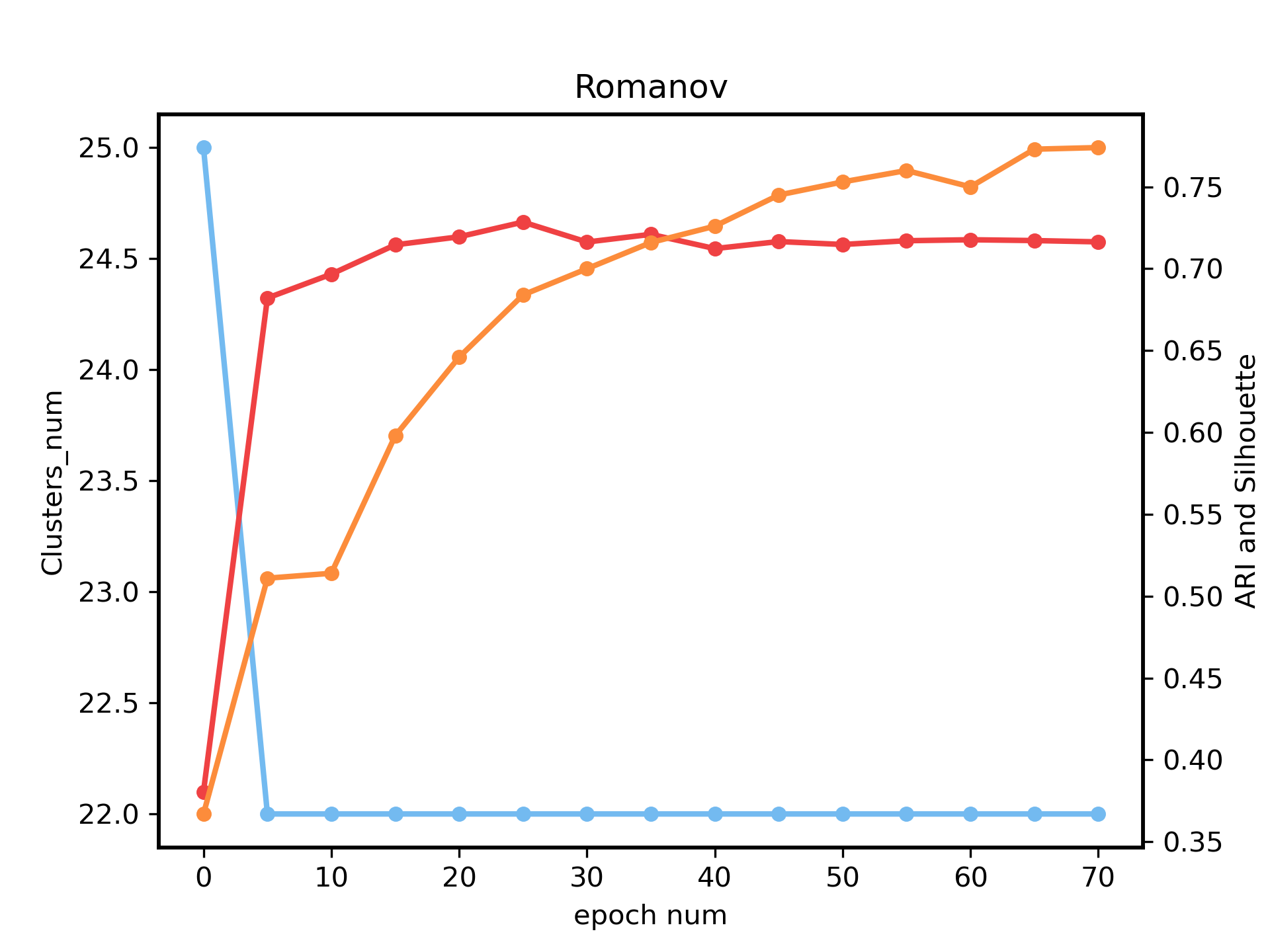

Supplement: bbae371 [file bbae371.zip › Figure S35.tif]

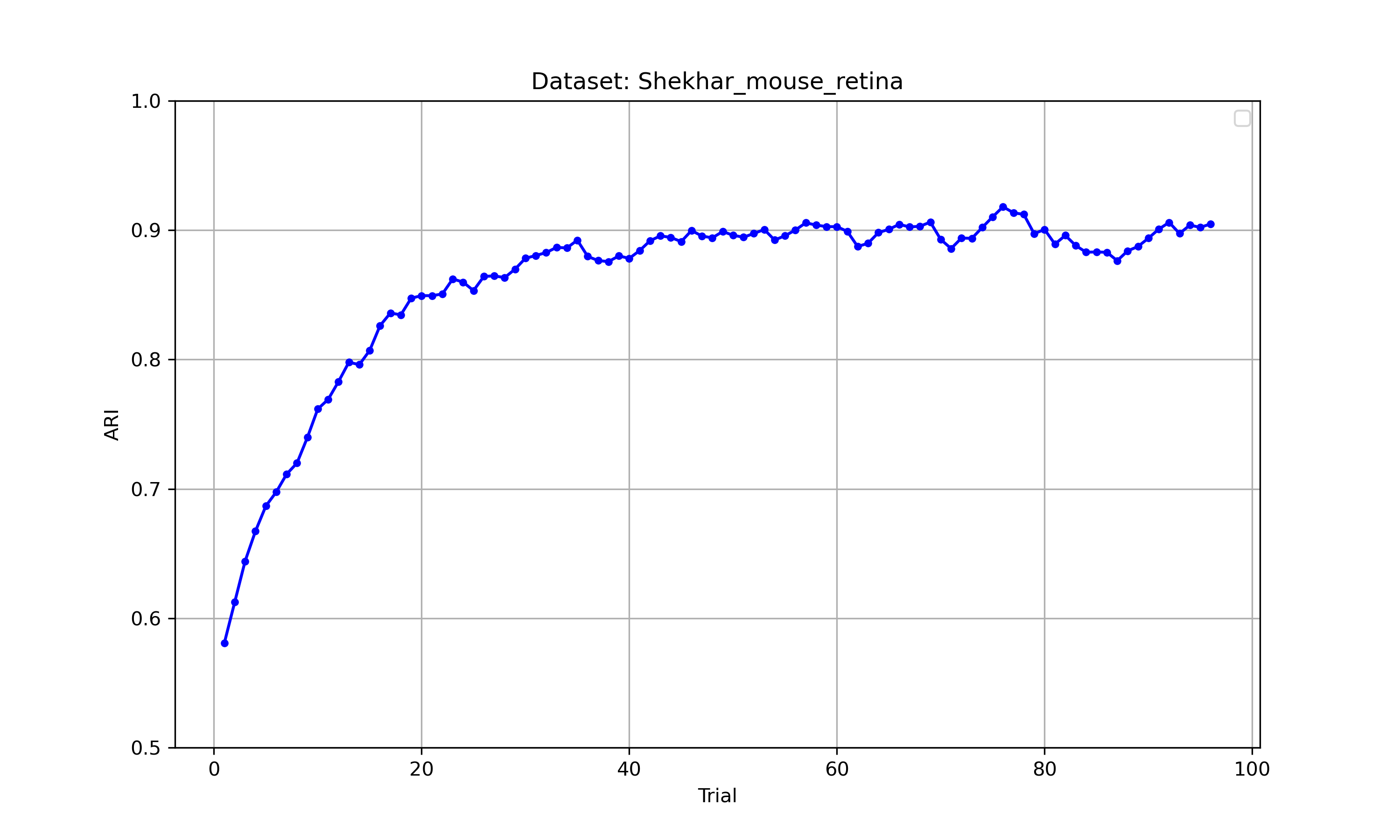

Supplement: bbae371 [file bbae371.zip › Figure S4.tif]

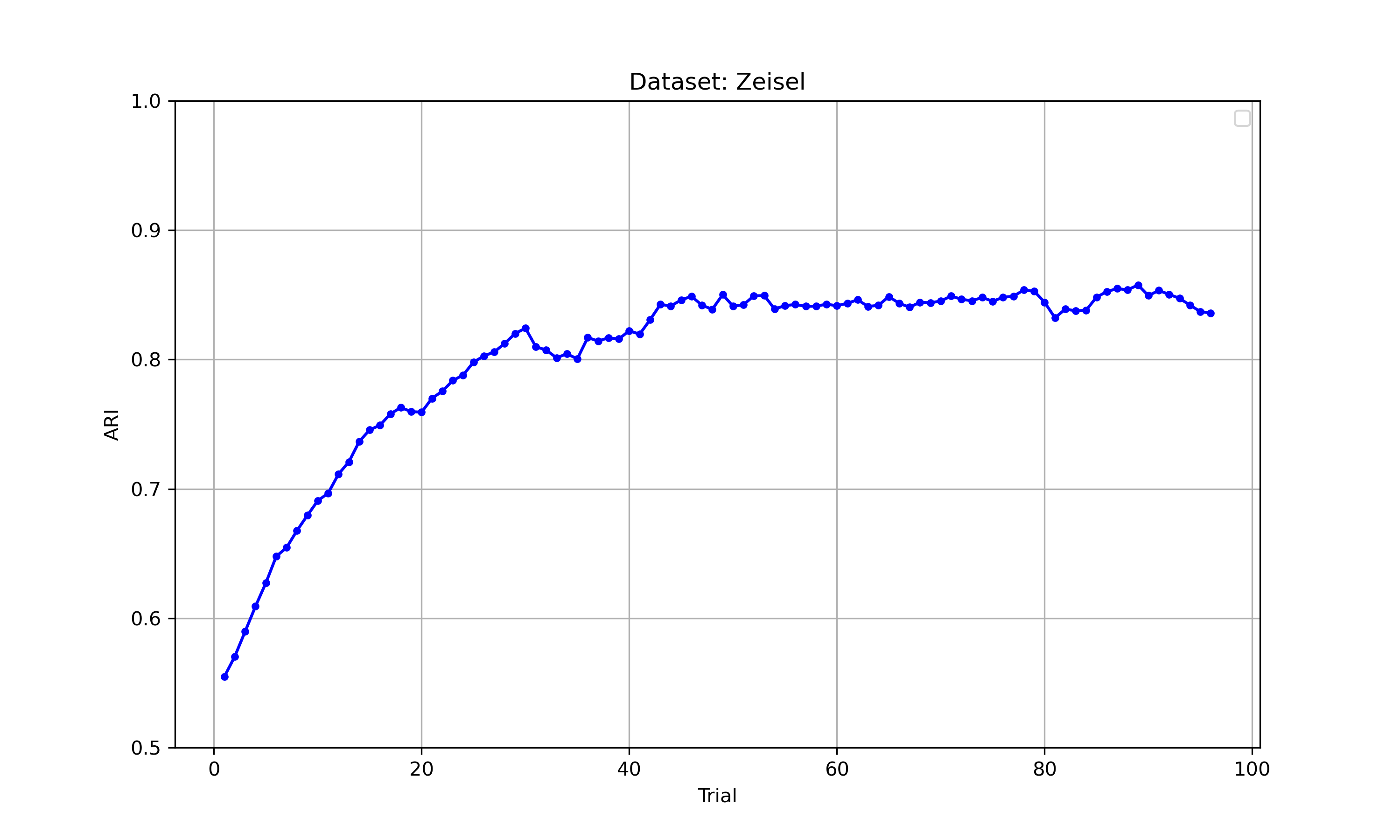

Supplement: bbae371 [file bbae371.zip › Figure S5.tif]

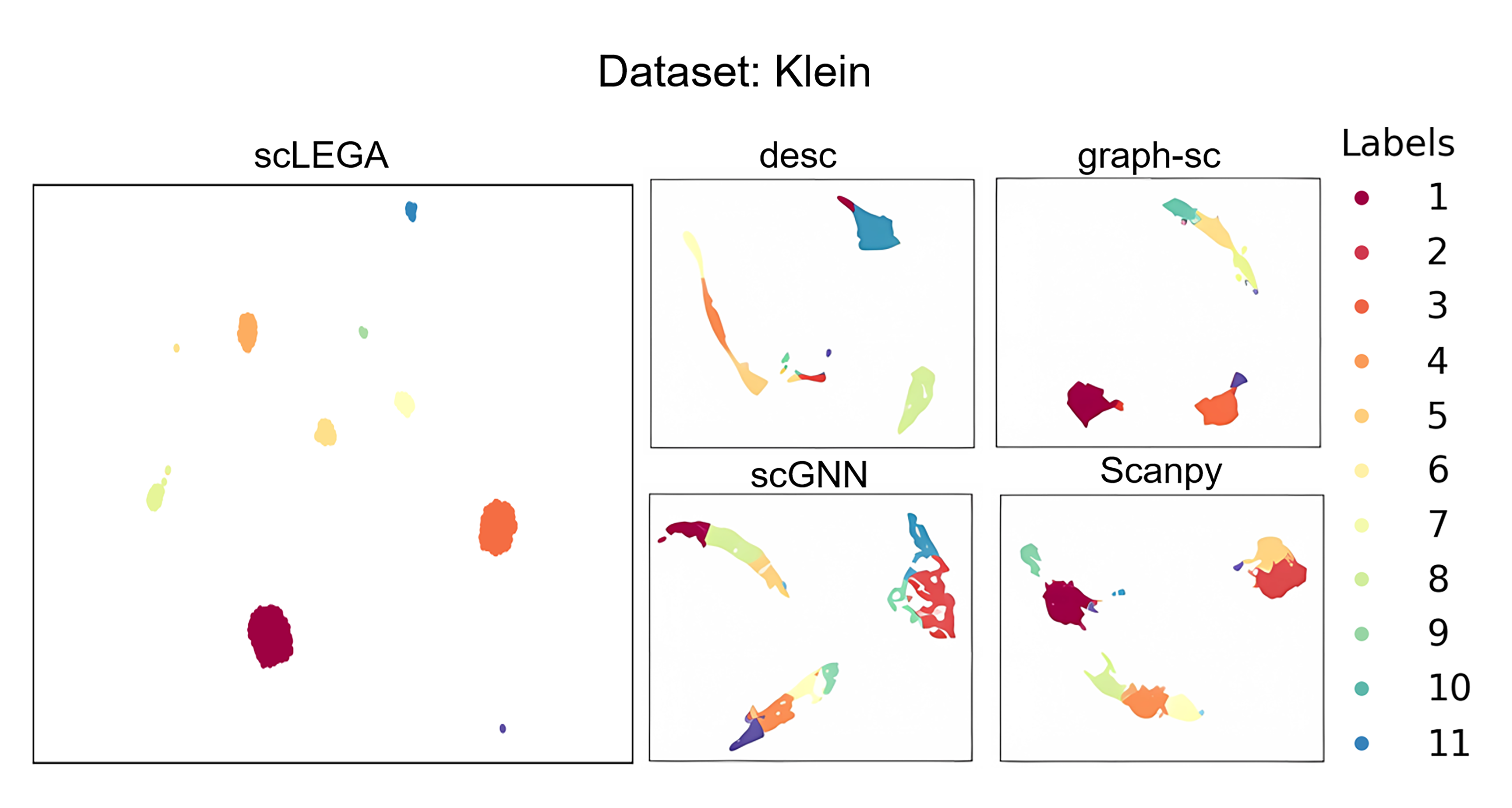

Supplement: bbae371 [file bbae371.zip › Figure S6.tif]

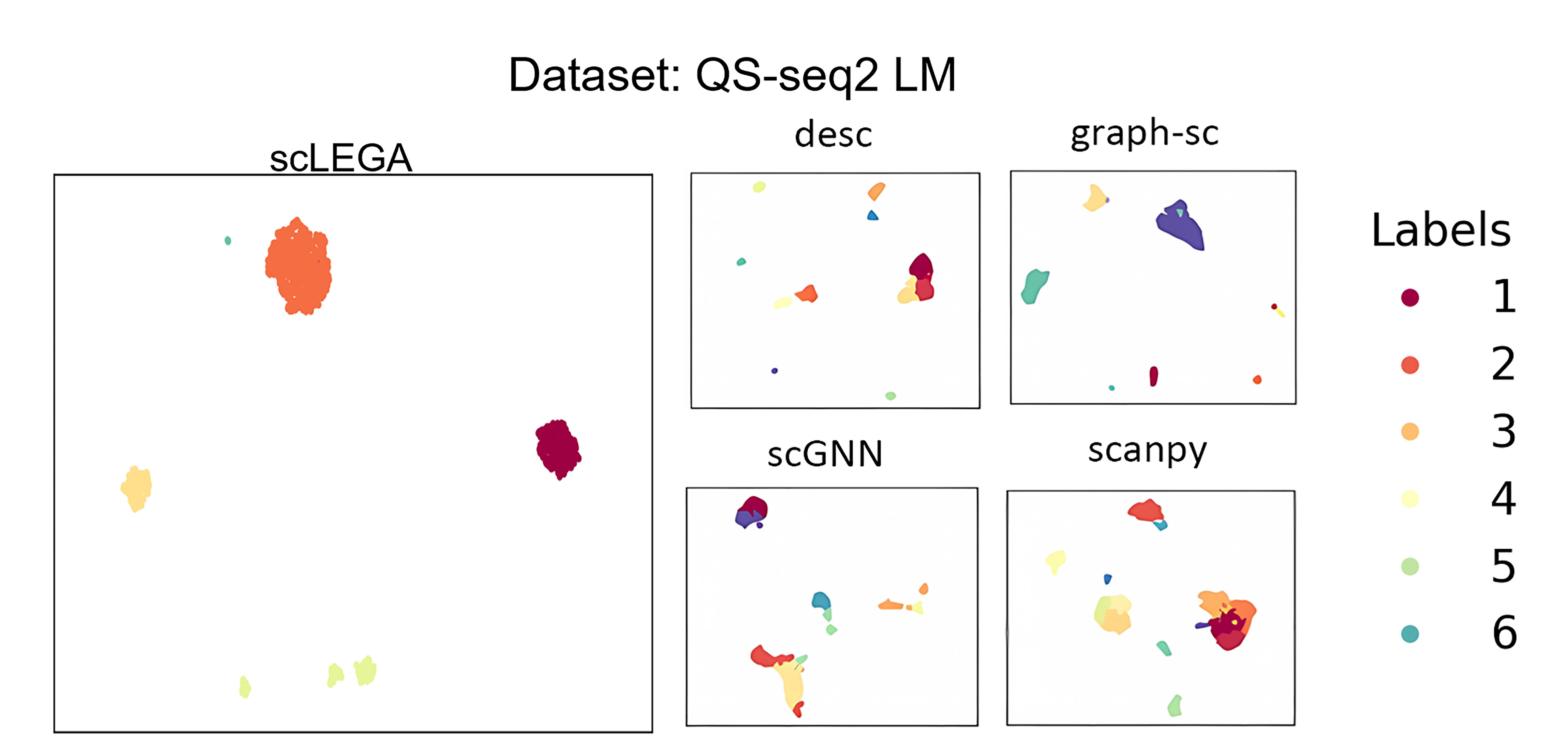

Supplement: bbae371 [file bbae371.zip › Figure S7.tif]

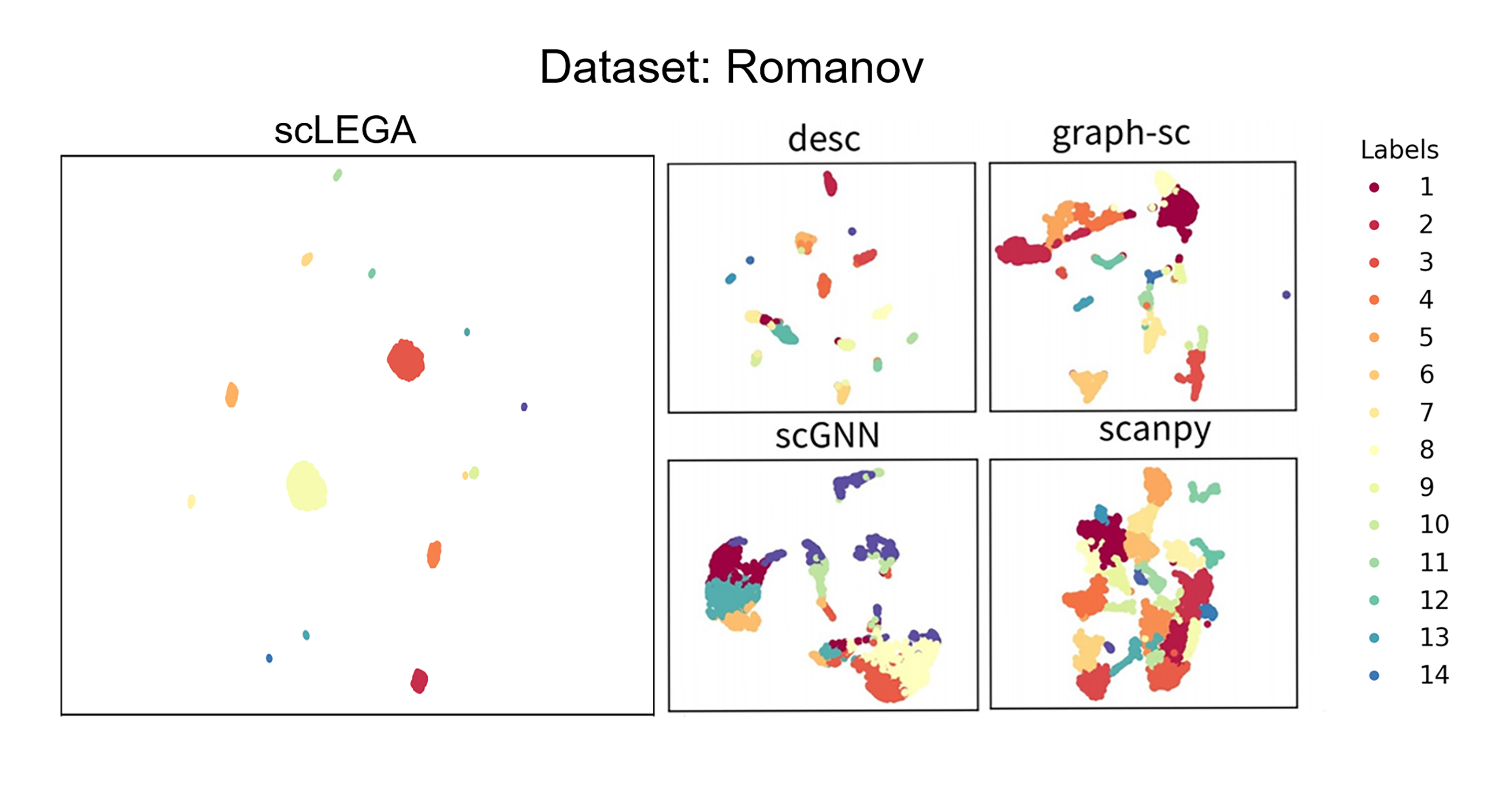

Supplement: bbae371 [file bbae371.zip › Figure S8.tif]

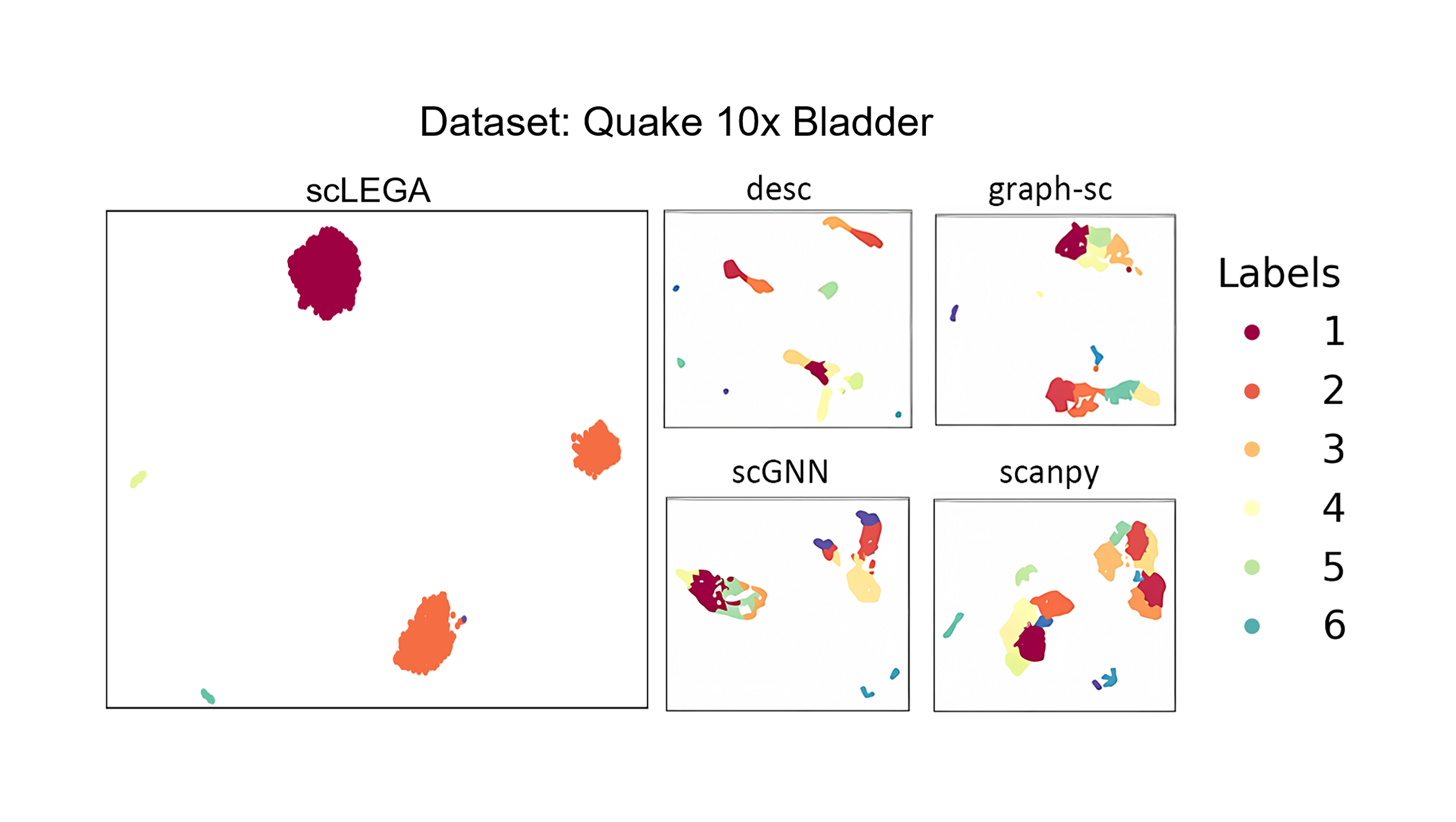

Supplement: bbae371 [file bbae371.zip › Figure S9.tif]
